# Orphan GPR84 facilitates uropod de-adhesion at multiple steps during leukocyte extravasation

**DOI:** 10.1038/s44319-026-00923-0

**Published:** 2026-09-12

**Authors:** Clare Latta, Terrence M Trinca, Francesca Robertson, Rachel Lau, Anna Barkaway, Loïc Rolas, Matthew Golding, Paul R C Imbert, Haitao Wang, Almke Bader, Liam S Hill, Lorna Hodgson, Yue Yang, Aleksandar Ivetic, Barbara Walzog, Myles Lewis, Mathieu-Benoit Voisin, Paul Martin, Sussan Nourshargh, Helen Weavers

**Affiliations:** 1 Centre for Microvascular Research, William Harvey Research Institute, Faculty of Medicine and Dentistry, Queen Mary University of London, London, UK; 2 School of Biochemistry, Biomedical Sciences Building, University of Bristol, Bristol, UK; 3 Experimental Medicine and Rheumatology, William Harvey Research Institute, Faculty of Medicine and Dentistry, Queen Mary University of London, London, UK; 4 Institute of Cardiovascular Physiology and Pathophysiology, Biomedical Center, Ludwig-Maximilians-Universität München, Munich, Germany; 5 School of Cardiovascular and Metabolic Medicine and Sciences, James Black Centre, BHF Centre of Research Excellence, King’s College London, London, UK

**Keywords:** Cell Adhesion, Polarity & Cytoskeleton, Immunology, Signal Transduction

## Abstract

Leukocyte migration through venular walls is an essential component of effective immunity. While the key molecular players driving the initial steps of this response are known, the terminating signals remain unclear. Here, we identify a conserved role for GPR84 family GPCRs in the successful completion of the final stages of leukocyte extravasation through acutely inflamed vessels. The integration of high-resolution intravital imaging with cell-specific genetics reveals that the deficiency of GPR84 orthologs in mice and *Drosophila* results in flawed detachment of transmigrating immune cells from vessel walls. Mechanistically, transcriptomics reveals that GPR84-deficient neutrophils exhibit defective actin cytoskeletal regulation and cellular adhesion/de-adhesion. Consistent with this, our fly-murine pipeline shows that GPR84 supports localized and dynamic Rho activation to enable detachment of the immune cell uropod from vessel exit sites. Moreover, pharmacological blockade of GPR84 signaling dampens immune cell migration in multiple murine acute inflammatory settings. Collectively, our findings present GPR84 as a novel physiological regulator of immune cell extravasation that is amenable to therapeutic targeting for modulating leukocyte infiltration into inflamed tissues.

## Synopsis

GPCRs of the GPR84 family are key regulators of leukocyte extravasation. *Drosophila* and murine in vivo models show that leukocyte GPR84/Tre-1 support dynamic Rho-dependent detachment of stretched uropods from vessels.

- Murine and *Drosophila* GPCRs of the GPR84 family play a key role in effective leukocyte extravasation.
- GPR84/Tre1 regulate Rho-dependent detachment of leukocytes from vessels.
- Pharmacological blockade of GPR84 suppresses leukocyte infiltration in multiple murine inflammatory models.

## Abstract (web summary)

GPCRs of the GPR84 family are key regulators of leukocyte extravasation. *Drosophila* and murine in vivo models show that leukocyte GPR84/Tre-1 support dynamic Rho-dependent detachment of stretched uropods from vessels.

## Graphical abstract

**Figure:**
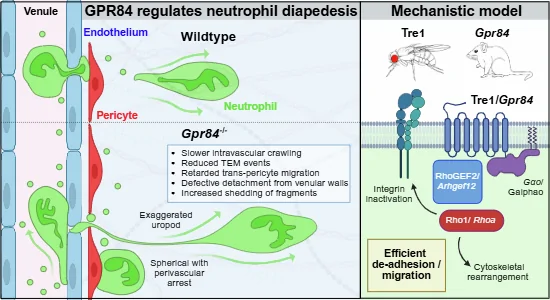

## Introduction

A key rate-limiting step in the development of an acute inflammatory reaction is the emigration of innate immune cells (neutrophils and monocytes) from the blood circulation to sites of tissue infection or damage (Nourshargh and Alon, 2014; Muller, 2016; Vestweber, 2015). This response begins with the capture and adhesion of leukocytes to venular walls, as described by the classical adhesion cascade, with the final step being the breaching of vessel walls (commonly post-capillary venules) (Ley et al, 2007). The latter, termed immune cell extravasation (also known as transmigration or diapedesis) (Nourshargh and Alon, 2014; Nourshargh et al, 2010), is a dynamic process that involves the active participation of both immune and vascular cells (Nourshargh and Alon, 2014; Muller, 2016; Vestweber, 2015).

Murine genetic approaches, combined with high-resolution intravital microscopy and complementary in vitro flow studies, have laid out the key steps and molecular players that underpin the initiation and progression of leukocyte extravasation (Muller, 2016; Vestweber, 2015; Nourshargh and Alon, 2014; Ley et al, 2007; Phillipson et al, 2006). To exit the blood circulation, immune cells are required to breach different components of the venular wall, namely endothelial cells (ECs) that line the inner aspect of all blood vessels, as well as pericytes and the venular basement membrane (BM). Characterization of immune cell and EC/pericyte expressed adhesion molecules, most notably proteins expressed at EC junctions (such as PECAM-1, JAM-A, JAM-C, and VE-cadherin), and immobilized chemokines, has shed much light on the underlying molecular mechanisms (Vestweber, 2015; Nourshargh and Alon, 2014; Muller, 2016; Ley et al, 2007). In breaching the endothelial layer, leukocytes commonly migrate through EC junctions (paracellular), and less frequently in the peripheral circulation, they exhibit migration through the body of individual ECs (transcellular). Once in the sub-EC space, leukocytes probe for weak spots in the basement membrane and gaps in the pericyte sheath, as directed by chemotactic cues from vascular and perivascular cells before fully exiting the venular wall and commencing their extravascular migration (Joulia et al, 2022; Girbl et al, 2018; Proebstl et al, 2012).

Despite our in-depth understanding of this process, the signaling events that enable the successful completion of each sequential step of leukocyte extravasation remain unclear. Unraveling such dynamic molecular cascades in vivo at the single-cell level necessitates tractable models amenable to high-resolution live imaging and cell-specific genetic manipulation. Whilst *Drosophila* were not previously considered an appropriate model to investigate immune cell extravasation because of their open circulation (Razzell et al, 2011), we recently identified a period during their pupal development when the beating wing hearts pulse blood (including the hemocytes, *Drosophila* leukocytes) through the developing wing vessels (known as wing “veins”) (Thuma et al, 2018). Here, wounding of the adjacent epithelium triggers the recruitment of hemocytes across the vessel wall that can be imaged in real-time with high spatiotemporal resolution (Thuma et al, 2018). Capitalizing on this model and harnessing *Drosophila’s* unrivaled genetic tractability, we identified *Tre1* (*Trapped in endoderm 1*), a Rhodopsin family G protein-coupled receptor (GPCR), as essential for hemocyte extravasation across vessel walls. In addition, live imaging studies revealed a failure of Tre1-deficient hemocytes to fully extravasate, suggesting a non-redundant role for Tre1 in modulating immune cell extravasation machinery (Thuma et al, 2018).

In the present study, we have undertaken an in-depth characterization of the functions of GPR84, the closest mammalian ortholog of Tre1, in leukocyte extravasation in murine models of inflammation using both genetic and pharmacological strategies. GPR84, an orphan GPCR of the rhodopsin-like Class A family (Wittenberger et al, 2001), exhibits mRNA expression largely restricted to peripheral immune cells (predominantly myeloid cells and microglia in the CNS) and is upregulated in certain inflammatory settings (Bouchard et al, 2007; Recio et al, 2018; Yousefi et al, 2001). While GPR84 has been identified as a pro-inflammatory immune cell receptor in experimental models of inflammation (Recio et al, 2018; Cooper et al, 2024; Puengel et al, 2020; Abdel-Aziz et al, 2016; Gagnon et al, 2018; Wang et al, 2023), its precise role in mediating key steps in immune cell migration at the single-cell level is unknown.

To address this, we have integrated genetic, live imaging, transcriptomic, electron microscopy, and pharmacological studies to shed light on the subcellular mechanisms through which GPR84 orthologs exhibit conserved functions during leukocyte extravasation. Briefly, genetic knockout of murine GPR84 impaired all stages of neutrophil diapedesis, from breaching the endothelium and the pericyte layer through to final detachment from the venular wall. Strikingly, live imaging and correlative light electron microscopy (CLEM) revealed a key impairment in GPR84-deficient leukocytes during the final stages of extravasation, namely failure of the leukocyte uropod to detach from the vessel wall. Our mechanistic studies involving both *Drosophila* and murine models suggest that Tre1/GPR84 plays an essential role in promoting Rho GTPase-driven release of integrin-mediated adhesions. Consistent with this, pharmacological blockade of GPR84 signaling in vivo significantly dampened neutrophil recruitment in multiple acute inflammatory reactions, including experimental models of cutaneous ischemia–reperfusion injury, peritonitis, and skin wound healing. Collectively, our observations identify a distinct role for the conserved GPCR84 family as rate-limiting players in immune cell extravasation, with potential for therapeutic targeting for pathologies characterized by overexuberant inflammation.

## Results

### The GPCR Tre1 is required for effective hemocyte extravasation from *Drosophila* pupal veins

**Figure 1:**
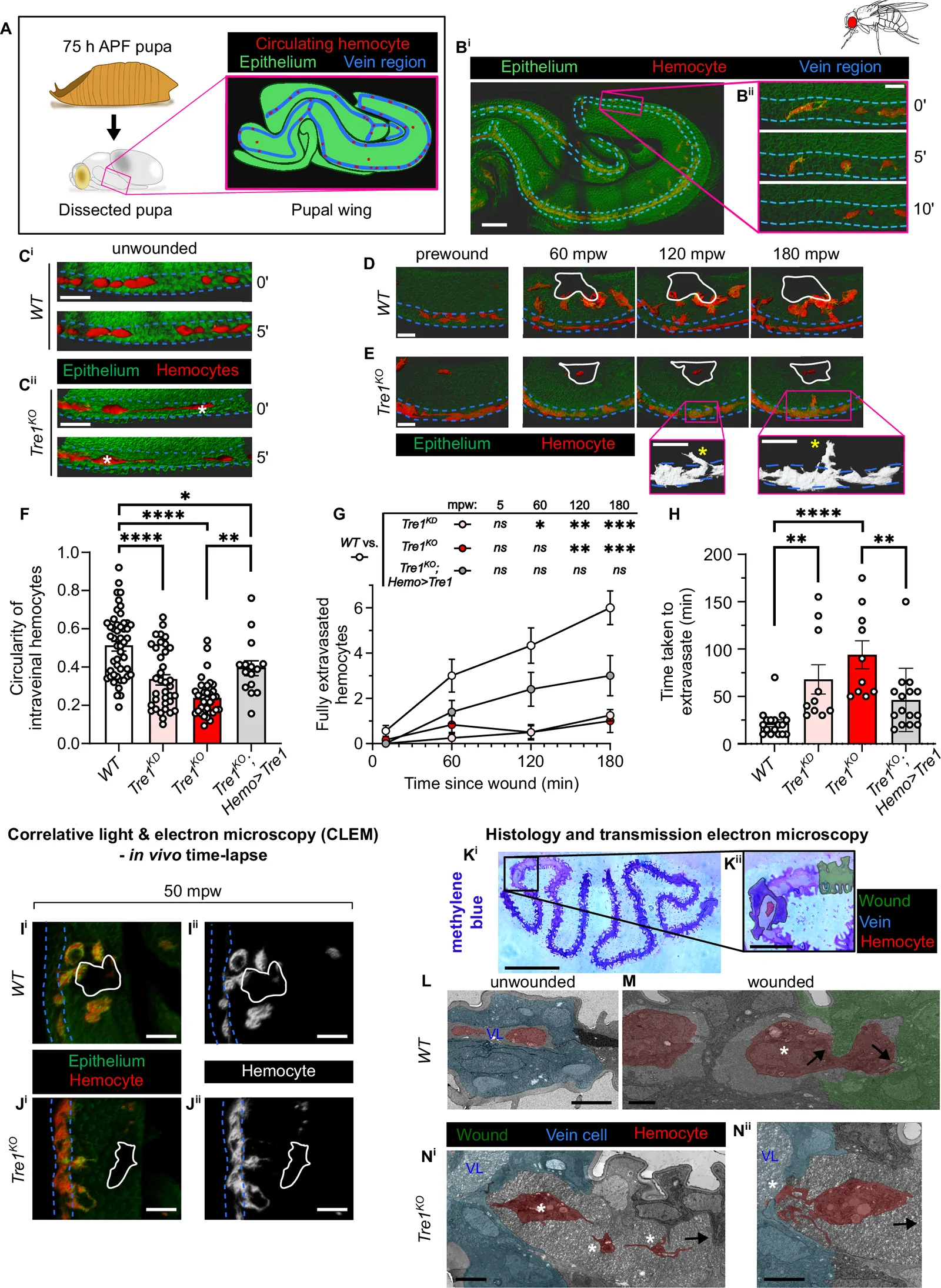
*Drosophila* late-pupal model of immune cell extravasation. (**A**) Schematic to illustrate how 75 h APF (after puparium formation) pupae are dissected to enable confocal imaging of the developing wings. (**B**^**i**^) Representative in vivo imaging of whole-pupal wing with epithelium labeled in green (*ubi-GMA*), hemocytes labeled in red (*srp-Gal4>mCherry; srp-mch*), and vein regions indicated with a blue dotted line. (**B**^**ii**^) Time-lapse IMARIS renderings of a vein region with circulating hemocytes. (**C**^**i**^,** C**^**ii**^) Comparison of *Oregon R* (*WT*) and *Tre1*^*KO*^ hemocytes’ morphology as they circulate in unwounded conditions. *Tre1*^*KO*^ hemocytes have cytoplasmic extensions that appear stuck to the luminal side of the vein cells (white asterisks). (**D**) Time-lapse confocal images of *WT* hemocytes (red) responding to a laser wound (solid white outline). (**E**) By contrast, *Tre1*^*KO*^ hemocytes cannot fully dissociate from the vessel wall. High magnification renderings (from regions in magenta boxes) illustrate the distinct morphology of mutant hemocytes and their long leading edges (yellow asterisks). (**F**) Comparison of the circularity of intravenous circulating *WT* hemocytes against *Tre1*^*KD*^ (*P* < 0.0001), *Tre1*^*KO*^ (*P* < 0.0001), or against hemocytes expressing Tre1 in a Tre1 null background (*P* = 0.0425); hemocytes expressing Tre1 in a Tre1 null background were more circular than *Tre1*^*KO*^ hemocytes (*P* = 0.0061). In total, 132 cells were analyzed, with cells sampled from 5 to 16 flies per genotype. Bar plots represent the calculated mean for each genotype, and dots represent individual hemocytes pooled from multiple animals. (**G**) Quantification of the number of hemocytes that have fully dissociated from veins and extravasated towards wounds at various timepoints. *WT* extravasation events were compared against *Tre1*^*KD*^ (60 mpw, *P* = 0.0144; 120 mpw, *P* = 0.0028; 180 mpw, *P* = 0.0004), *Tre1*^*KO*^ (120 mpw, *P* = 0.0027; 180 mpw, *P* = 0.0003), or against hemocytes expressing Tre1 in a Tre1 null background (all timepoints, *P* > 0.05). (**H**) Analysis of time taken for individual *WT* hemocytes to extravasate from veins versus *Tre1*^*KD*^ (*P* = 0.0099), *Tre1*^*KO*^ (*P* < 0.0001), or against hemocytes expressing Tre1 in a Tre1 null background (*P* > 0.05); the time taken for *Tre1*^*KO*^ hemocytes to extravasate was significantly slower than that of hemocytes expressing Tre1 in a Tre1 null background (*P* = 0.0093). Bar plots represent the calculated mean for each genotype, and dots represent individual hemocytes pooled from 4 to 9 animals per genotype. (**I**, **J**) In vivo confocal imaging of *WT* (**I**,** I**^**i**^) versus *Tre1*^*KO*^ (**J**,** J**^**i**^) hemocytes as they extravasate towards wounds (white outline), just prior to fixation at 50 min post wounding (mpw) for further EM analysis in a correlative light and electron microscopic (CLEM) study. (**K**^**i**^) Methylene blue-stained resin-embedded transverse sections of a *WT* wounded sample. (**K**^**ii**^) Higher magnification view (from black boxed region) reveals a single hemocyte (false-colored red) within the lumen of the vein (blue) with a wound (green) adjacent to the vessel. (**L**) Transmission electron microscopy (TEM) of an unwounded *WT* vein (blue) with two hemocytes (red) within. (**M**) *WT* hemocytes (red) 15 min *post* extravasation as they traverse the intervein epithelium towards a wound (green) with a hemocyte already at the wound (white asterisk). (**N**^**i**^, **N**^**ii**^) *Tre1*^*KO*^ hemocytes have partially extravasated and exhibit extensive leading edges, but remain adherent to the vein cells, with multiple lagging tails observed (white asterisks). (**L**–**N**) VL denotes the position of the vein lumen; in (**M**, **N**) black arrows indicate the position or direction of the wound, respectively. Data in plots (**F**, **H**) were analyzed using one-way ANOVA with a Dunnett’s test performed for multiple comparisons. Data in (**G**) were analyzed using a mixed-effects model with a Dunnett’s test performed for multiple comparisons. For each quantification, data represent at least nine animals per genotype and represent the mean ± SEM. *P < 0.05, **P < 0.01, ***P < 0.001, ****P < 0.0001. Scale bars: (**B**, **K**^**i**^) = 50 µm; (**B**^**i**^, **C**^**i**^, **C**^**ii**^, **D**, **E**, **I**^**i**^, **J**^**ii**^, **K**^**ii**^) = 15 µm; (**L**) = 5 µm; (**M**, **N**^**i**^, **N**^**ii**^) = 2 µm. See also Movie EV1. Source data are available online for this figure.

We recently demonstrated that *Drosophila* pupal wings, in which hemocytes circulate through vessels (the wing “veins”), offer the opportunity to model immune cell extravasation in response to an inflammatory stimulus in the form of a small epithelial wound adjacent to a wing vein (Fig. 1A–D) (Thuma et al, 2018). Within this model, we can observe hemocytes circulating within the hemolymph pulsing through wing vessels, with vessel LV3 (that extends in one focal plane) being particularly amenable for imaging (Fig. 1B,C). Within 10 min of making a laser wound in the epithelium adjacent to this vessel, hemocytes are seen to probe the vessel wall in the vicinity of the injury before extravasating and subsequently migrating towards the wound (Fig. 1D).

**Figure EV1:**
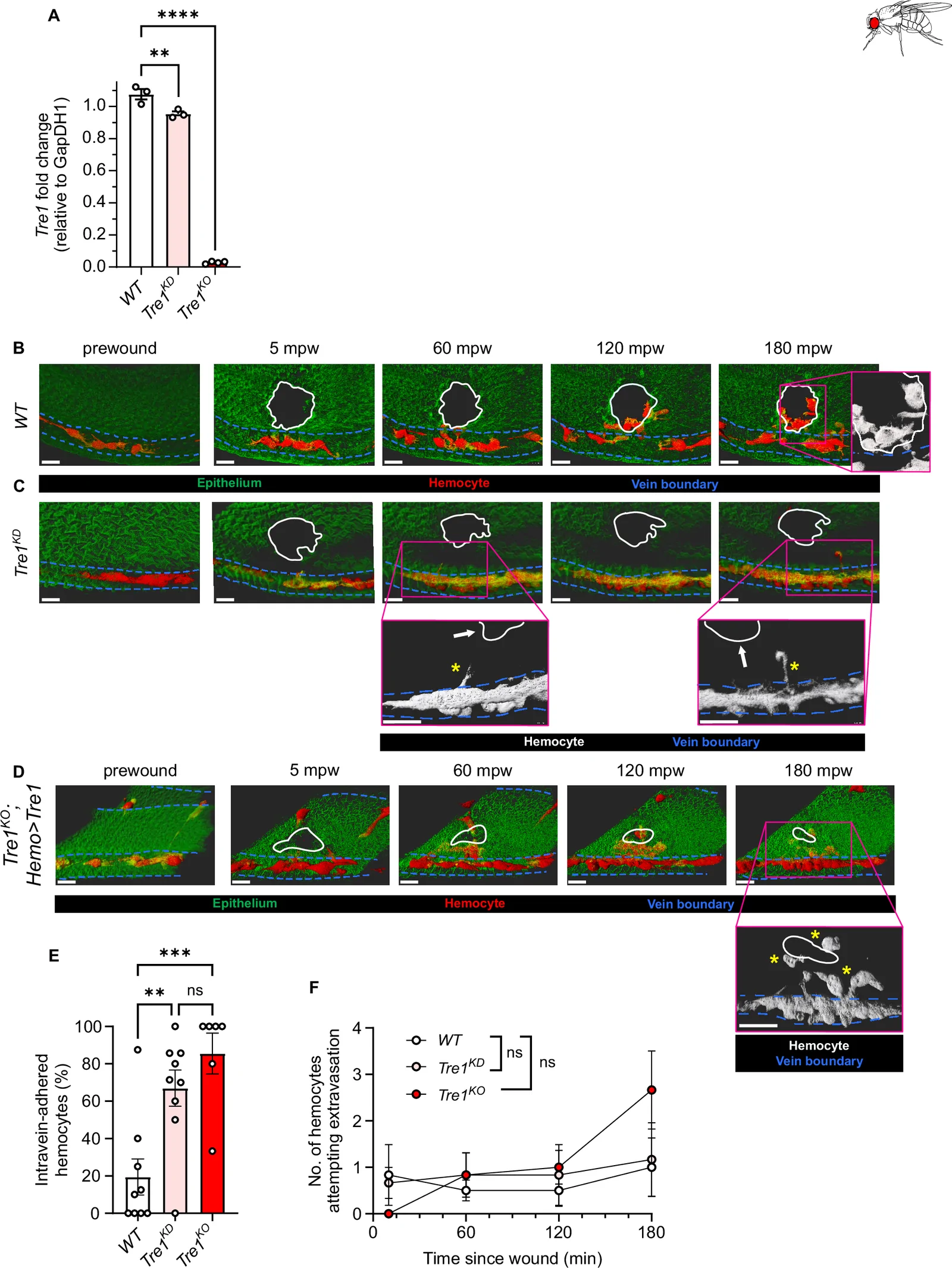
Tre1 is required by hemocytes to complete extravasation. (**A**) Quantitative RT-PCR of the relative expression of *Tre1* in 75 h APF whole-pupae in *Oregon R* (*WT*) background versus pupae with RNAi-mediated silencing of *Tre1* (*Tre1*^*KD*^) in hemocytes (*srp-Gal4*-driven knockdown) or in fully *Tre1* null pupae (*P* = 0.0051 and *P* < 0.0001, respectively). *Tre1* threshold cycle values were normalized to *Gapdh1*. RT-PCR data represented in a bar plot (mean ± SEM) were overlaid with average values (white dots) for each biological replicate (RNA pooled from 10 animals). Data was analyzed by one-way ANOVA with a Dunnett’s test for multiple comparisons. (**B**, **C**) Representative in vivo time-lapse imaging of 75 h APF with *Tre1*^*KD*^ hemocytes, which results in a significant reduction in hemocyte extravasation capacity as compared to *WT*. (**D**) Representative in vivo time-lapse imaging of 75 h APF *Tre1* null pupae expressing *Tre1* specifically in hemocytes (*srp*-*Gal4*-driven expression), which partially recovered the Tre1 extravasation phenotype. Solid white line indicates the border of the wound, white arrows indicate the direction towards the wound that hemocytes extravasated towards, and asterisks indicate hemocytes that have either fully or partially extravasated. (**E**) Comparison of the proportion of intraveinally adhered hemocytes between *WT* and *Tre1*^*KD*^ (*P* = 0.0054) or *Tre1*^*KO*^ (*P* = 0.0007) genotypes. Hemocytes were classified as adhered if they remained in the same location between consecutive frames (remained still for five minutes). Between six and nine animals were analyzed for each genotype, and data represent mean ± SEM. Data were analyzed by one-way ANOVA with a Tukey’s test for multiple comparisons between genotypes. (**F**) Quantification of the number of hemocytes attempting to extravasate at various timepoints after wounding. Six animals were analyzed for each genotype, and data represent mean ± SEM. A two-way ANOVA was performed followed by Tukey’s multiple comparisons which determined no significant differences between genotypes at any time point. *P < 0.05, **P < 0.01, ***P < 0.001, ****P < 0.0001. (**C**, **D**) Yellow asterisks indicate hemocytes, and white arrows show the direction of travel towards wounds. Scale bars: (**B**–**D**) = 15 µm.

Harnessing the live imaging opportunities of the translucent pupa, together with *Drosophila’s* genetic tractability, we sought novel genes that might drive immune cell extravasation. Here, extending our previous works (Thuma et al, 2018), and utilizing both a hemocyte-specific *Tre1-RNAi* knockdown (*Tre1*^KD^) approach and a Tre1 null (*Tre1*^KO^) mutant (in which the endogenous Tre1 locus has been removed) (Kim et al, 2021), our live imaging studies show Tre1 to be fundamental for complete hemocyte extravasation (Figs. 1C–H and  EV1A–C). An additional genetic recovery approach in which we re-express Tre1 specifically in hemocytes (using *UAS-Tre1* driven by *srp-Gal4*) (Chen et al, 2024) within a *Tre1* null background confirmed a role for hemocyte Tre1 during extravasation (Figs. 1G,H and  EV1D). Intriguingly, within the wing veins, hemocytes lacking Tre1 showed an intravenous morphological phenotype even in unwounded conditions; Tre1-deficient hemocytes exhibited cytoplasmic extensions that appeared stuck to the luminal side of the vein cells, resulting in reduced circularity and luminal adherence (Figs. 1C,F and  EV1E), a sustained phenomenon that was evident for the duration of the imaging period. Given that hemocyte-specific Tre1 expression recovered the circularity and extravasation defects of the *Tre1* null, our data suggest an autonomous role for Tre1 within hemocytes.

Following wounding, *Tre1* null hemocytes showed an extreme phenotype, with fewer hemocytes fully extravasating and those that did fully extravasate, taking longer than that of controls or RNAi-based Tre1 knockdowns (Figs. 1D–H and EV1B–D; Movie EV1). These striking extravasation defects occurred despite no initial differences in hemocyte recruitment (Fig. EV1F). The tails of Tre1-deficient hemocytes remained adherent to the vein walls, suggesting that Tre1 may regulate the perivascular detachment phase of hemocyte extravasation (Fig. 1E). We next undertook correlative light and transmission electron microscope imaging (CLEM) studies to compare hemocyte morphologies post wounding in control and Tre1-deficient pupae (Fig. 1I–N). Time-lapse movies enabled the capture of hemocytes in the act of extravasating in vivo (Fig. 1I,J), and these samples were immediately prepared to visualize this process at the ultrastructural level via transmission electron microscopy (Fig. 1K–N). In control unwounded pupae, we observed hemocytes within the lumen of the wing vein (Fig. 1L); following wounding, hemocytes were observed outside of the vessel exhibiting relatively short lamellipodia en route to the wound (Fig. 1M). Tre1-deficient hemocytes, however, showed long leading extensions and retained multiple lagging tethers linking them to the vein wall cells and to the interior of the vein (Fig. 1N^i^,N^ii^). Consistent with our live-imaging studies, these CLEM data suggest that hemocytes are disrupted in their capacity to extravasate completely through wing vessels after a sterile injury, potentially due to an incapacity to de-adhere from vessel cells.

### Tre1 shares structural homology with its conserved mammalian ortholog, GPR84

**Figure 2:**
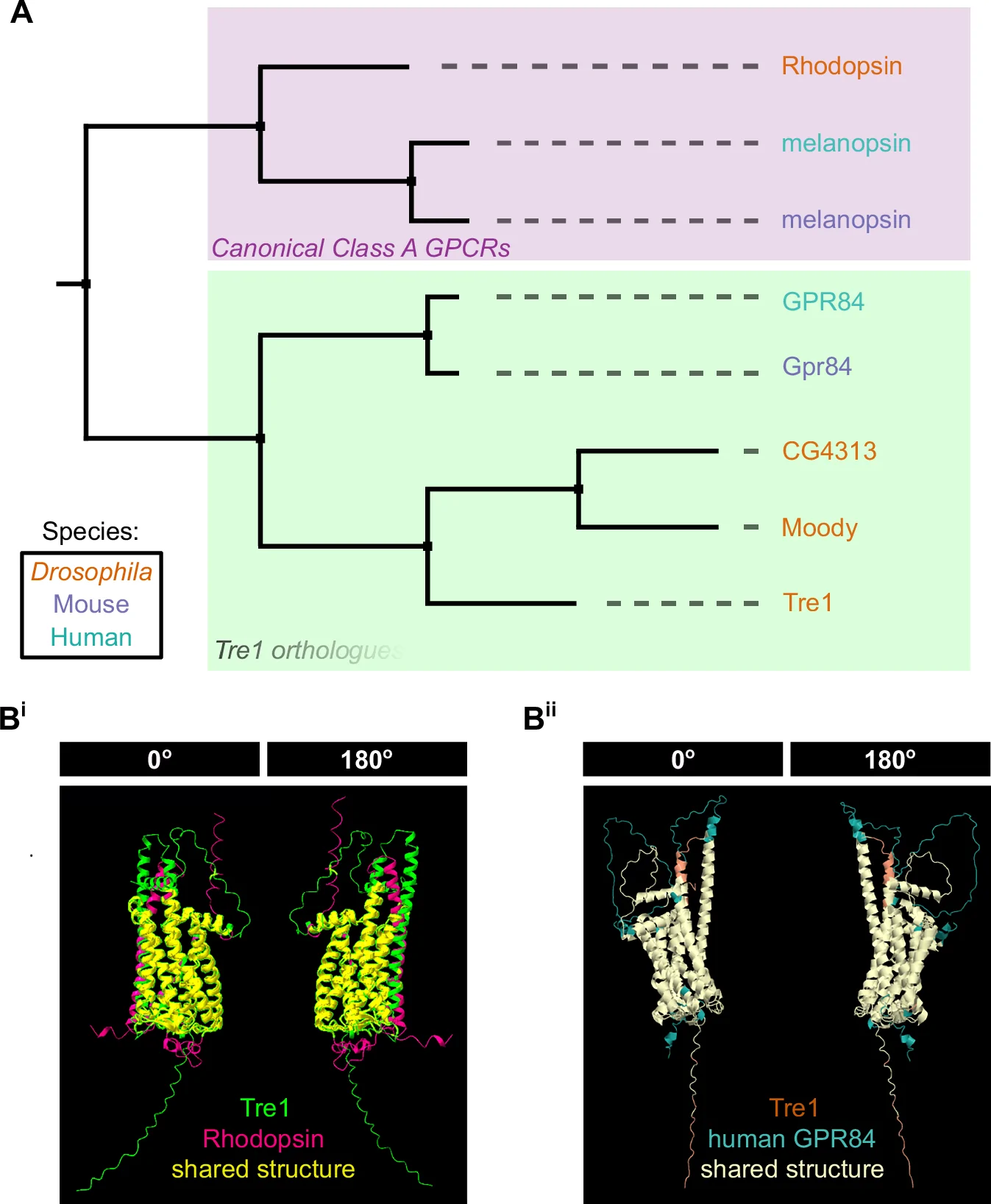
Tre1 is related to and shares structural homology with its mammalian ortholog GPR84. (**A**) Phylogenetic tree constructed from multiple sequence alignment between predicted Tre1 paralogues in *Drosophila* (maroon), and orthologs in mice (blue) and humans (teal). The canonical Class A GPCR NinaR (AKA, Rhodopsin) in *Drosophila* and its mammalian orthologs were added as a non-homologous routing comparison to Tre1. The size of horizontal branches corresponds to the estimated level of evolutionary conservation between sequences on adjacent nodes. (**B**^**i**^) Structural comparison of AlphaFold3 predicted models (two orientations: back and front) for *Drosophila* Tre1 (maroon) and Rhodopsin (teal), with shared homologous structural regions indicated (yellow). The non-homologous sequence alignment between *Drosophila* Rhodopsin and Tre1 yielded a sequence alignment score of 512.1, where 153 Cα pruned pairs had a RMSD of 1.127 Å and across all 358 Cα pairs the RMSD was 13.97 Å. (**B**^**ii**^) Structural comparison of AlphaFold3 predicted models (two orientations: back and front) for Tre1 (maroon) and human GPR84 (teal), with their extensively shared homologous structural regions indicated (yellow). Orthologs alignment yielded 238 Cα pairs with a RMSD of 1.14 Å (1.135 Å, pruned), and across all 314 aligned pairs, the RMSD was 4.10 Å. Protein structures were aligned with UCSF ChimeraX Matchmaker (Pettersen et al, 2021). See also Movie EV2. Source data are available online for this figure.

To assess whether our *Drosophila* model could identify extravasation players with conserved roles in mammals, we explored whether the human and mouse genomes possess Tre1 orthologs. Phylogenetic analysis of amino acid sequences revealed several *Drosophila* paralogues and mammalian orthologs of Tre1 (Fig. 2A), including GPR84 with 34% amino acid sequence homology. Neither Tre1 nor its mammalian orthologs have experimentally derived structures, but AlphaFold enabled us to compare their predicted structures (Fig. 2B; Movie EV2) (Jumper et al, 2021). This in silico comparison suggested high structural homology between Tre1 and human GPR84 with significant identical elements (yellow, Fig. 2B^ii^), suggesting likely shared functionality; less structural homology was observed between Tre1 and an unrelated Class A GPCR, *Drosophila* Rhodopsin (yellow, Fig. 2B^i^).

### GPR84 mediates neutrophil extravasation in a murine model of acute inflammation

**Figure 3:**
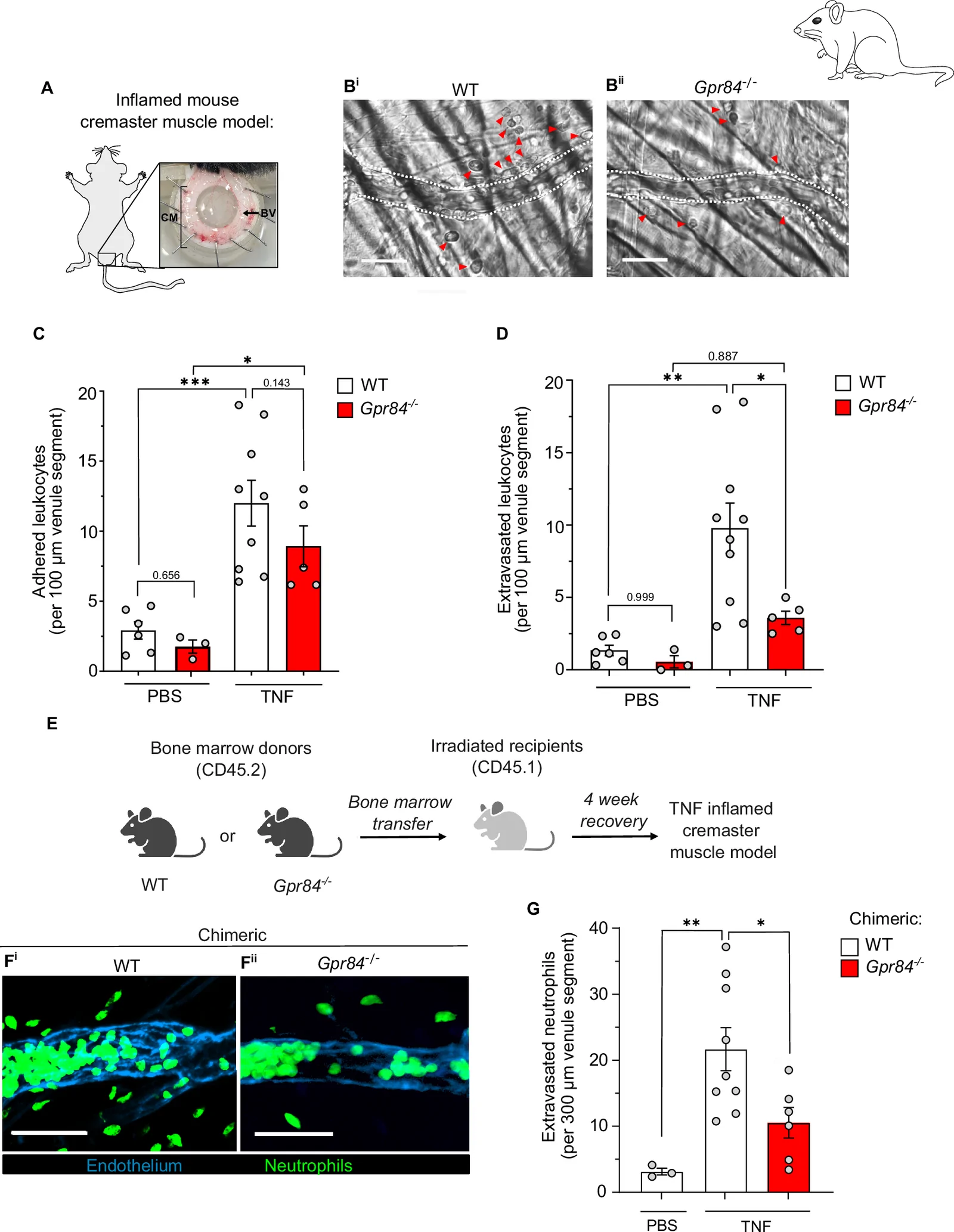
Myeloid cell GPR84-deficiency reduces neutrophil extravasation in TNF-stimulated cremaster muscle. (**A**) Schematic shows the exteriorized murine cremaster muscle (CM), subjected to TNF-induced inflammation immediately prior to intravital microscopy analysis. The arrow indicates the cremasteric microvasculature and the presence of blood vessels (BV). (**B**) Representative images of (**B**^**i**^) WT and (**B**^**ii**^) *Gpr84*^*−/−*^ cremasteric post capillary venules subjected to intrascrotal injection of TNF and subsequently imaged by brightfield intravital microscopy (IVM) at 4 h. Arrows indicate the presence of extravasated leukocytes within the interstitial tissue. Number of firmly adhered (**C**) and extravasated (**D**) leukocytes in WT and *Gpr84*^*−/−*^ tissues stimulated with PBS or TNF (*n* = 3–10 mice/group); each data point represents the average number of leukocytes from 2 to 8 venules per mouse. (**C**) Data compares PBS and TNF treatments in WT (*P* < 0.0001) and *Gpr84*^*−/−*^ mice (*P* = 0.0136). (**D**) Data compares PBS and TNF treatments in WT mice (*P* = 0.0018) and TNF-stimulated WT and *Gpr84*^*−/−*^ mice (*P* = 0.0335). (**E**) Schematic depicting the generation of WT and *Gpr84*^−/−^ bone-marrow chimeric mice. (**F**) Inflamed cremaster muscles were fixed and immunostained for endothelial cells (PECAM-1; blue) and neutrophils (MRP-14; green). Representative confocal images of cremasteric postcapillary venules from (**F**^**i**^) WT and (**F**^**ii**^) *Gpr84*^*−/−*^ chimeric mice. (**G**) Quantification of the average number of extravasated neutrophils from 6 to 13 venule segments in TNF-inflamed cremaster muscles of chimeric WT and *Gpr84*^−/−^ mice (*n* = 3–9 mice/group). Data shows statistical significance between PBS-treated and TNF-stimulated WT mice (*P* = 0.0081) and between WT and *Gpr84*^*−/−*^ TNF-stimulated tissues (*P* = 0.0428). Each data point represents an individual mouse, and the data shows mean ± SEM. (**C**, **D**) were analyzed by two-way ANOVA with uncorrected Fisher’s LSD or Šídák’s multiple comparisons test. (**G**) was analyzed by one-way ANOVA with a Tukey’s test for multiple comparisons. *P < 0.05, **P < 0.01. Scale bars: (**B**) = 20 µm, (**F**) = 50 µm. See also Movie EV3. Source data are available online for this figure.

**Figure EV2:**
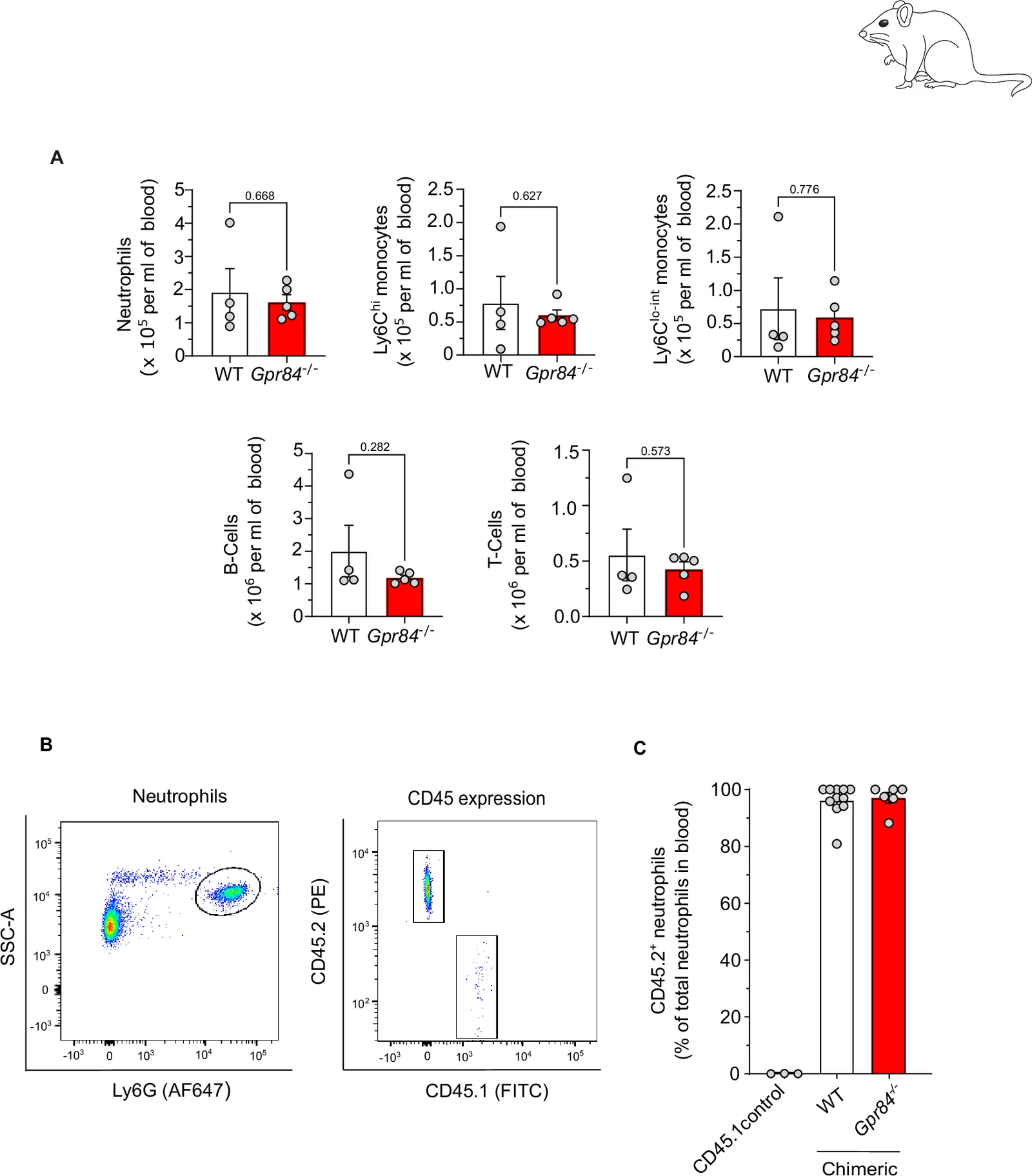
Blood leukocyte counts of WT vs *Gpr84*^*−/−*^ global knockouts and reconstitution efficiency in chimeric mice after bone marrow transfer. (**A**) Differential leukocyte counts of whole blood from WT (*n* = 4) and *Gpr84*^*−/−*^ (*n* = 6) mice determined by flow cytometry. (**B**) Representative flow cytometry gating strategy for selecting neutrophils, and (**C**) assessing CD45.2 allelic variant expression as a marker of reconstitution efficiency when generating WT and *Gpr84*^*−/−*^ chimeric mice. Data represent mean ± SEM and in (**A**) were analyzed by an unpaired *T* test with no significance between experimental groups.

Extending the *Drosophila* works, we sought to investigate the functional role of the predicted mammalian ortholog of Tre1, GPR84. In initial studies, aiming to unravel the stage of immune cell migration mediated by GPR84, we analyzed immune cell behavior of WT and *Gpr84*^*−/−*^ mice in control (PBS) or acutely- inflamed mouse cremaster muscles by brightfield intravital microscopy (IVM) (Fig. 3A–D; Movie EV3). Here, local TNF (4 h) induced a significant and comparable level of intraluminal leukocyte adhesion in both WT and *Gpr84*^*−/−*^ mice, as compared to PBS-treated tissues (Fig. 3C). In contrast, TNF-induced leukocyte extravasation was significantly reduced in *Gpr84*^*−/−*^ mice as compared to WT littermates (Fig. 3D). Furthermore, chimeric animals with selective myeloid GPR84 deficiency (generated through transfer of bone marrow cells to irradiated recipients; Fig. 3E), showed defective neutrophil extravasation (Fig. 3F,G). Of note, we detected no differences in leukocyte adhesion or extravasation, nor any distinct morphological differences in PBS-treated WT or KO tissues. Furthermore, we saw no difference in the total or differential leukocyte counts in the circulating blood of WT and KO mice under basal conditions Fig. EV2A), and the chimeric mouse lines showed equal reconstitution efficiency (Fig. EV2B,C). Collectively, the present findings illustrate a specific and cell-autonomous role for GPR84 in regulating the passage of leukocytes through inflamed venular walls.

### GPR84-deficient neutrophils display excessive adhesive interactions within inflamed vessels

**Figure 4:**
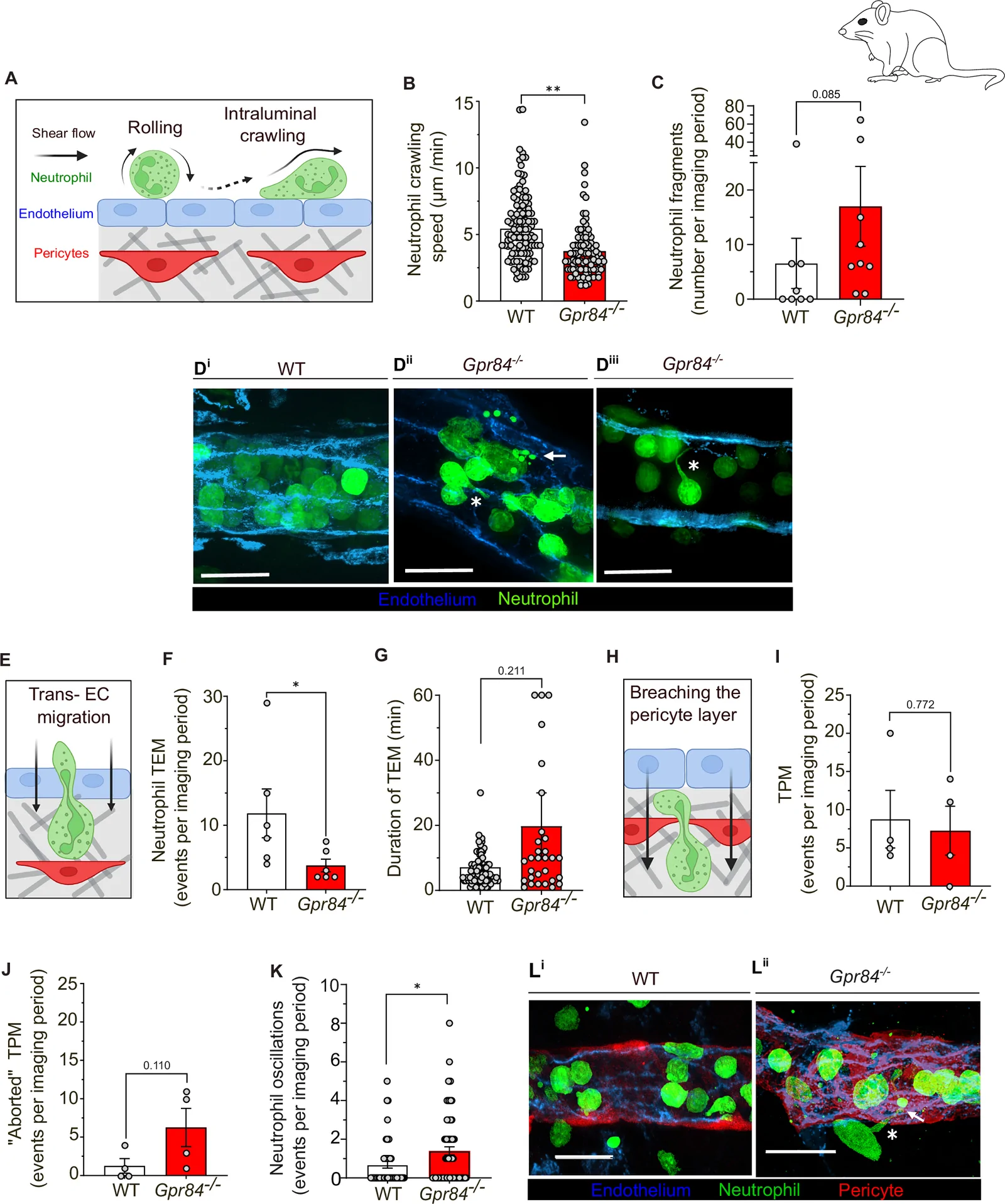
GPR84-deficient neutrophils display impaired transendothelial migration (TEM) and aberrant breaching of the pericyte layer during extravasation. (**A**–**J**) The cremaster microvasculature of WT and *Gpr84*^*−/−*^ mice on a *Lyz2-EGFP-ki; Acta2-RFPcherry-Tg* genetic background was acutely inflamed with intrascrotal injection of TNF (300 ng) and subsequent extravasation events recorded by confocal IVM. (**A**) Schematic depicting the response of intraluminal neutrophil crawling analyzed in (**B**). (**B**) Quantification of intraluminal neutrophil crawling velocity with each data point representing an individual neutrophil from 1 to 2 venules per mouse; WT (116 cells, *n* = 5 mice) and *Gpr84*^*−/−*^ (99 cells, *n* = 6 mice) (*P* = 0.0067) and (**C**) number of neutrophil-derived fragments from 1 to 2 venules in WT (*n* = 8) and KO (*n* = 9) mice. (**D**) Confocal images of luminal neutrophils in venules of WT (**D**^**i**^) and *Gpr84*^*−/−*^ mice (**D**^**ii**^, **D**^**iii**^) 4 h post administration of TNF. (**D**^**ii**^, **D**^**iii**^) Arrows show increased deposition of neutrophil fragments in *Gpr84*^*−/−*^ mice, and asterisks indicate the presence of elongated neutrophil uropods tethered to endothelial cells. (**E**) Schematic depicting the response being analyzed in (**F**, **G**), i.e., transendothelial cell migration (TEM). (**F**, **G**) Quantification of (**F**) the number of neutrophil TEM events within an imaging period of 2–5 h with each data point representing the average from 1 to 2 venules per mouse (*P *= 0.0238), and (**G**) duration of neutrophil TEM (in min); WT (86 cells) and *Gpr84*^*−/−*^ (30 cells) *n* = 6 mice/group. Bar plots represent the calculated mean value for each genotype, and data points represent single neutrophils pooled from multiple animals. (**H**) Schematic depicting the response being analyzed in (**I**–**K**), i.e., trans-pericyte migration (TPM). (**I**–**K**) Number of neutrophils exhibiting TPM events (**I**, **J**) the number of “aborted” TPM events, presented as the average from 1 to 2 venules per mouse within an imaging period, WT (*n* = 4) and *Gpr84*^*−/−*^ (*n* = 4). (**K**) Number of neutrophil oscillations at the pericyte layer; each data point represents an individual neutrophil pooled from 1 to 2 venules per mouse, WT (56 cells, *n* = 4 mice) and *Gpr84*^*−/−*^ (69 cells, *n* = 4 mice), showing a statistically significant difference between WT and KO cells (*P* = 0.0133). (**L**) Confocal images of neutrophil morphology when breaching the pericyte layer in (**L**^**i**^) WT and (**L**^**ii**^) *Gpr84*^*−/−*^ mice. Asterisk indicates sub-endothelial neutrophil structures with (**L**^**ii**^) displaying an elongated tail retained within the sub-EC space and increased deposition of neutrophil fragmented structures (arrow). Data shows mean ± SEM. Data were analyzed by unpaired *T* test or Mann–Whitney *U* test (on the means per mouse or in (**K**) between pooled neutrophils) with significance shown by *P < 0.05, **P < 0.01. Scale bars: (**B**, **J**) = 20 µm. (**A**, **C**, **F**) were created in BioRender. L, C (2025) https://BioRender.com/bup6fba. Source data are available online for this figure.

Having identified defective immune cell extravasation in both Tre1-deficient *Drosophila* and GPR84-deficient mice, we next sought to gain more insight into the mechanism of action of GPR84 in real-time and at the single-cell level using high-resolution 4D confocal IVM. For this purpose, we interbred *Gpr84*^*−/−*^ mice with the compound mouse reporter strain *Lyz2-EGFP-ki*; *Acta2-RFPcherry-Tg* that exhibits endogenous GFP^high^ neutrophils and RFP^+^ pericytes/smooth muscle cells. For imaging ECs, or more specifically EC junctions, the mice received a local injection of non-blocking fluorescently labeled anti-PECAM-1 mAb, as previously described (Woodfin et al, 2011). The application of this platform to exteriorized TNF-stimulated cremaster muscles revealed aberrant neutrophil behaviors in GPR84 KO mice at multiple stages of neutrophil extravasation, all reminiscent of defective de-adhesion. Quantification of luminal responses (Fig. 4A) revealed significantly slower crawling of KO neutrophils within inflamed postcapillary venules, as compared to the motility of WT neutrophils (Fig. 4B). This was associated with the detection of an increased number of neutrophil fragments in close apposition to sites of neutrophil adhesion in KO mice, as compared to WTs (Fig.  4C,D^i^–D^ii^). This response may allude to excessive neutrophil adhesion and difficulties with detachment from the endothelium, as further indicated by excessive tethering of *Gpr84*^*−/−*^ neutrophils to the endothelium (Fig. 4D^iii^). With respect to the latter, despite no significant difference in the absolute number of neutrophil tethers detected between WT and KO mice (0.186 ± 0.057 and 0.166 ± 0.047 tethers/intraluminal neutrophils over 1 h imaging period, respectively), the profiles detected between the two mouse lines were different. Specifically, 35% of WT neutrophils, as compared to 43% of *Gpr84*^*−/−*^ neutrophils, displayed tethers that were elongated by at least one cell body (≥ ~10 µm) in length. These observations are similar to the aberrant intraluminal structures detected in Tre1 null *Drosophila* hemocytes (Fig. 1C,F).

In analyzing the frequency and dynamics of neutrophil transendothelial cell migration (TEM) (Fig. 4E**)**, GPR84-deficient neutrophils displayed a significantly reduced number of TEM events (Fig. 4F) and consistently showed an extended duration to fully breach the endothelium (Fig. 4G). Post breaching of the endothelium, KO mice subsequently presented multiple aberrant interactions between neutrophils and pericytes (Fig. 4H). Specifically, despite no overall differences in the number of neutrophils breaching the pericyte layer (termed trans-pericyte migration; TPM) in WT and KO mice (Fig. 4I), we noted an increase in the number of neutrophils that probed but ultimately “aborted” their migration across the pericyte layer (Fig. 4J). Furthermore, KO neutrophils exhibited a distinct hesitancy and increased oscillatory behavior, characterized by a repetitive probing and retracting of the neutrophil cell body, prior to eventual completion of their migration across the pericyte sheath (Fig. 4K). In many instances, this wavering neutrophil-pericyte interaction was observed throughout the whole duration of the imaging period in GPR84 KO mice, and this step ultimately failed (see Movie EV4). Interestingly, similar to the defective intraluminal responses, post breaching the pericyte layer, a subset of *Gpr84*^*−/−*^ neutrophils again presented elongated tail-like structures within the sub-EC space (Fig. 4L). The latter were commonly associated with evidence of neutrophil fragment deposition, supporting the notion of a generally impaired leukocyte de-adhesion within inflamed vessel walls of KO mice.

### GPR84-deficient neutrophils exhibit defective detachment from inflamed vessels

**Figure 5:**
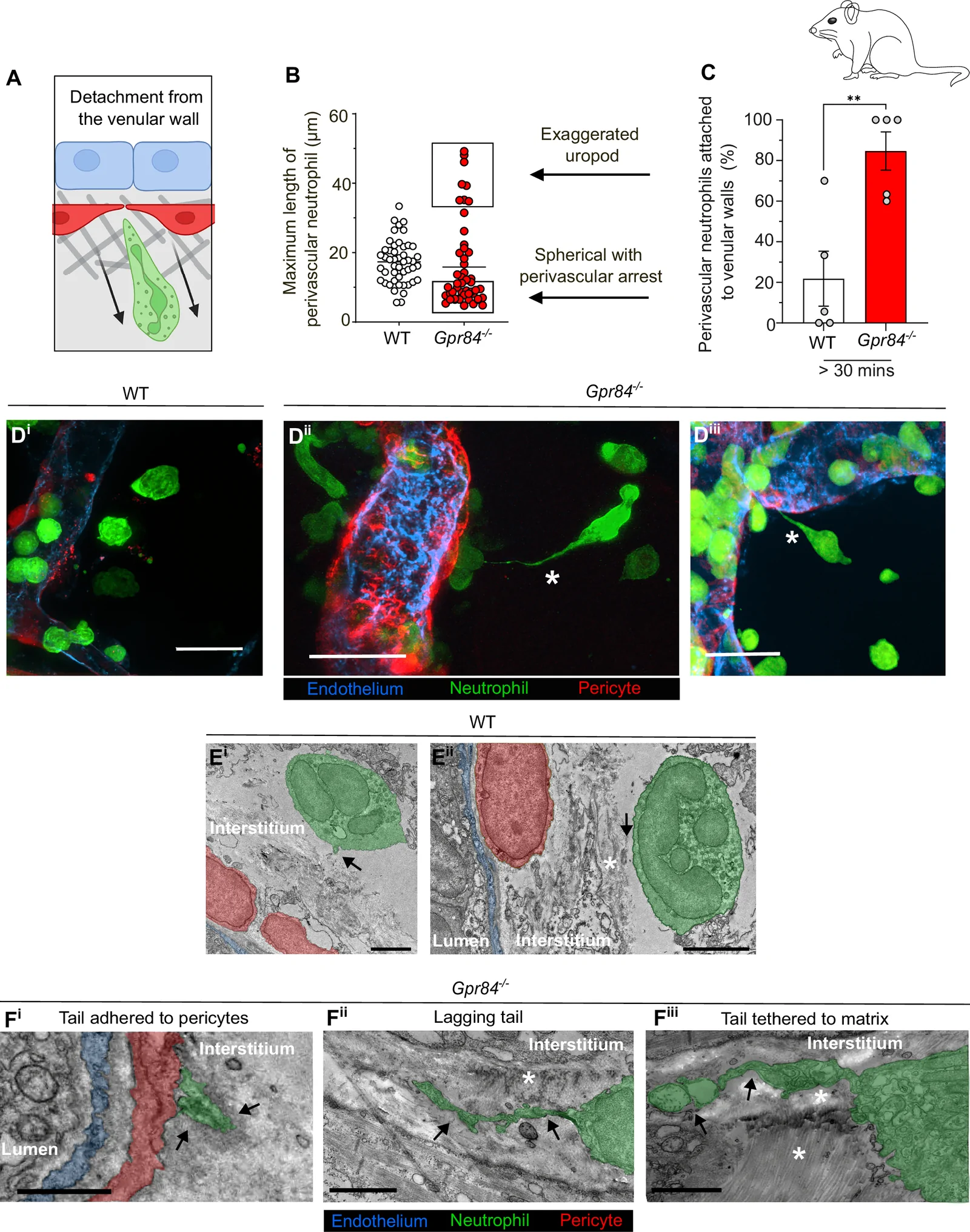
Neutrophil detachment from inflamed venular walls is impaired in GPR84-deficient mice. (**A**) Schematic depicting the response being analyzed in (**B**, **C**), i.e., neutrophil detachment from the venular wall after breaching the pericyte layer. (**B**) The maximum length of an extravasated neutrophil whilst still tethered to the vascular wall (defined as perivascular neutrophil) during a 1 h recording of a TNF-stimulated post-capillary venule. Each data point represents an individual neutrophil pooled from WT and *Gpr84*^*−/−*^ mice (*n* = 5 mice/group). For the *Gpr84*^*−/−*^ neutrophils, arrows indicate either an “exaggerated uropod” with an undulating motion prior to detachment or a “spherical” morphology with perivascular arrest. (**C**) Quantification of the percentage of perivascular neutrophils attached to the venular wall for >30 min during the 1 h imaging period (*P* = 0.0064). (**D**) Representative images of neutrophils extravasating in WT (**D**^**i**^) and *Gpr84*^*−/−*^ (**D**^**ii**^, **D**^**iii**^) cremaster muscles. Cells were identified as follows: Neutrophils (*Lyz2-EGFP*; green), endothelial cells (anti-PECAM-1 mAb; blue), and pericytes (*Acta2-RFPcherry*; red). Asterisks indicate *Gpr84*^*−/−*^ mice displaying a protracted attachment to the venular wall at the 4 h point of the reaction. (**E**, **F**) Transmission electron microscopy analysis of inflamed cremaster muscles showing the ultrastructure of neutrophils as they detach from the venular wall post-extravasation in WT (**E**) and *Gpr84*^−/−^ (**F**) mice. (**E**^**i**^, **E**^**ii**^) Representative images of WT neutrophils just after detaching from the venular wall at the experimental endpoint of 4 h. Black arrows indicate the remaining neutrophil (green) uropod post-detachment from the pericyte layer (red) and endothelium (blue). A white asterisk indicates the presence of extracellular matrix components in the interstitial space. (**F**) Representative images of protracted neutrophil attachments to venular walls in *Gpr84*^−/−^ mice, (**F**^**i**^) displaying retained pericyte attachment, (**F**^**ii**^) with a lagging tail, as indicated by arrows, and (**F**^**iii**^) tail retention along the extracellular matrix. Data in (**C**) represents mean ± SEM and analyzed by an unpaired *T* test (*n* = 5 mice/group). **P < 0.01. Scale bars: (**D**) = 20 µm; (**E**^**i**^) = 2 µm; (**E**^**ii**^) = 3 µm; (**F**) = 1.5 µm. Source data are available online for this figure.

Guided by the striking phenotype of elongated tails observed in Tre1-deficient hemocytes (Fig. 1), we extended our murine confocal IVM studies to analyze neutrophil detachment from inflamed venules in the perivascular space (Fig. 5A). As a quantitative assessment of neutrophil morphology, we measured the maximum length of perivascular neutrophils during the imaging period (Fig. 5B). This analysis revealed that in GPR84-deficient mice, perivascular neutrophils present two principal profiles, (i) a population exhibiting a “spherical” morphology that shows almost total perivascular arrest, and (ii) a population with an extended and persistent uropod formation, reaching >50 µm in length in some cells. The resultant effect was a delayed detachment of KO neutrophils from inflamed venular walls. Hence, in entering the perivascular space, while WT neutrophils displayed brief attachments (**∼**12 min) to the exit site of the venular wall, in *Gpr84*^*−/−*^ mice, **∼**80% of the perivascular neutrophils required >30 min to fully detach and enter the surrounding interstitial space (Fig. 5C; Movie EV5). The aberrant GPR84 KO neutrophil tails (Fig. 5D) were probed further at the ultrastructural level by transmission electron microscopy (Fig. 5E,F). Whereas, in WT mice we saw fully detached neutrophils with short uropods (as if recently detached from adjacent pericytes) (Fig. 5E), in *Gpr84*^*−/−*^ mice we observed extensive lagging tails that appeared tethered to the pericyte wall (Fig. 5F^i^). The latter were often associated with fibrillar matrix (Fig. 5F^ii^–F^ii^). Collectively, the findings suggest exaggerated and prolonged leukocyte adhesion at multiple sites within and along the venular wall.

### Transcriptomic analysis of GPR84-deficient murine neutrophils indicates defective actin cytoskeletal regulation and cell adhesion

**Figure 6:**
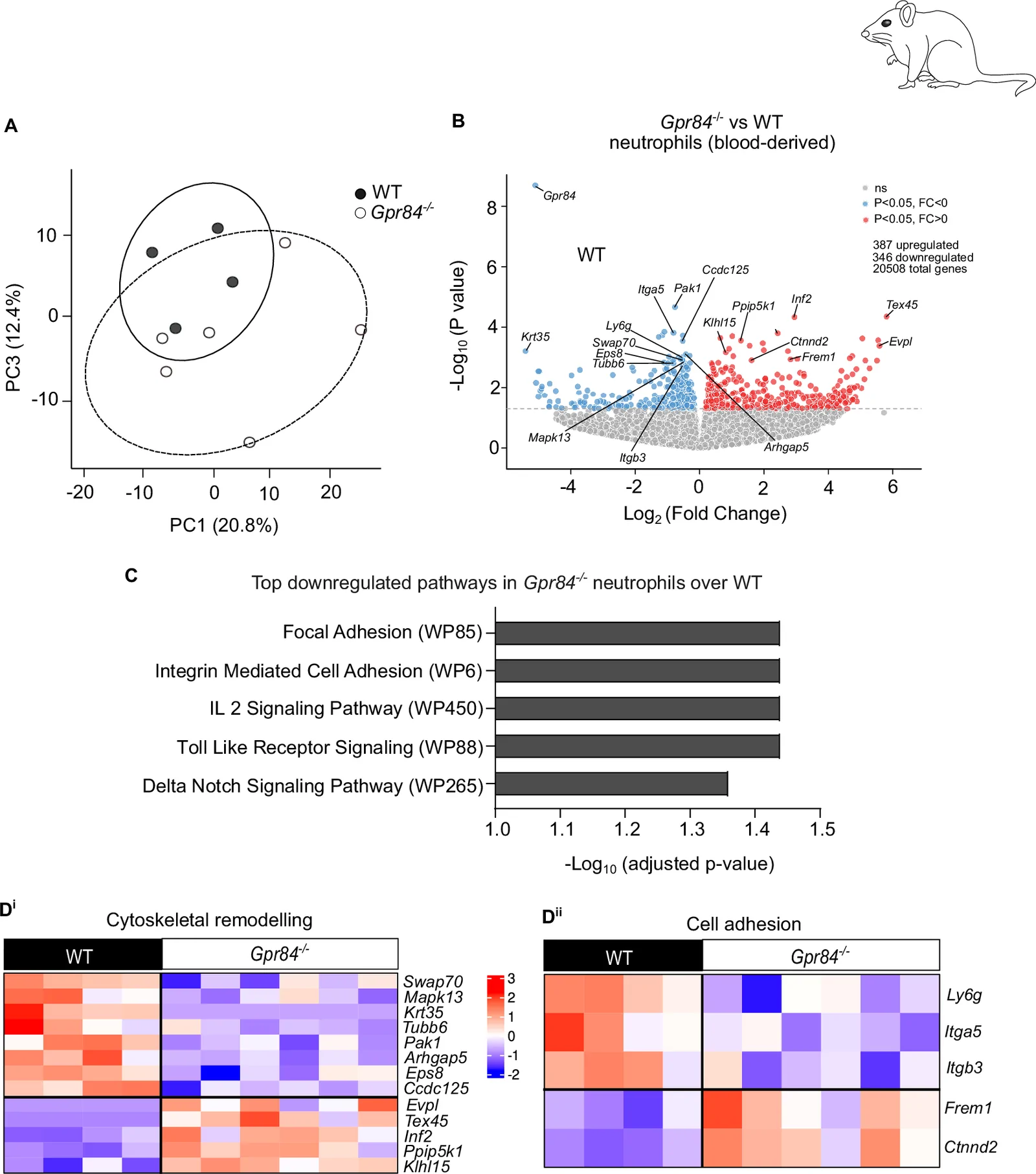
GPR84-deficient neutrophils express molecular changes in actin cytoskeletal regulation and cell adhesion. (**A**–**D**) Neutrophils were FACs sorted from the whole blood of WT (*n* = 4) or *Gpr84*^*−/−*^ (*n* = 6) mice under baseline conditions and subjected to bulk RNA-sequencing. (**A**) Principal component analysis (PCA) of transcriptomes: PC1 and PC3 describe the major variance between WT (*n* = 4) and *Gpr84*^*−/−*^ (*n* = 6) neutrophil transcriptomes. (**B**) Volcano plot of the relative difference in gene expression between *Gpr84*^*−/−*^ neutrophils (*n* = 6 mice) compared to WT (*n *= 4 mice). Labeled genes are associated with cytoskeletal remodeling and cell adhesion biological functions. The presented results are based on Fisher’s exact test with false discovery rate (FDR) adjustment. Significantly differentially expressed genes (unadjusted *P* value <  0.05) are color-coded as follows: genes downregulated in *Gpr84*^*−/−*^ over WT neutrophils are indicated in blue (fold change, FC  <  0), and upregulated genes in *Gpr84*^*−/−*^ over WT neutrophils are indicated in red (FC  >  0). (**C**) Pathway enrichment analysis (using the WikiPathways 2024 database from EnrichR) of 346 DEGs downregulated in *Gpr84*^*−/−*^ over WT neutrophils (scored as −log_10_ (*P* value)). (**D**) Mini-heatmaps show transcript levels (with an unadjusted *P* value < 0.05) of genes associated with (**D**^**i**^) cytoskeletal remodeling and (**D**^**ii**^) cell adhesion, with the scale bar representing Z-scaled gene expression across the samples.

With our live imaging studies strongly suggesting a role for Tre1/GPR84 in leukocyte detachment during extravasation, we next wished to gain insight into the likely signaling machinery involved. For this, we undertook an unbiased transcriptomic analysis of circulating WT and GPR84-deficient neutrophils. Principal Component Analysis (PCA) of data acquired from sorted blood neutrophils (isolated from untreated mice) showed a separation between WT and KO neutrophils (Fig. 6A). This revealed 713 differentially expressed genes (DEGs), with 367 significantly upregulated and 346 significantly downregulated in GPR84-deficient neutrophils relative to WT cells (Fig. 6B). Gene set enrichment analysis indicated dysregulated cytoskeletal remodeling and cell adhesion properties in GPR84-deficient neutrophils (Fig. 6C,D^i^–D^ii^), consistent with the observed phenotype of impaired detachment from the venular wall. Specifically, numerous candidates related to cytoskeletal remodeling were downregulated in GPR84-deficient neutrophils (Fig. 6D^i^), including *Pak1*, *Arhgap5, Ccdc125*—all considered essential effectors of Rho-GTPases (Shin et al, 2013; Zhao and Manser, 2005). Interestingly, GPR84-deficient neutrophils also upregulated Rho regulators such as *Pip5k1*, a kinase implicated in RhoA polarization and uropod formation in neutrophils (Xu et al, 2010).

**Figure EV3:**
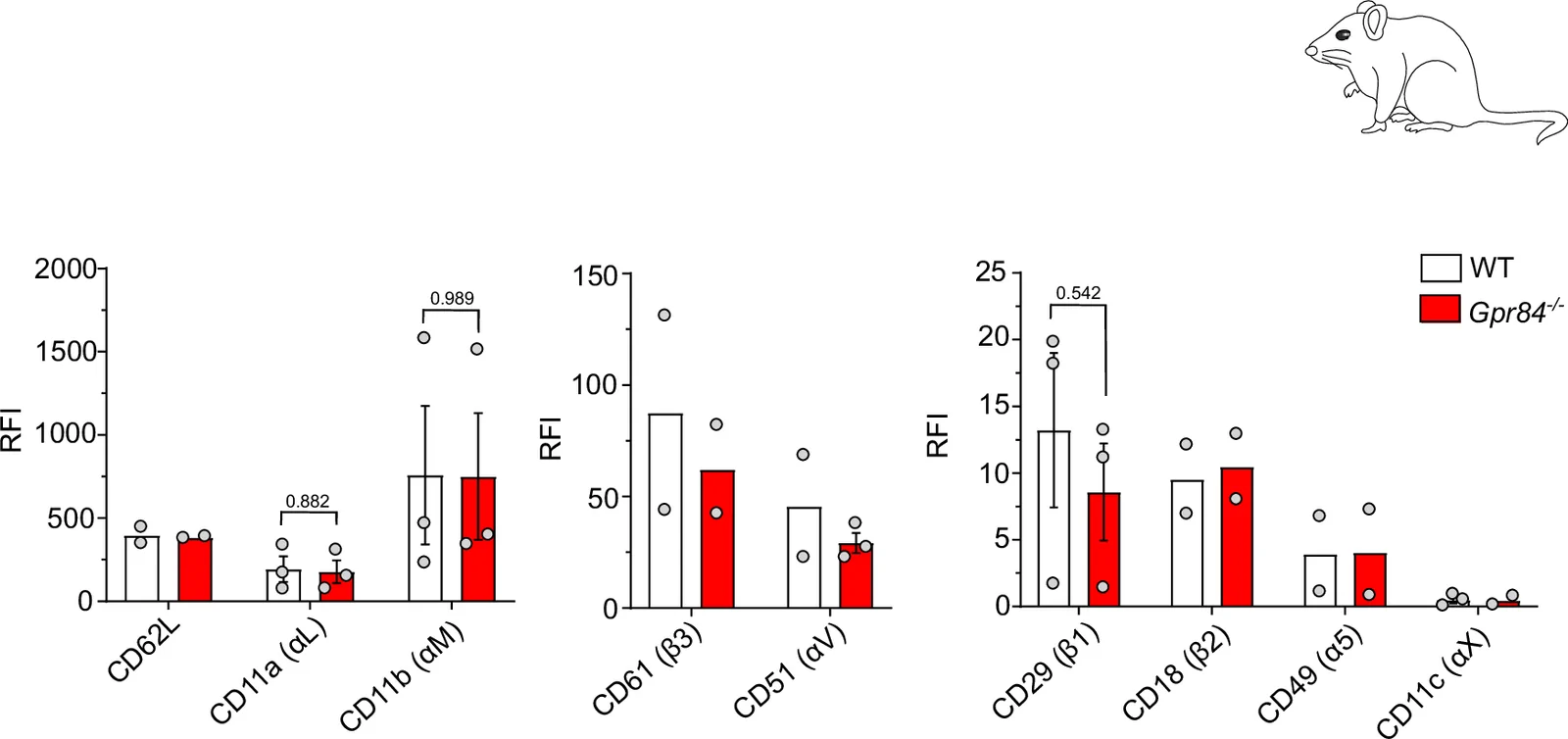
Integrin expression on WT and *Gpr84*^*−/−*^ mouse blood neutrophils. Relative fluorescence intensity (RFI) of surface expression levels of indicated integrins on mouse blood neutrophils, from WT and *Gpr84*^*−/−*^ as determined by flow cytometry (2–3 mice/group). Data represent mean ± SEM. Data were analyzed by unpaired *T* test with Welch’s correction between genotypes where applicable.

Several DEGs indicative of altered cell adhesion, most notably integrins involved in cell-cell attachment and cell-ECM adhesion, were also identified (Fig. 6D^ii^). Specific examples that were downregulated in KO neutrophils include *Itga5* that encodes for the α subunit of the integrin fibronectin receptor α5β1 (VLA-5) and *Itgb3* that encodes for the β3 integrin subunit. The latter is a component of multiple cell surface receptors that play roles in cell adhesion, migration, and signaling, and participates in RGD-dependent binding to ECM ligands (Chastney et al, 2025). Whilst suggesting defective adhesion of KO neutrophils to venular cells and basement membrane structures, we found no significant difference in the cell surface protein expression of key integrin subunits in KO vs WT neutrophils (Fig. EV3). The latter may be due to potential regulation of transcripts by negative feedback to maintain appropriate protein expression levels. KO neutrophils also showed altered expression of other genes implicated in cell migration, such as *Ly6g*, a downregulated candidate implicated in neutrophil infiltration and considered to act as a modulator of integrin functions (Song et al, 2025; Wang et al, 2012). Interestingly, KO cells displayed upregulation of *Frem1* that encodes for the generation of extracellular matrix proteins involved in the formation of basement membranes (Smyth et al, 2004). Though not previously linked to neutrophils, its upregulation may be a cellular compensatory mechanism aimed at normalizing impaired integrin-ECM interactions. Collectively, these data suggest that Tre1/GPR84 might regulate leukocyte extravasation via Rho-dependent cytoskeletal rearrangement and regulation of integrin adhesion dynamics.

### *Drosophila* Tre1 supports localized Rho1 activity during hemocyte extravasation

**Figure 7:**
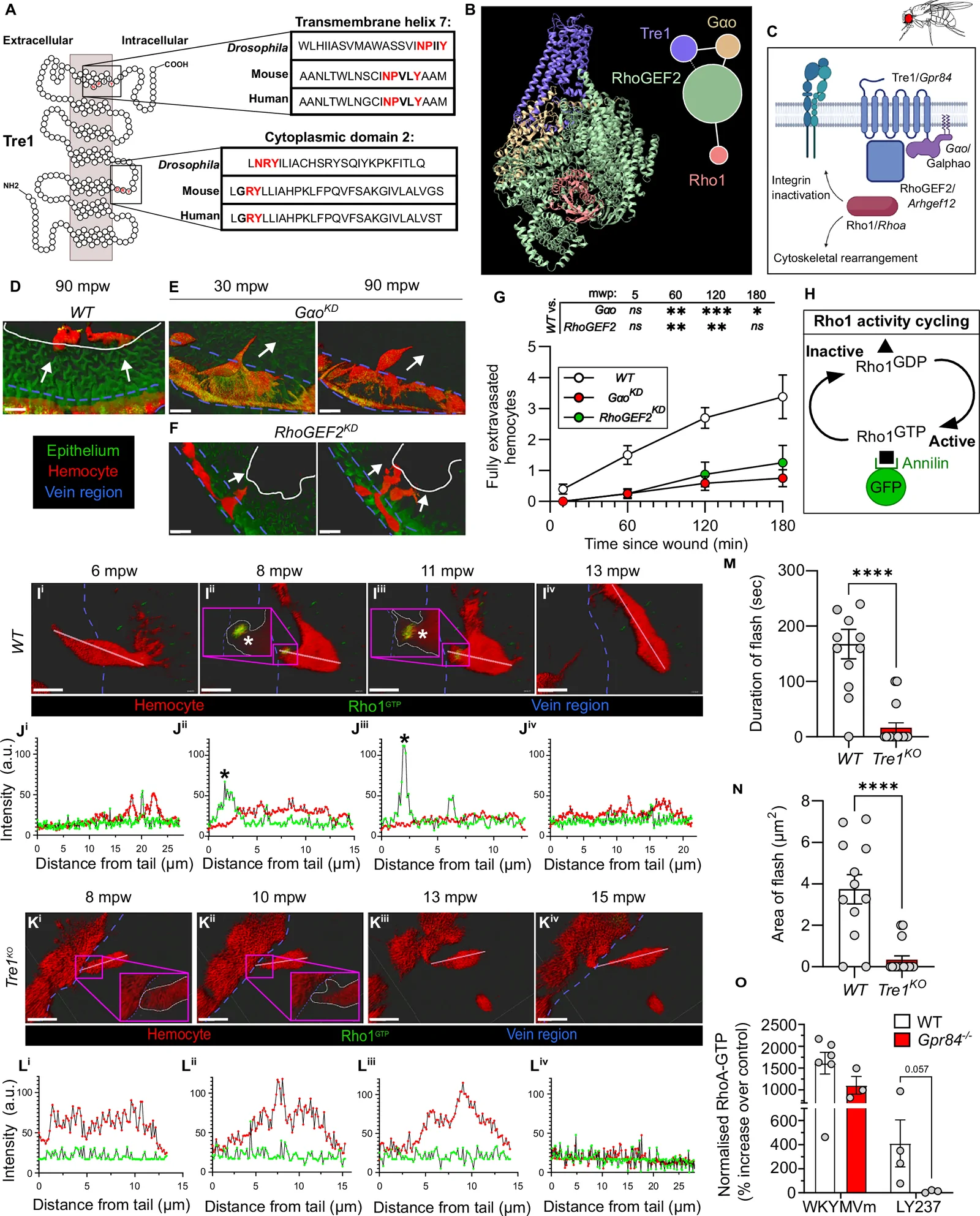
During extravasation, Tre1 signaling supports the dynamic localization of active Rho1 to hemocyte lagging tails during tail retraction. (**A**) Pearl necklace diagram of Tre1 highlighting key motifs, and how these are conserved between *Drosophila*, mice, and humans. (**B**) Structural model generated in Alphafold3 showing the predicted interface between Tre1 (blue) and Rho1 (red), with RhoGEF2 (green) and Gαo (brown) acting as intermediate accessory proteins with a calculated ipTM score of 0.3725 ± 0.0043 (mean ± SD, *n* = 4). Visualization was rendered in ChimeraX. (**C**) Proposed signaling pathway linking Tre1 to hemocyte tail retraction from the vasculature. Created in BioRender. L, C. (2025) https://BioRender.com/bup6fba. (**D**, **E**) IMARIS renderings of time-lapse confocal imaging demonstrating the requirement of Gαo for hemocyte (red) extravasation from the vasculature (blue dotted line) towards wounds (solid white outline). (**D**) *WT* hemocytes are recruited towards wounds. (**E**) *Gαo*^*KD*^ and (**F**) *RhoGEF2*^*KD*^ hemocytes both phenocopy Tre1 mutants with apparent vessel de-adhesion defects. (**G**) Quantification of the number of extravasation events between *WT* hemocytes and *Gαo*^*KD*^ (60 mpw, *P *= 0.0051; 120 mpw, *P* = 0.0002; 180  mpw, *P* = 0.0124) and *RhoGEF2*^*KD*^ (60 mpw, *P* = 0.0059; 120 mpw, *P* = 0.0061) hemocytes. Between 8 and 13 animals per genotype. (**H**) Diagram showing how the Anillin active-Rho1 biosensor reports Rho1 activity. (**I**–**L**) IMARIS renderings and intensity line plots of in vivo time-lapse imaging of *WT* (**I**^**i**^–**I**^**iv**^) and *Tre1*^*KO*^ (**K**^**i**^–**K**^**iv**^) hemocytes (red) as they extravasate from vessels, with active Rho1 (green). (**J**^**i**^–**J**^**iv**^) In *WT* hemocytes, a transient puncta of active-Rho1 localizes in the tail that resolves during tail retraction; black and white asterisks. (**L**^**i**^–**L**^**iv**^) In *Tre1*^*KO*^ hemocytes, significantly fewer active Rho1 flashes were observed. (**M**) Comparison of the time taken for active Rho1 flashes to resolve in *WT* and *Tre1*^*KO*^ hemocytes during extravasation (*P* < 0.0001) and (**N**) area of Rho1 flashes between genotypes (*P *< 0.0001). A flash/puncta was defined as any event which occurred over two consecutive time frames and with an intensity above 40 a.u. (approximately double channel background levels). Data in (**G**) were analyzed using a mixed-effects model with a Dunnett’s test performed for multiple comparisons. Data in bar plots (**M**, **N**) represent calculated means for each genotype, and dots represent individual hemocytes pooled from 12 to 16 animals per genotype; Unpaired *t* tests were performed to determine significance between genotypes. For each quantification, data represent at least five animals per genotype, and the mean ± SEM is represented. (**O**) Quantification of RhoA-GTP using a RhoA G-LISA in WT and *Gpr84*^*−/−*^ bone-marrow-derived mouse neutrophils stimulated with the FPR agonist, WKYMVm, or the GPR84 agonist, LY237. Data are presented as percentage increase over minimal readings detected in control unstimulated samples from 3 to 5 biological replicates (performed in technical replicates), with each data point acquired from neutrophils pooled from 2 to 3 donor mice and analyzed as multiple Mann–Whitney *U* tests. For (**D**–**F**), white arrows indicate the direction of travel of hemocytes towards wounds. *P < 0.05, **P < 0.01, ***P < 0.001, ****P < 0.0001. Scale bars: (**D**, **E**, **I**^**i**^–**I**^**iv**^, **K**^**i**^–**K**^**iv**^) = 10 µm. See also Movie EV7. Source data are available online for this figure.

Based on our live in vivo imaging and transcriptomic data, we speculated that Tre1/GPR84 may regulate detachment of hemocytes/neutrophils from the vessel wall by Rho-mediated events. Tre1 contains two highly conserved sequences (the E/N/DRY and NPxxY motifs), known to act via distinct signaling pathways (G-protein signaling and Rho1/E-cadherin, respectively) for different elements of the spatio-temporal guidance of germ cells (LeBlanc and Lehmann, 2017). A sequence comparison of these motifs between *Drosophila*, mouse and human highlighted that the NPxxY (so called Rho binding domain), is fully conserved between Tre1 and mammalian GPR84, whereas the E/N/DRY is not (Fig. 7A). *Drosophila* studies of migrating germ cells have shown the NPxxY motif is required for downstream Rho1 activation and that Tre1^NPxxY^ mutant germ cells demonstrate aberrant migration (Kim et al, 2021; LeBlanc and Lehmann, 2017). Our in silico AlphaFold3-mediated analysis (Abramson et al, 2024) of the predicted interface between *Drosophila* Rho1 and Tre1 suggested that Rho guanine nucleotide exchange factor 2 (RhoGEF2) and G protein alpha subunit o (Gαo) might act as intermediate accessory proteins to the stable structure (Fig. 7B). Consistent with this, Gαo has been reported to directly bind to and act downstream of Tre1 during *Drosophila* neuroblast cell polarization (Yoshiura et al, 2012); Gαo also acts downstream of Tre1’s closely-related paralogue Moody to help stabilize the extended morphology of the surface glia in the *Drosophila* blood brain barrier (Schwabe et al, 2005). RhoGEF2 is an established activator of Rho1 that acts by facilitating the switch of GDP to GTP (Mosaddeghzadeh and Ahmadian, 2021); previous work has also suggested a connection between Tre1 and RhoGEF2 during the migration of the *Drosophila* primordial germ cells (Lin et al, 2022). Since we found hemocytes and neutrophils lacking Tre1/GPR84 to exhibit similar morphological phenotypes of lagging tails and transcriptomic alteration of Rho family members, we hypothesized that these GPCRs may signal through their conserved NPxxY motifs. Consequently, we considered that the latter might then activate Rho1 via RhoGEF2 and Gαo to facilitate tail retraction/detachment during the latter stages of extravasation (Fig. 7C).

**Figure EV4:**
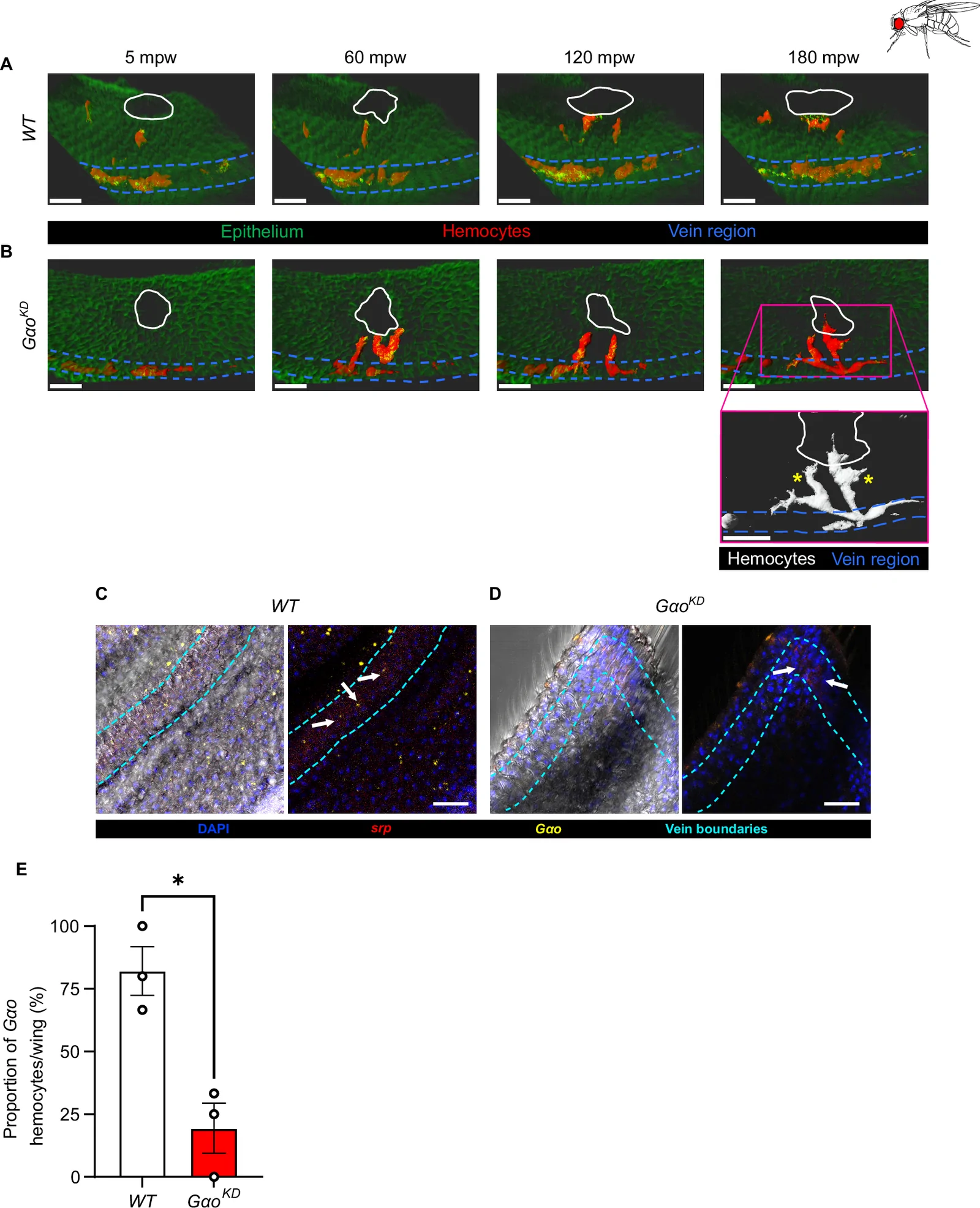
Downstream effectors of Tre1. (**A**, **B**) IMARIS renderings of 75 h APF pupae with *Gαo* silenced hemocytes (*srp-Gal4*-driven RNAi silencing) as they respond to wounds, with labeling of epithelium (green, *ubi-GMA*), hemocytes (red, *srp-Gal4>mCherry, srp-mch*), veins (manually labeled with a blue dotted line), wound perimeter (solid white line), and hemocytes attempting to extravasate (yellow asterisks). (**A**) *WT* hemocytes are recruited to the wound and fully detached from the vasculature (**B**), whereas *Gαo*^*KD*^ hemocytes show reduced recruitment and defective de-adhesion after leaving the vasculature. (**C**, **D**) Representative images of *WT* (**C**) or *Gαo*^*KD*^ (**D**) 75 h APF wings labeled by HCR in situ hybridization for *Gαo* (yellow) and the hemocyte marker *srp* (red), nuclei were counterstained with DAPI (blue), and brightfield was used to identify vasculature (manually labeled; white dotted line). In WT wings, *Gαo* was found to be lowly expressed by both hemocytes and epithelium, but reduced expression of *Gαo* was observed in the *Gαo*^*KD*^. (**E**) Quantification of the proportion of *Gαo*^*positive*^ hemocytes between *WT* and *Gαo*^*KD*^ pupae (*P* = 0.0108), validating reduced expression when using the RNAi line. In total, 53 cells from 3 wings for each genotype were analyzed, and data show mean ± SEM, which was analyzed by an unpaired *t* test. *P < 0.05. Scale bars: (**A**–**D**) = 15 µm.

To address this proposed mechanism, we utilized high-resolution in vivo imaging of extravasating *Drosophila* hemocytes in response to a laser wound. Independent analysis of *Gαo-RNAi* and *RhoGEF2-RNAi* knockdown hemocytes revealed both proteins to be essential for timely and proper hemocyte extravasation from the vasculature. Indeed, *Gαo-RNAi* and *RhoGEF2-RNAi* knockdown phenocopied *Tre1* mutants with hemocytes displaying de-adhesion defects (Figs. 7D–G and EV4A,B; Movie EV6). Expression analyses confirmed *Gαo-RNAi* caused a significant reduction in Gαo transcripts within hemocytes (Fig. EV4C–E). Utilizing a transgenic fluorescent biosensor for in vivo Rho1 activity that consists of GFP tagged to the Rho1 GTP-binding domain of Anillin (Munjal et al, 2015), we visualized the dynamics of Rho1 activity in vivo during extravasation (Fig. 7H–N; Movie EV7). As control hemocytes extravasated from vessels, we observed clear punctae of active Rho1 localized to the hemocyte tail (preceding tail retraction) that dissolved after the tail had detached (Fig. 7I,J). In Tre1-deficient hemocytes, active Rho1 was mostly absent from the tail, which remained adherent for longer and often failed to fully detach from the vein (Fig. 7K,L). Quantification of Rho1 activity (duration and area of “active Rho1” fluorescence) confirmed that *Tre1*-null hemocytes have smaller and more transient flashes of localized Rho1 activity, even on the rare occasions when they occurred (Fig. 7M,N). Similarly, using a RhoA G-LISA (G-protein Linked Immunosorbent Assay), GPR84 KO bone-marrow-derived mouse neutrophils exhibited reduced GTP-bound RhoA following stimulation with the potent GPR84 agonist L237, as compared to WT cells (Fig. 7O). Notably, within this assay, both WT and KO neutrophils responded equally to the synthetic FPR activator, WKYMVm, showing that GPR84-deficient mouse neutrophils have no intrinsic abnormalities in RhoA activation. Together, our findings demonstrate defective Rho1/RhoA activity in Tre1/GPR84-deficient immune cells in *Drosophila* and mice (Fig. 7).

### Leukocyte Tre-1/GPR84 modulate integrin-mediated adhesion

A key finding of the present study is that hemocytes and neutrophils lacking Tre1/GPR84 have long lagging tails that can persist for hours after they have attempted to leave the circulation. In control mammalian leukocytes, uropod adhesion and then retraction result from the accumulation of integrin-to-ECM focal adhesions that dissolve through a combination of contractile and biochemical forces (Hind et al, 2016). We speculated that Tre1/GPR84 facilitates tail retraction through the regulation of cytoskeletal contraction and dissolution of integrin focal adhesions mediated by Rho1/A activation. This notion is supported by previous in vitro studies showing that pharmacological blockade of RhoA leads to abnormally elongated tails in monocytes adherent to extracellular matrix (Worthylake et al, 2001), an observation that resembles Tre1/GPR84 null immune cells attempting to extravasate in vivo.

**Figure 8:**
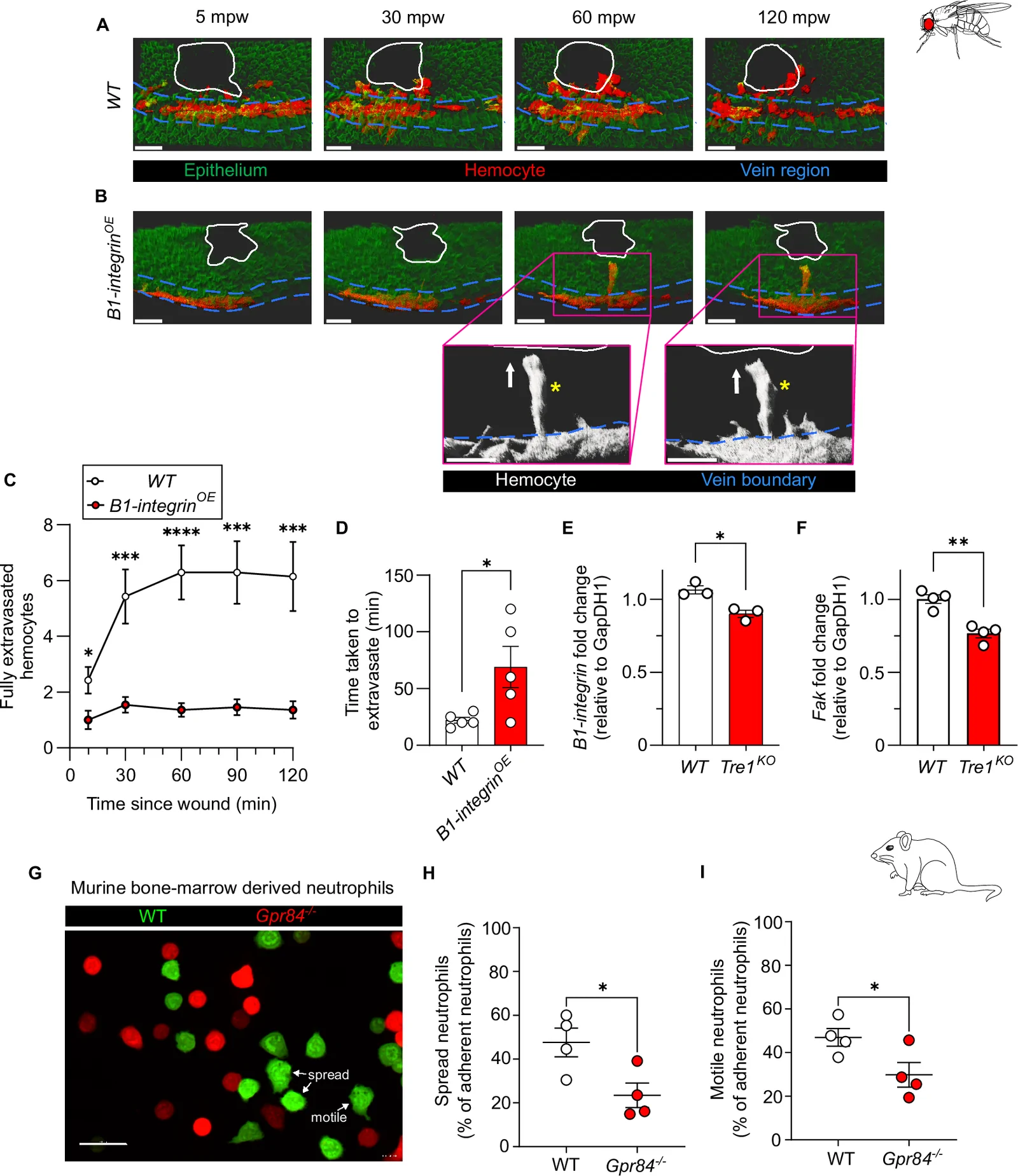
Tre1/GPR84 regulates integrin expression and functions. (**A**, **B**) IMARIS rendering of in vivo imaging of *WT* versus *myospheroid* (*ß1-integrin*) over-expressing hemocytes as they respond to wounds, with labeling of epithelium (green, *ubi-GMA*), hemocytes (red, *srp-Gal4>mCherry, srp-mch*), veins (manually labeled with a blue dotted line) and wound perimeter (solid white line). (**A**^**i**^–**A**^**iv**^) *WT* hemocytes are recruited to the wound and fully detach from the vasculature (**B**^**i**^–**B**^**iv**^), whereas *ß1-integrin*^*OE*^ hemocytes show reduced recruitment, a lagging tail phenotype with long protrusions (yellow asterisks) towards the wound, tethering to the vasculature. White arrows indicate the direction of travel of hemocytes towards wounds. (**C**) Quantification of numbers of hemocytes that have fully dissociated from veins and extravasated toward wounds between *WT* and *ß1-integrin*^*OE*^ genotypes (5 mpw, *P* = 0.0220; 30 mpw, *P* = 0.0003; 60 mpw, *P* < 0.0001; 90 mpw, *P* = 0.0001; 120 mpw, *P* = 0.0003). Each genotype consisted of five animals. (**D**) Analysis of time taken for individual hemocytes to extravasate from veins in both *WT* and *ß1-integrin*^*OE*^ genotypes (*P* = 0.0254). Each genotype consisted of five animals. (**E**, **F**) Quantitative RT-PCR of the relative expression of *B1-integrin* (*P* = 0.0118) and *Fak* (*P* = 0.0014) in whole 75 h APF pupae in both *WT* and *Tre1*^*KO*^ backgrounds, with both target genes’ threshold cycle values normalized to Gapdh1; in the *Tre1*^*KO*^ background, both genes show a significant decrease in expression levels. RT-PCR data represented in a bar plot is mean ± SEM overlayed with the average value (red dots) for each biological replicate (RNA pooled from 10 individuals). (**G**–**I**) Bone marrow-derived WT (green) and *Gpr84*^*−/−*^ (red) mouse neutrophils were mixed and seeded onto ICAM-1-coated surfaces and stimulated with CXCL1 for 15 min. (**G**) Representative confocal images illustrating the cellular morphology of WT and KO neutrophils, and quantified proportion of (**H**) “spread” (*P* = 0.0307) and (**I**) “motile” (*P* = 0.0481) adherent neutrophils from *n* = 4 mice/group as biological replicates. Data show mean ± SEM. Data in (**C**) were analyzed by multiple unpaired *T* tests followed by correction for multiple comparisons using the two-stage step-up method of Benjamini, Krieger, and Yekutieli. Data in (**D**–**F**, **H**, **I**) were analyzed by unpaired *T* tests. *P < 0.05, **P < 0.01, ***P < 0.001, ****P < 0.0001. Scale bar: (**A**^**i**^–**A**^**iv**^, **B**^**i**^–**B**^**iv**^) = 15 µm; (**G**) = 20 µm. Source data are available online for this figure.

**Figure EV5:**
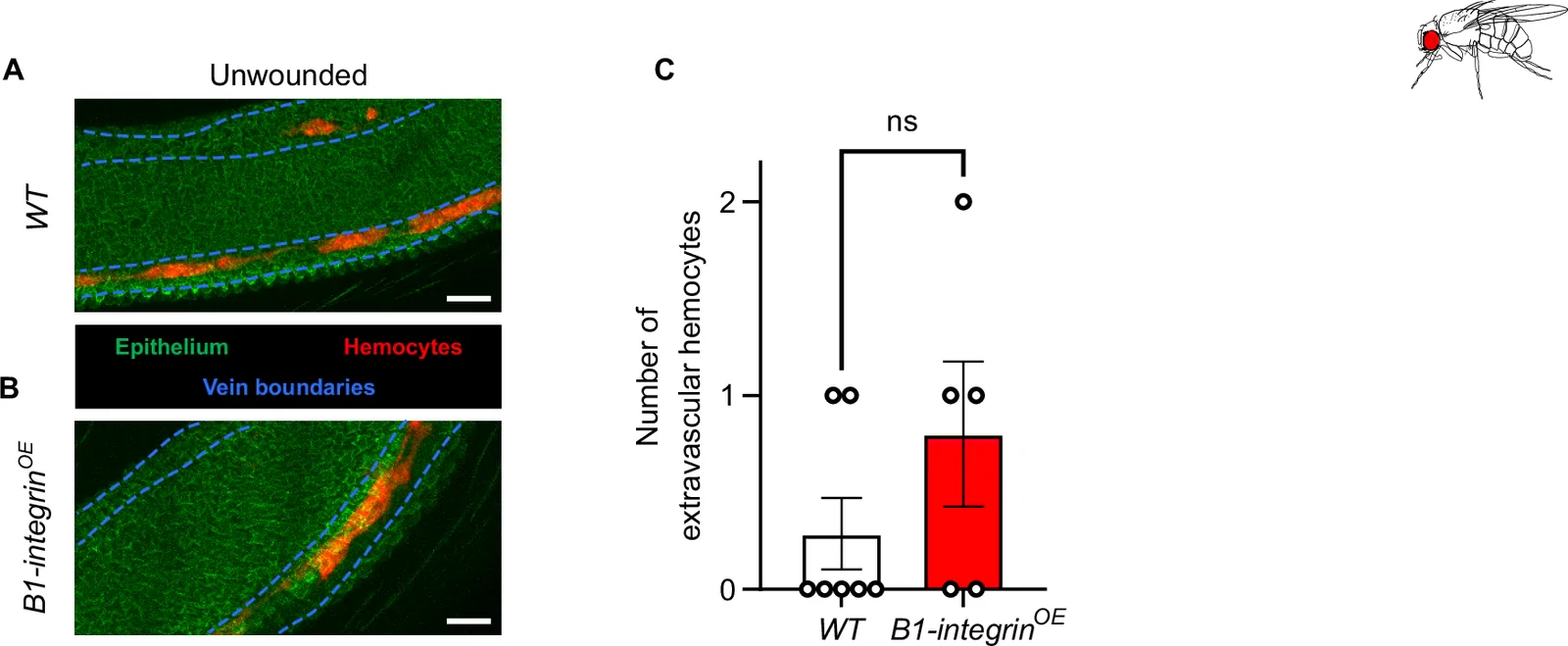
Overexpression of ß1-integrin in hemocytes does not impact their tissue distribution. (**A**, **B**) Representative images of 75 h APF wing vasculature with no significant difference in the number of extravascular hemocytes between *WT* (**A**) and *ß1-integrin*^OE^ (**B**) hemocytes. Extravascular refers to hemocytes, which are outside of the vasculature. (**C**) Quantification of the number of extravascular hemocytes in *WT* and *B1-integrin*^OE^ genetic backgrounds. Between 5 and 7 animals were analyzed for each genotype. Data represent mean ± SEM, which was analyzed by an unpaired Mann–Whitney test. Scale bars: (**A**, **B**) = 15 µm.

In hypothesizing that activated Tre1 dissolves integrin adhesions in the hemocyte tail, we predicted that elevating hemocyte integrin levels would phenocopy Tre1-deficient hemocytes. Indeed, we observed that overexpression of *myospheroid* (*mys*; the predominant *Drosophila* β-integrin) specifically in hemocytes led to a strikingly similar defective extravasation phenotype (Fig. 8A–D) despite not impacting the number of extravascular hemocytes pre-wound (Fig. EV5A–C). To further address whether Tre1/GPR84 regulates focal adhesion dissolution in lagging tails as leukocytes extravasate, we examined whether *mys* levels were perturbed in *Drosophila* lacking Tre1. We found that in *Tre1* null pupae, *mys* mRNA levels were significantly reduced, as were transcripts for *focal adhesion kinase* (*Fak*), a known regulator of integrins (Fig. 8E,F). These data are consistent with the transcriptomic analysis of GPR84 KO mouse neutrophils, suggesting a conserved role for Tre1/GPR84 in modulating integrin-based adhesion (Fig. 6C), and our observations of defective adhesion of GPR84 mouse KO neutrophils to intercellular adhesion molecule 1 (ICAM-1)-coated plates (Fig. 8G–I). ICAM-1 is highly expressed on TNF-stimulated endothelial cells and pericytes (Proebstl et al, 2012; Cooper et al, 2024; Recio et al, 2018; Yousefi et al, 2001; Bouchard et al, 2007) and was thus used as an in vitro adhesive substrate to functionally mimic neutrophil interactions with vascular cells. Following stimulation with the chemokine CXCL1, whilst the average surface area of WT and KO neutrophils was not significantly different (64.42 µm^2^ ± 7.42 and 53.88 µm^2^ ± 6.74, WT and KO neutrophils, respectively; *n* = 283 WT and *n* = 186 KO neutrophils, from *n* = 4 mice, *P* = 0.2582), the frequency of *Gpr84*^*−/−*^ neutrophils that displayed a **“**spreading” morphology was significantly reduced (Fig. 8G,H). This was associated with an overall defective motility in vitro (Fig. 8I) and observed reduced membrane ruffling and polarization, compared to controls (Fig. 8G), suggesting an impairment in integrin-induced cytoskeletal remodeling in response to activation.

Collectively, the data acquired from our *Drosophila* and mouse studies, using both in vitro and in vivo assays, strongly support the concept that Tre1/GPR84 exhibits conserved functionalities in supporting Rho-mediated regulation of integrin activity.

### Pharmacological blockade of GPR84 suppresses neutrophil extravasation in acute inflammation

**Figure 9:**
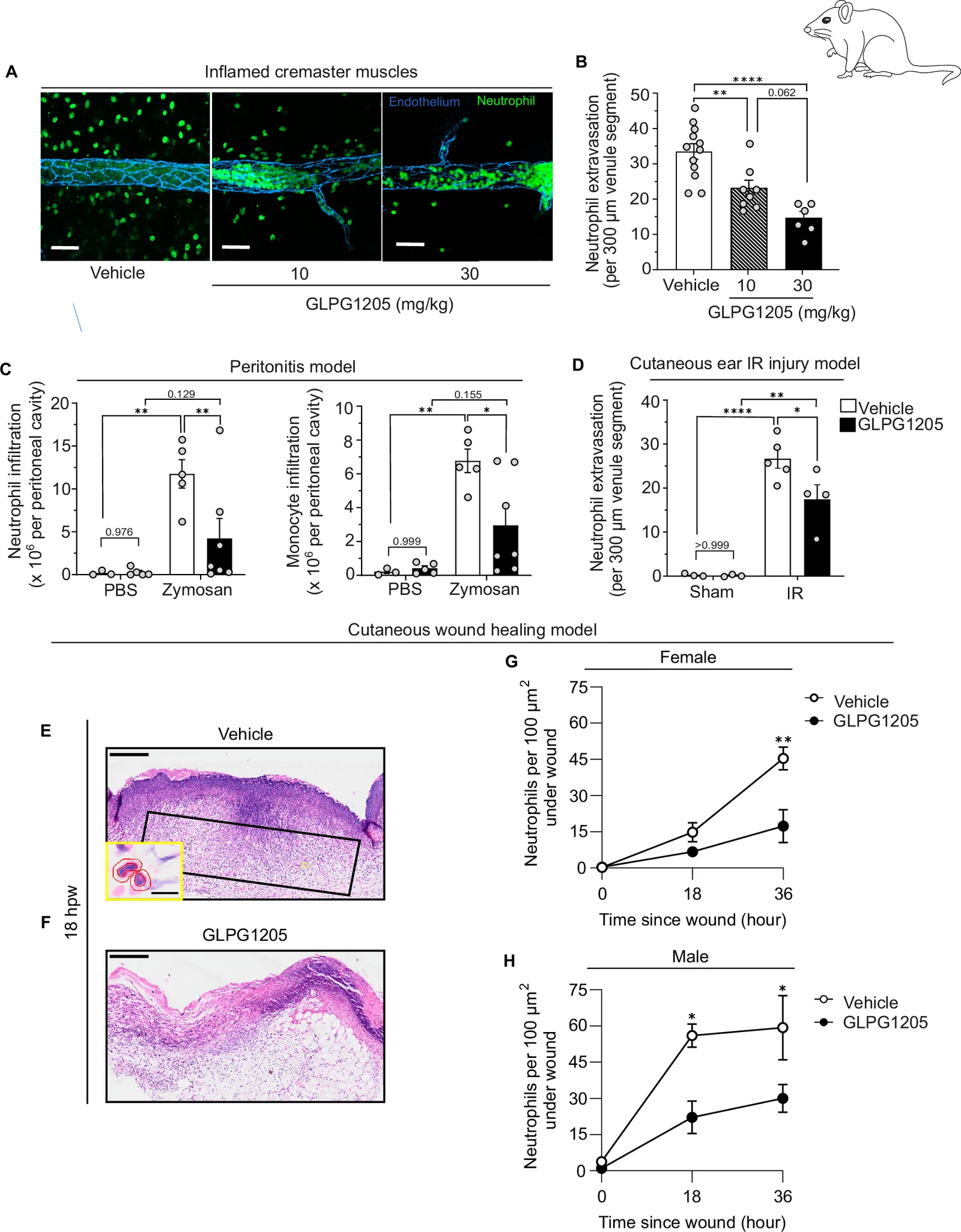
Pharmacological blockade of GPR84 suppresses neutrophil extravasation in models of acute inflammation. (**A**) Representative images of TNF-stimulated (4 h) cremasteric venules of C57Bl6/J mice pre-treated (oral gavage, 24 h before) with vehicle or GLPG1205 (10 or 30 mg/kg). Tissues were fixed, permeabilized, and stained for endothelial cells (PECAM-1; blue) and neutrophils (MRP-14; green). (**B**) Quantification of the average number of extravasated neutrophils per 300 µm venule segment from 4 to 11 venules per mouse; *n* = 6–12 mice/group. Data compares mice treated with vehicle with mice treated with GLPG1205 at 10 mg/kg (*P* = 0.0066) or 30 mg/kg (*P* < 0.0001). (**C**) C57BL/6 mice were treated with vehicle or GLPG1205 (30 mg/kg) 24 h prior to an i.p. injection of zymosan or PBS (control). The infiltration of neutrophils and monocytes at 24 h post injection was quantified by flow cytometry (*n* = 5–8 mice/group). Data (left panel) compares neutrophil infiltration in vehicle-treated mice stimulated with i.p. PBS or zymosan (*P* = 0.0017) and i.p. zymosan stimulated groups in mice treated with vehicle or GLPG1205 (*P* = 0.0075). The monocyte infiltration data (right panel) compares vehicle-treated mice stimulated with i.p. PBS or zymosan (*P *= 0.0014) and i.p. zymosan stimulated groups in mice treated with vehicle or GLPG1205 (*P* = 0.0185). (**D**) C57BL/6 mice were treated with vehicle or GLPG1205 (30 mg/kg) 24 h prior to ear ischemia reperfusion injury. Quantification of the average number of extravasated neutrophils from 5 to 12 venule segments per mouse (with *n* = 3–5 mice/group). Data compare vehicle-treated mice subject to sham or IR injury (*P* < 0.0001), GLPG1205-treated mice subject to sham or IR injury (*P *= 0.0019), and vehicle and GLPG1205-treated mice subject to IR injury (*P* = 0.0486). (**E**, **F**) Hematoxylin and eosin-stained wax sections of back skin wounds at 18 h post wounding (hpw). (**E**) Representative image of an 18 h wound from a vehicle-treated female mouse. The black box indicates the approximate region for neutrophil quantification, and the yellow box indicates the site of the inset, highlighting automated capture and quantification of the numbers of neutrophil nuclei. (**F**) Representative image of an 18 h wound from a GLPG1205-treated female mouse. (**G**, **H**) Quantification of neutrophil extravasation at different time-points in the wound beds of vehicle versus GLPG1205-treated mice (*n* = 3 mice/group/timepoint). (**G**) In females, the number of neutrophils at the wound bed was only significantly less in GLPG1205-treated mice 36 h after wounding (*P* = 0.0012). (**H**) Whereas, male mice treated with GLPG1205 had significantly less neutrophils at the wound bed earlier (18 h, *P* = 0.0127; 36 h, *P* = 0.0308). Data in (**G**, **H**) shows mean ± SEM. Data in (**B**) was analyzed by one-way ANOVA with a Tukey’s test for multiple comparisons, and (**C**, **D**) were analyzed by two-way ANOVA with Uncorrected Fisher’s LSD or Tukey’s multiple comparisons post hoc test. (**G**, **H**) were analyzed by unpaired *T* tests followed by correction for multiple comparisons using Šídák’s test. *P < 0.05, **P < 0.01. Scale bars: (**A**) = 50 µm; (**E**, **F**) = 250 µm: (**E**) inset 10 µm. Source data are available online for this figure.

Given the conserved role for GPR84/Tre1 in supporting effective immune cell extravasation, in a final series of experiments, we explored the effects of pharmacologically modulating murine GPR84 during acute inflammation. For this purpose, we tested the efficacy of a selective and orally active functional GPR84 antagonist (GLPG1205) in multiple murine models of acute inflammation. Initially, using our TNF-inflamed cremaster muscle model, a reaction in which the GPR84 KO mice exhibited ~70% inhibition of leukocyte migration (Fig. 3D), we found that treatment of mice with the antagonist led to a dose-dependent suppression of neutrophil extravasation (Fig. 9A,B). Using the higher dose of the antagonist (30 mg/kg), these findings were extended to three other inflammatory settings, namely a zymosan-induced peritonitis model, a cutaneous ischemia–reperfusion (IR) injury model, and finally a skin wounding assay. In the peritonitis model, a well-characterized self-resolving acute inflammatory reaction, 24 h post injection of zymosan, GLPG1205 pre-treated mice exhibited reduced peritoneal cavity infiltration of both neutrophils and monocytes (Fig. 9C). These results suggest that GPR84 can mediate appropriate migration of multiple populations of myeloid cells. The cutaneous IR injury model, employed as a model of a pressure ulcer, similarly showed an effective neutrophil infiltration response in control mice that was significantly suppressed in mice treated with the GPR84 antagonist (Fig. 9D). The latter studies included both male and female mice, demonstrating that the efficacy of GPR84 blockade was not sex specific.

GPR84 expression increases in murine myeloid cells (neutrophils, macrophages, and monocytes) following skin wounding (Cooper et al, 2024). To investigate how wound inflammation might be impacted by GPR84 signaling, we generated punch biopsy skin wounds in GLPG1205-treated versus control adult male and female mice (Fig. 9E–H). Standardly, the initial influx of neutrophils to such a skin wound begins from 6 h post wounding, with numbers peaking at 24 h and diminishing back to basal levels after 48 h, as macrophage numbers begin to predominate in the wound. To capture potential differences in neutrophil influx in treated versus control mice, we harvested wounds at 18 and 36 h and analyzed wound sections stained with Hematoxylin and Eosin (Fig. 9E,F); here, the automated software QuPath (Bankhead et al, 2017) enabled efficient quantification of Hematoxylin-stained neutrophil nuclei within Eosin-stained cytoplasms (inset, Fig. 9E). Our data show a clear dampening of neutrophil influx into the wound bed at both timepoints in female (Fig. 9G) and male (Fig. 9H) mice.

Collectively, using a pharmacological approach, these results demonstrate the importance of GPR84 in regulating leukocyte extravasation in several inflammatory contexts in different organs, in response to different stimuli, and in both male and female animals.

## Discussion

Whilst the molecular and cellular pathways that regulate the onset of leukocyte extravasation across vessel walls are increasingly well-characterized, the complex signaling events that enable successful completion of the sequential steps in vessel breaching remain unclear. Here, we identified the orphan GPCR, Tre1/GPR84, as pivotal for effective termination of leukocyte extravasation during acute physiological and pathological inflammation. Specifically, by revealing how leukocytes deficient in these GPCRs retain long, adherent tethers and fail to complete extravasation in vivo, we demonstrate their functional role in effective leukocyte tail detachment from vessel walls. Crucially, our murine studies demonstrated a role for GPR84 in multiple stages of neutrophil extravasation, consistently indicative of regulating neutrophil de-adhesion from vascular cells, endothelial cells, and pericytes, as well as potentially the venular basement membrane. Mechanistically, transcriptomic analyses together with intravital imaging of subcellular signaling events revealed that Tre1/GPR84 regulated leukocyte cytoskeletal and adhesion dynamics, specifically supporting localized Rho1 activity in retracting leukocyte tails. Moreover, pharmacological antagonism of murine GPR84 in vivo dampened leukocyte infiltration in a range of acute inflammatory conditions in both sexes, including zymosan-induced peritonitis and skin wounding. Collectively, these data identify GPR84 as a previously unknown regulator of neutrophil extravasation and present data to suggest its potential as a promising anti-inflammatory therapeutic target.

GPR84 is widely considered a pro-inflammatory molecule as its expression is increased in myeloid cells (particularly monocytes and macrophages) in response to multiple inflammatory stimuli (Cooper et al, 2024; Recio et al, 2018; Yousefi et al, 2001; Bouchard et al, 2007; Nagasaki et al, 2012). Furthermore, studies have reported on the ability of GPR84 to mediate leukocyte infiltration into inflammatory sites, and indeed this receptor has been the subject of much interest as a therapeutic anti-inflammatory target (Cooper et al, 2024; Recio et al, 2018; Suzuki et al, 2013; Mancini et al, 2019; Wang et al, 2023). Despite the significant attention given to this molecule for >15 years, the mechanism through which it mediates leukocyte trafficking has remained unknown. Here, we demonstrate a specific role for Tre1/GPR84 in leukocyte (*Drosophila* hemocyte and murine neutrophil) migration, a phenomenon dramatically illustrated during extravasation across vessel walls in acute inflammation. During this response, we propose that Tre1/GPR84 signaling acts via Gαo and RhoGEF2 to promote dynamic Rho1 activation within the leukocyte tail; Rho1 activity in turn promotes cytoskeletal and adhesion remodeling that enables the leukocyte to fully detach its lagging tail from the vessel wall. In the absence of effective Tre1/GPR84 signaling, despite the leukocyte initiating the process of extravasation, the lack of Rho1-driven de-adhesion leads to decelerated breaching of vessel walls, culminating in a striking elongated leukocyte phenotype in the perivascular region. The resultant impeded extravasation led to a notable accumulation of ‘stuck’ leukocytes around the inflamed vessel. Interestingly, our data suggest Tre1/GPR84 may be involved in regulating adhesion at multiple steps during leukocyte migration, including intravascular crawling, as we observed increased hemocyte luminal adherence and reduced circularity in the absence of Tre1 and reduced slow crawling of GPR84 KO neutrophils on inflamed venular endothelium. Nevertheless, while Tre1/GPR84 might impact leukocyte adhesion levels during all migratory responses, more pronounced effects might be apparent during events that require stronger detachment, such as extravasation. Intriguingly, despite Tre1/GPR84-deficient leukocytes exhibiting a more adhesive cellular phenotype, we found a marked transcriptomic downregulation of adhesion molecules (particularly integrin subunits) within Tre1/GPR84-deficient leukocytes. This raises the possibility that null leukocytes downregulate adhesion-related gene expressions to compensate for the elevated adhesion response.

Though it is widely accepted that tightly controlled spatio-temporal regulation of integrin-mediated leukocyte adhesion is essential for effective extravasation (Nourshargh and Alon, 2014), the precise associated mechanisms remain unclear. Integrins play established roles in driving the initial steps of neutrophil extravasation, with activation of defined β2 integrins mediating distinct intraluminal responses, including LFA-1-dependent adhesion followed by Mac-1-dependent crawling (Phillipson et al, 2006). In addition, there is ample evidence for the involvement of integrins in supporting neutrophil breaching of vascular components, ECs, pericytes, and the venular basement membrane (Nourshargh and Alon, 2014; Girbl et al, 2018). Finally, prior to full exit from the venular wall, neutrophils (and other immune cells) exhibit elongated uropods in the perivascular space, a phenomenon mediated through integrin-mediated attachment of immune cells to vascular structures (Hyun et al, 2012); here, it has been shown that a coordinated dissociation of LFA-1 from the endothelium and VLA-3-mediated migration through the basement membrane enables timely de-adhesion and propulsion into the interstitial tissue.

Our work is also consistent with earlier seminal studies demonstrating that monocyte adhesive behavior is critically regulated by Rho activity (Worthylake et al, 2001; Aepfelbacher et al, 1996). Monocytes require RhoA-dependent uropod retraction to release β2 integrin-mediated adhesion during transendothelial migration as well as migration on a solid substratum (Worthylake et al, 2001). Indeed, RhoA inhibition in monocytes blocked tail retraction during transendothelial migration in a manner strikingly reminiscent of GPR84-deficient neutrophils, with monocytes exhibiting β2 integrins localized on stuck, elongated trailing uropods (Worthylake et al, 2001). In an independent study, Rho inactivation increased the spreading of human monocytes on fibronectin and suggested that monocyte Rho activity might antagonize cell spreading by maintaining cell tension and cortical actin organization (Aepfelbacher et al, 1996). Our findings enhance our knowledge of these processes by presenting evidence for Tre1/GPR84 as an essential component of leukocyte de-adhesion, potentially inducing the dissolution of β2 integrin complexes via Rho-signaling at the terminal stages of neutrophil extravasation.

Despite improved understanding of the intracellular events downstream of activated Tre1/GPR84, the identity of the upstream signals mediating leukocyte GPR84 activation (including its precise ligand) remains unclear. Studies of the *Drosophila* germ cells reveal that the Wunens, germ cell-expressed membrane proteins that can dephosphorylate and inactivate phospholipids, may modulate Tre-1 signaling by creating a phospholipid gradient (Chen et al, 2025). In the mammalian system, GPR84 has been proposed as a medium-chain fatty acid (MCFA) receptor, whereby MCFAs promote leukocyte migration, phagocytosis, and release of pro-inflammatory cytokines (e.g., TNF) (Suzuki et al, 2013; Nagasaki et al, 2012; Wang et al, 2006; Hu et al, 2008). Intriguingly, our transcriptomics data revealed an upregulation of the dual-functional inositol kinase Ppip5k1 in *Gpr84*-deficient neutrophils. *Drosophila* dPip5k generates PI(4,5)P2 and plays an important role in polarizing the actin cytoskeleton for the directed migration of embryonic germ cells, in cooperation with Tre1 (Kim et al, 2021). Signaling via phospholipids may thus play conserved roles modulating leukocyte Tre1/GPR84 activity in acute inflammatory settings in vivo.

Comparative transcriptomic profiling of control versus GPR84-deficient neutrophils revealed numerous biological pathways that could be regulated by GPR84. Whilst cytoskeletal remodeling and cellular (focal and integrin-mediated) adhesion pathways were amongst the most downregulated pathways in GPR84 KO cells, neutrophils lacking GPR84 also downregulated other key immune pathways (including IL2 and Toll Like Receptor Signaling). IL2 signaling within neutrophils has been suggested to inhibit their migration, increase their adherence to ECs, and stimulate neutrophil antimicrobial activities, including the respiratory burst and degranulation (Li et al, 1996; Kowanko and Ferrante, 1987). Our transcriptomic data show that components of the Delta/Notch signaling pathway were also downregulated by GPR84 deficiency. Whilst the role of Notch signaling in myeloid lineages (particularly neutrophils) remains poorly understood, evidence suggests Toll-Like Receptor (TLR) signaling can induce Notch receptor and ligand expression, and these pathways may coordinate to fine-tune leukocyte activation and function (Hildebrand et al, 2018; Hu et al, 2008). Notch signaling has also been linked to myeloid cell development and differentiation (Schroeder and Just, 2000), suggesting GPR84 may modulate leukocyte development, consistent with our observed downregulation of *Ly6g* (a candidate reflective of neutrophil maturity) in GPR84-deficient neutrophils. These data suggest GPR84 may have additional functions in leukocytes beyond Rho1-mediated regulation of leukocyte adhesion.

Given that GPCRs are major therapeutic targets—accounting for nearly 35% of all clinically used drugs—GPCR antagonists are considered as plausible strategies for selective modulation of leukocyte trafficking in numerous inflammatory conditions (Sun and Ye, 2012). In line with this, there exists considerable therapeutic interest in GPR84 as a potential druggable target for various inflammatory conditions, despite its molecular mechanisms remaining largely unknown. Here, our data adds important mechanistic insight into GPR84’s translational potential. Our in vivo pharmacological studies revealed the efficacy of a GPR84 functional antagonist (GLPG1205) in dampening neutrophil infiltration in multiple experimental disease models, including skin wounding and peritonitis. This is consistent with recent work identifying GPR84 as a key regulator of myeloid cell (particularly macrophage) recruitment during murine skin wound healing (Cooper et al, 2024). Intriguingly, GPR84 levels are also increased in murine models of diabetes and fibrosis (Gagnon et al, 2018; Recio et al, 2018), with inhibition of GPR84 signaling reducing fibrosis in a range of tissues (Simard et al, 2020; Puengel et al, 2020; Gagnon et al, 2018). GPR84 has also been identified as a biomarker of burn injury exhibiting increased expression on blood neutrophils isolated from human patients (Hou et al, 2024). Recently, GPR84 inhibition even accelerated the repair of murine ulcerative lesions, including tissue damage associated with *Mycobacterium ulcerans* infection (Foulon et al, 2026). These studies, together with our work, indicate how GPR84 signaling might contribute to numerous inflammatory pathologies, including chronic non-healing wounds, peritonitis, acute respiratory distress syndrome, and ulcerative colitis (Abdel-Aziz et al, 2016; Yin et al, 2020; Zhang et al, 2022).

In summary, we have exploited the unique collective tractability of our *Drosophila* and murine models for rapid mechanistic interrogation of complex cell-specific signaling networks in vivo. With the focus of these studies being on the regulation of leukocyte extravasation across vessel walls, integrating our *Drosophila* studies with murine models that enable intravital imaging of mammalian leukocyte extravasation has proven to be a powerful approach for identifying new, conserved, and fundamental molecular players of acute inflammation. Capitalizing on *Drosophila’s* unrivaled potential for combining in *vivo* real-time imaging of subcellular signaling events with cell type-specific genetics, we have identified a conserved role for GPR84 orthologs in mediating localized leukocyte de-adhesion and cytoskeletal dynamics during extravasation. In the long term, we envision that the unique imaging and large-scale screening opportunities of *Drosophila* will enable the discovery of more genes with important conserved regulatory functions in immune cell extravasation, both within immune cells and the vascular cells with which they interact.

## Methods

**Table:** Reagents and tools table

Reagent/resource	Reference or source	Identifier or catalog number
**Experimental models**		
*Lyz2-EGFP-ki* (*M. musculus*)	Dr Thomas Graf (Center for Genomic Regulation and ICREA, Spain)	
C57/Bl6J (*M. musculus*)	Charles River	
*Gpr84*^*−/−*^ (*M. musculus*)	Nicol et al, 2015	B6N.Gpr84tm1(Dgen)Crl
*Lyz2-EGFP-ki; Acta2-RFPcherry-Tg* (*M. musculus*)	Proebstl et al, 2012	
*Gpr84*^*-/-*^*; Lyz2-EGFP-ki; Acta2-RFPcherry-Tg* (*M. musculus*)	This study	
*CD45.1* (*M. musculus*)	Dr Suchita Nadkarni (William Harvey Institute, Queen Mary University London)	B6.SJL-Ptprca Pepcb/BoyJ
*ubi-GMA, srp-gal4* (*D. melanogaster)*	Ref: Brückner et al, 2004	
*srp-mcherry* (*D. melanogaster)*	Gift from Daria Siekhaus	
*;;Tre1-RNAi* (*D. melanogaster)*	Bloomington *Drosophila* Stock Center	#33718
*Oregon R* (*D. melanogaster)*	Lab stock	
*AniRBD* (*D. melanogaster)*	Ref: Munjal et al, 2015	
*Tre1*^*KO*^ (*D. melanogaster)*	Ref: Kim et al, 2021	
*UAS-mys* (*D. melanogaster)*	Bloomington *Drosophila* Stock Center	#68158
*Gαo-RNAi* (*D. melanogaster)*	Bloomington *Drosophila* Stock Center	#34653
**Antibodies**		
CD31 (PECAM-1) Monoclonal Antibody (390), Functional Grade, eBioscience™	Thermo Fisher Scientific	Cat# (16-0311-85)
Anti-MRP-14	Gift from Dr Nancy Hogg (Francis Crick Institute, UK)	
Zombie Yellow™ fixable Viability kit	Biolegend	Cat# 423103
CD16/CD32 Monoclonal Antibody (93), eBioscience™	Thermo Fisher Scientific	Cat# 14-0161-85
PE Rat IgG2b, κ Isotype Ctrl Antibody	BioLegend	Cat# 400607
PE Armenian Hamster IgG Isotype Ctrl Antibody	BioLegend	Cat# 400907
PE Rat IgG1, κ Isotype Ctrl Antibody	Biolegend	Cat# 400407
Anti-CD45 PB (30-F11)	BioLegend	Cat# 103126
Anti-CD3 PE (17A2)	BioLegend	Cat# 100205
Anti-CD19 AF488 (6D5)	BioLegend	Cat# 115524
AF488 Rat IgG2a, κ Isotype Ctrl Antibody	BioLegend	Cat# 400525
Anti-CD11b PE/Cy7 (M1/70)	BioLegend	Cat# 101216
PE/Cy7 Rat IgG2b, κ Isotype Ctrl Antibody	BioLegend	Cat# 400617
Anti-Ly6C PerCP-Cy5.5 (HK1.4)	BioLegend	Cat# 128012
PerCP/Cyanine5.5 Rat IgG2c, κ Isotype Ctrl Antibody	BioLegend	Cat# 400723
Anti-Ly6G AF647 (1A8)	BioLegend	Cat# 127610
AF647 Rat IgG2a, κ Isotype Ctrl Antibody	BioLegend	Cat# 400525
Anti-F4/80 PE (BM8)	BioLegend	Cat# 123110
PE Rat IgG2a, κ Isotype Ctrl Antibody	Invitrogen	Cat# 12-4321-80
Anti-CD115 APC-Cy7 (AFS98)	BioLegend	Cat# 135531
APC-Cy7 Rat IgG2a, κ	Biolegend	Cat# 400523
Anti-CD62L PE (MEL-14)	BioLegend	Cat# 104407
Anti-CD11a PE (M17/4)	BioLegend	Cat# 101107
Anti-CD11b PE (M17/0)	BioLegend	Cat# 101207
Anti-CD61 PE (2C9.G2 (HMβ3-1))	BioLegend	Cat# 104307
Anti-CD51 PE (RMV-7)	BioLegend	Cat# 104105
Anti-CD29 PE (HMβ1-1)	BioLegend	Cat# 102207
Anti-CD18 PE (M18/2)	BioLegend	Cat# 101407
Anti-CD49 PE (5H10-27 (MFR5)	BioLegend	Cat# 103805
Anti-CD11c PE (N418)	BioLegend	Cat# 117307
**Oligonucleotides and other sequence-based reagents**		
*Tre1* forward primer 5′-ATTAGTGCCTGTGTCTTTGTGAC-3′		Custom-made oligo
*Tre1* reverse primer 5′-GGAGATGCTTAGCGAAATGACG-3′		Custom-made oligo
*mys* forward primer 5′-CGCGGTGCTACCAAAACAC-3′		Custom-made oligo
*mys* reverse primer 5′-GAATCTGCTCAACTGTTATCGGA-3′,		Custom-made oligo
*Fak* forward primer 5′-GCTGACCGATGATTATGC-3′		Custom-made oligo
*Fak* reverse primer 5′-CGAACGGTGGGCGTAGAGTAG-3′,		Custom-made oligo
*Gapdh1* forward primer 5′-TAAATTCGACTCGACTCACGGT-3′		Custom-made oligo
*Gapdh1* reverse primer 5′-CTCCACCACATACTCGGCTC-3′.		Custom-made oligo
*Srp smFISH probe*	Molecular instruments	Custom-made probes
*Gαo smFISH probe*	Molecular instruments	Custom-made probes
X1-488	Molecular instruments	
X3-647	Molecular instruments	
**Chemicals, enzymes, andother reagents**		
Recombinant Mouse TNF-alpha (aa 80–235) Protein	R&D Systems	Cat# 410-MT-010/CF
Recombinant mouse ICAM-1 protein (Active)	Abcam	Cat# ab277758
Mouse CXCL1 (GRO alpha) Recombinant Protein	Gibco (PeproTech®)	Cat# 250-11-20UG
Cell Tracker™ Green CMFDA	Thermo Fisher Scientific	Cat# C7025
Cell Tracker™ Orange CMRA	Thermo Fisher Scientific	Cat# C34551
LY237 (GPR84 agonist)	MedChem Express	Cat# HY-178234
WKYMVm (FPR agonist)	Merk	Cat# 344216-5MG
Neutrophil Isolation Kit	Miltenyi	Cat# 130-097-658
RhoA G-LISA GTPase Activation Assay Kit	Cytoskeleton	Cat# BK124
GLPG1205	MedChem Express (Galapagos Pharmaceuticals)	Cat# HY-135303
RNeasy Mini Kit	Qiagen	Cat# 74104
Maxima First Strand cDNA Synthesis Kit	Thermo Fisher Scientific	Cat# K1651
Double-strand-specific DNase	Thermo Fisher Scientific	Cat# 18068015
HCR Gold RNA-FISH kit (v3.0)	Molecular instruments	
Antifade mounting medium with DAPI	Vector Laboratories	Cat# H-1200-10
Cacodylate	Thermo Fisher Scientific	Cat# A12075.88
PowerUp Syber Green	Thermo Fisher Scientific	Cat# A25742
**Software**		
Imaris 9	Bitplane, Zurich, Switzerland	
FlowJo 10	Treestar	
Huygens 25.10	Scientific Volume Imaging	
NIS version 6.10.02	Nikon	
Prism	Graphpad	
ImageJ/fiji	NIH	
**Other**		
Zeiss LSM800 microscope	Zeiss	Bio-Imaging Facility | CMR (centre-for-microvascular-research.com)
Leica SP8 microscope	Leica	Bio-Imaging Facility | CMR (centre-for-microvascular-research.com)
Leica TCS SP8 AOBS	Leica	Wolfson Bioimaging Facility (https://www.bristol.ac.uk/wolfson-bioimaging/)
CSU-W1 SoRa Spinning Disk microscope	Nikon	Bio-Imaging Facility | CMR (centre-for-microvascular-research.com)
Brightfield microscope	Axioskop FS, Carl Zeiss	
LSR Fortessa flow cytometer	BD Biosciences	Barts Cancer Institute Flow Cytometry Facility | (bci.qmul.ac.uk/core-services/flow-cytometry/)
FACS Aria™ II	BD Biosciences	Barts Cancer Institute Flow Cytometry Facility | (bci.qmul.ac.uk/core-services/flow-cytometry/)
Sunrise microplate reader	Tecan^TM^	

### *Drosophila* stocks and husbandry

Fly stocks were maintained according to standard protocols. All crosses were performed at 25 °C unless otherwise stated. The following *Drosophila* stocks were used: *ubi-GMA, srp-gal4* (Brückner et al, 2004)*, UAS-mcherry, srp-mcherry (gift from Daria Siekhaus)*, *Act-Gal4, UAS-GMA, w;;Tre1-RNAi* P{TRiP.HMS00599} (Bloomington *Drosophila* Stock Center #33718), *Oregon R*, *AniRBD* (a gift from T. Lecuit (Munjal et al, 2015)), *w;Tre1*^*KO*^ (a gift from D.J. Andrew (Kim et al, 2021)), *w;;UAS-mys* (Bloomington *Drosophila* Stock Center #68158), *w;Gαo-RNAi* P{TRiP.hms01129} (Bloomington *Drosophila* Stock Center #34653). Of note, in all studies conducted in *Drosophila*¸ all driver and reporter lines were out-crossed to *Oregon R* (*wild-type*) and the offspring used as a comparative baseline (control) against mutants.

### *Drosophila* dissections, wounding, and imaging

Larvae were sexed, and male pre-pupae were collected 75 h prior to wounding and kept at 25 °C to ensure consistent staging. 75 h after puparium formation (APF), pupae were then placed on double-sided tape ventral side down, and forceps were used to remove the pupa. For imaging, dissected pupae were placed left side down in a glass-bottomed microscopy dish (Greiner Bio-One GmbH, 627861). Wounds were induced using a Micro Point ablation laser (Andor Technology Oxford Instruments) either attached to an Olympus BX53 upright microscope or a Leica SP8 confocal microscope.

### Identifying Tre1 orthologs, multiple sequence alignment, and phylogenetic tree construction

Tre1 paralogues and orthologs in *Drosophila*, mice, and humans were identified using DIOPT, with those sequences having a weighted score >5 selected for further analysis (Hu et al, 2011). *Drosophila* protein sequences were retrieved from FlyBase, and human and mouse protein sequences from the NCBI protein database. The longest isoform was selected if there were multiple isoforms per gene. Multiple sequence alignment was performed with Clustal Omega, and a phylogenetic tree was constructed with JalView. Structural alignment and visualization of orthologous and non-homologous protein structures were performed using UCSF ChimeraX (Pettersen et al, 2021).

### RNA isolation, reverse transcription, and real-time quantitative PCR

RNA was isolated from *WT* and *Tre1*^*KO*^*;;* 75 h APF pupae by crushing in TRIzol (Life Technologies) and RNA purified using an RNeasy Mini Kit (Qiagen). Equal quantities of RNA were then reverse transcribed using a Thermo Scientific Maxima First Strand cDNA Synthesis Kit, and genomic DNA was eliminated using double-strand-specific DNase (Thermo Scientific). Relative quantification of gene expression was performed using SYBR Green Supermix with a real-time PCR machine. Target gene expression was normalized to the expression of the housekeeping reference gene *Gapdh1* using the ΔΔCt analysis method. The following primers were used in this study: *Tre1* F-primer 5′-ATTAGTGCCTGTGTCTTTGTGAC-3′ and R-primer 5′-GGAGATGCTTAGCGAAATGACG-3′, *mys* F-primer 5′-CGCGGTGCTACCAAAACAC-3′ and R-primer 5′-GAATCTGCTCAACTGTTATCGGA-3′, *Fak* F-primer 5′-GCTGACCGATGATTATGC-3′ and R-primer 5′-CGAACGGTGGGCGTAGAGTAG-3′, and *Gapdh1* F-primer 5′-TAAATTCGACTCGACTCACGGT-3′ and R-primer 5′-CTCCACCACATACTCGGCTC-3′.

### Hybridization chain reaction (HCR) on pupae

HCR fluorescence in situ hybridization (HCR-FISH) was performed on whole *Drosophila* 75 h APF pupae using the HCR v3.0 system (Molecular Instruments, USA) and following the protocol of Rose et al (Rose et al, 2022) with minor modifications. Briefly, whole pupae were pierced near the wing hinge to improve permeability of tissue and fixed in 4% formaldehyde for 30 min at RT, followed by methanol series to dehydrate and rehydrate samples. Pupae were then incubated in proteinase K (30 µg/ml) for 30 min at RT, followed by another fixation with 4% formaldehyde to help preserve tissue integrity before proceeding to hybridization. Samples were incubated overnight at 37 °C with HCR panel targeting *Gαo* (X3) and *srp* (X1) (1:50 dilution). Then, the samples were treated for 24 h at RT with HCR hairpin amplifiers X1-488 and X3-647 (1:50 dilution). Finally, samples were mounted in antifade mounting medium containing DAPI (Vector Laboratories, USA) and imaged on a Leica SP8 confocal microscope.

### Correlative light electron microscopy on pupae

For CLEM studies, confocal images were acquired as described, and wing hinges were punctured with forceps before fixation in freshly prepared 2.5% glutaraldehyde (Thermo Fisher Scientific) in 0.1 M cacodylate buffer (Thermo Fisher Scientific) for 24 h at 4 °C. Pupae were then washed in 0.1 M sodium cacodylate (three times for 10 min) before being placed in a secondary fixative containing 1% OsO_4_, 1.5% potassium ferrocyanide, and 0.1 M sodium cacodylate for 2 h at 4 °C. They were again washed in 0.1 M sodium cacodylate (three times for 10 min) and wings dissected and embedded in a solution of 13.5% BSA, 0.1 M cacodylate, and 5% glutaraldehyde for 10 min. Wings were then placed in an automatic tissue processor (Leica EM TP). In all, 70 nm sections were cut with a UC6 ultramicrotome (Leica) with Diamond Knife (Diatome), and were collected onto pioloform-coated copper slot grids (Agar Scientific). These sections were then imaged using a Tecnai 12 BioTwin Spirit TEM (tungsten filament, 120 kV) and a FEI Eagle 4k×4k CCD camera.

### Murine husbandry

All transgenic mice used were on a C57BL/6 N background. *Gpr84*^*−/−*^ mice (and WT littermate controls) were derived from cryopreserved sperm (provided by GlaxoSmithKline) at MRC Harwell. GPR84 (GenBank accession number NM_030720, Ensembl identification number ENSMUSG00000063234) KO mice were provided by Deltagen under a GSK license agreement. As summarized in (Nicol et al, 2015), the insertion of an IRES–LacZ–poly(A) expression cassette replaced 257 bp of coding sequence to generate a premature stop codon, thus deleting in the first three transmembrane domains. Heterozygote breeding pairs were used to maintain the colony at Queen Mary University of London.

For confocal intravital microscopy studies, myeloid cells and pericytes were visualized by generating a *Lyz2-EGFP-ki*; *Acta2-RFPcherry-Tg* colony. The Lyz2-EGFP-ki mice, summarized in (Faust et al, 2000), were crossed with *Acta2-RFPcherry-Tg* mice in which insertion of the RFP variant mCherry under control of the *Acta2* promoter to generate RFP+ pericytes and smooth muscle cells (Proebstl et al, 2012). *Lyz2-EGFP-ki*; *Acta2-RFPcherry-Tg* mice were crossed with *Gpr84*^*+/−*^ (heterozygote) mice across several generations to generate WT and *Gpr84*^*−/−*^ mice on the *Lyz2-EGFP-ki*; *Acta2-RFPcherry-Tg* background.

For the chimeric studies detailed below, *Lyz2-EGFP-ki* mice were used as bone-marrow donors to C57Bl6/J recipient mice with a CD45.1 allelic variant, which were a gift from Dr Suchita Nadkarni (William Harvey Institute, Queen Mary University London).

All animals were used between 8 and 20 weeks old, and all experimental groups were age-matched. For all pharmacological blockade studies, wild-type C57/Bl6J mice were purchased from Charles River. Male mice were used for all cremaster and peritonitis studies; both males and females were used for ear IR and cutaneous wound healing experiments.

All animals were group housed in individually ventilated cages under specific pathogen-free (SPF) conditions and a 12 h light–dark cycle. Animals were humanely sacrificed via cervical dislocation or exsanguination at the end of experiments in accordance with UK Home Office regulations. All in vivo experiments were conducted at the William Harvey Research Institute, Queen Mary University of London, UK, under the UK legislation for animal experimentation and in agreement with the UK Home Office Animals Scientific Procedure Act 1986 (ASPA).

Unless constrained by evident phenotypic alterations (e.g., leukocyte recruitment solely observed in stimulated animals but not in control subjects), all experiments involved steps in which experimenters were blinded. All sample sizes were determined based on previous studies, and the order of GLPG1205 treatment was randomized across batches.

### Brightfield intravital microscopy and analysis

Cremaster muscles were inflamed with an intrascrotal (i.s.) injection of TNF (300 ng) or vehicle control in a 400 µl bolus of PBS. The mice were administered an i.p. injection of ketamine (100 mg/kg) and xylazine (10 mg/kg) 1.5 h prior to exteriorization of the cremaster muscle onto an imaging stage. The cremaster muscles were continuously superfused in warm Tyrode’s solution, and the animals were maintained at 37 °C throughout image acquisition.

Brightfield intravital microscopy was used as a preliminary assessment of real-time leukocyte dynamics. Firmly adhered leukocytes were considered those that remained stationary on the luminal endothelium for ≥30 s. The number of extravasated leukocytes within 50 µm of either side of a 100 µm post-capillary venule segment was quantified at the 4 h experimental end point. Post-capillary venules were observed and movies captured using a ×63 water dipping objective on a transmitted light upright fixed stage microscope (Axioskop FS,Carl Zeiss) with a digital CMOS camera (Hamamatsu).

### Generation of bone marrow chimeric animals

To generate chimeric mice, C57Bl6/J mice (on a CD45.1 allelic background) were irradiated with 4 Gy (3 min 25 s) two times, 4 h apart. The day after irradiation, ∼1.5 × 10^6^ bone marrow-derived cells were administered to each mouse by i.v. injection. Four weeks after irradiation, myeloid cell reconstitution efficiency was assessed by whole blood collection and flow cytometry to calculate absolute leukocyte counts prior to induction of inflammation in the cremaster muscles (all mice showed an effective reconstitution efficiency, as shown in Fig. EV2). After 4 h, the animals were sacrificed, the cremaster muscles were exteriorized and fixed prior to permeabilization and immunofluorescence staining.

### Inflammatory response in the cremaster muscles

Mice were briefly anesthetized with 3% isofluorane and injected i.s. with TNF (300 ng) (R&D Systems) or phosphate-buffered saline (PBS) as a vehicle control, together with fluorescently labeled non-blocking anti-CD31 mAb (4 µg, clone 390, Thermo Fischer Scientific) to label endothelial cell junctions, in a 400 µl bolus using a 2–5 h stimulation period.

### Whole-mount immunofluorescence staining

Exteriorized cremaster or ear tissues were fixed for 1 h in 4% paraformaldehyde (PFA) washed in PBS and permeabilised for 3 or 5 h, respectively, in PBS containing 25% fetal calf serum (FCS) and 0.5% Triton-X. Tissues were stained with fluorescently labeled monoclonal antibodies, MRP-14 (a gift from Professor Nancy Hogg) or PECAM-1, overnight in PBS containing 10% FCS at 4 °C. Antibody conjugation to AF488, 647, or 405 was performed using kits according to the manufacturer’s instructions (ThermoFisher Scientific).

### Confocal microscopy and image analysis

Immunostained whole-mount cremaster muscles or ears were imaged with an upright Leica TCS SP8 (Leica) (with a white light laser (tuneable 470–670 nm)) or an inverted LSM800 (Carl Zeiss) confocal laser scanning microscope equipped with solid-state laser diodes (405, 488, 561, and 640 nm excitation wavelengths), respectively. For high-resolution confocal imaging of neutrophil-vessel wall structures, either the LSM800 (Carl Zeiss) or a CSU-W1 SoRa Spinning Disk microscope (Nikon) was used prior to deconvolution with either Huygens 25.10 (Scientific Volume Imaging) or NIS version 6.10.02 (Nikon), respectively.

For all quantified images, Z-stacks of half post-capillary venules, in a longitudinal orientation, were collected. Post-capillary venules 20–45 µm in diameter were selected for image acquisition, and images were collected at 1024 × 1024 pixels. In cremaster tissues, the number of extravasated neutrophils was quantified per field of view (typically 330 × 160 × 45 µm). For IR injury experiments in the ear (described below), the number of extravasated neutrophils within 50 µm from the vessel wall was quantified.

### Confocal intravital microscopy and analysis

Confocal IVM was used to assess the migration dynamics of neutrophils across the endothelium and interactions with pericytes. WT and *Gpr84*^*−/−*^ mice on a *Lyz2-EGFP-ki*; *Acta2-RFPcherry-Tg* mice, mice were subject to an injection of TNF (300 ng). Movies were acquired on a Leica SP8 laser scanning microscope with a ×20 water-dripping objective (NA 1.0) and 8 kHz resonant scanner. Postcapillary venules between 20 and 45 µm were imaged for 1 h with two venules imaged per mouse 2–5 h post TNF-injection. Half vessels in a longitudinal orientation were recorded to achieve dimensions of typically 300 × 130 × 35 µm with a resulting voxel size of approximately 0.29 (*x*) × 0.29 (*y*) × 0.69 (*z*) µm, and serial z-stacks of 0.7 µm optical sections were acquired every 60 s. The time-lapse serial z stacks were assembled and processed into 3D videos and rendered using IMARIS software^TM^ (Bitplane, Zurich, Switzerland) for analysis.

Neutrophil dynamics were determined by manual tracking of individual neutrophils using IMARIS software. When assessing intraluminal behaviors, neutrophil “crawling” on endothelial cells was defined as a directional movement of ~1 cell radius (~10 µm). Individual cells, which were clearly visible in terms of dynamics and location, were tracked over 3 or more frames (≥ 3 min) to generate the speed of crawling (µm/min). Neutrophil tethers were defined as elongated structures of 5–10 µm in length with a rear contact point on the luminal endothelium. The number of neutrophil-derived fragments within a post-capillary venule, over each 1 h imaging period, was manually counted. Neutrophil fragments were considered as spherical, GFP^+^ structures that were notably smaller than neutrophils with an average radius of approximately ~2 µm.

To assess neutrophil dynamics across the vessel wall, neutrophil TEM was defined as an event where neutrophils fully migrated through EC junctions in a luminal-to-abluminal direction. Trans- pericyte migration (TPM) was defined as the point at which a neutrophil displayed an initial protrusion through the pericyte layer before completing their migration into the perivascular space. Neutrophils were categorized as displaying “aborted” TPM when they displayed a minimum of 1 oscillation during the 1 h imaging period—a protrusion of the leading edge through the pericyte border followed by a retraction into the sub-endothelial space. In Fig. 5B, the maximum length of a perivascular neutrophil was measured within a 1 h imaging period; measurements were taken from the leading edge of the neutrophil to the point of attachment between the posterior protrusion and the venular wall. In Fig. 5C, the time attached to the vessel wall was considered the period between the end of TPM and the completion of tail detachment from the venular wall.

### Transmission electron microscopy

For mouse transmission electron microscopy studies, cremaster muscles were excised from mice subjected to 4 h TNF-induced inflammation, pinned to dental wax before fixation in a freshly prepared mix of 2.5% glutaraldehyde (Thermo Fisher Scientific) in 0.05 M cacodylate buffer (Thermo Fisher Scientific) supplemented with 0.18 mM calcium chloride, for 16 h at 4 °C. Samples were then secondarily fixed in 1% OsO_4_ for 1 h, and taken through serial dehydrations in an automatic tissue processor (Leica EM TP) prior to embedding in resin. 70 nm sections were cut with a UC6 Leica ultramicrotome and Diamond Knife (Diatome) and were collected onto pioloform-coated copper slot grids (Agar Scientific) and imaged using a Tecnai 12 BioTwin Spirit TEM (tungsten filament, 120 kV) and a FEI Eagle 4k×4k CCD camera.

### Whole blood collection

Mice were either terminally anesthetized with 3% isoflurane and the abdomen exposed to collect whole blood from the caudal vena cava, followed by cervical dislocation, or sacrificed prior to a cardiac puncture with collection of whole blood directly into 50 mM EDTA.

### Flow cytometry

Differential leukocyte counts in whole blood and peritoneal lavage were assessed by flow cytometry. Blood samples were incubated with ACK erythrocyte lysis buffer (150 mM NH_3_CL, 1 mM KHCO_3_, and 1 mM EDTA) for 5 min. Samples were then washed once with staining buffer (2 mM EDTA and 0.5% BSA in PBS). Samples were then incubated with Fc-block (anti-CD16/32) antibodies (5 µg/ml) diluted in staining buffer for 15 min at 4 °C. Subsequently samples were stained for surface cells markers diluted in staining buffer (and their appropriate isotypes controls); anti-CD45 PB (1 µg/ml; 30-F11; 103126; BioLegend), CD3 PE (1 µg/ml; 17A2; 100205; BioLegend), CD19 AF488 (1 µg/ml; 6D5; 115524; BioLegend), CD11b PE/Cy7 (0.2 µg/ml; M1/70; 101216; BioLegend), Ly6C PerCP-Cy5.5 (0.2 µg/ml; HK1.4; 128012; BioLegend), Ly6G AF647 (0.5 µg/ml; 1A8; 127610; BioLegend), F4/80 PE (1 µg/ml; BM8; 123110; BioLegend), CD115 APC-Cy7 (1 µg/ml; AFS98; 135531; BioLegend), CD62L PE (0.2 µg/ml; MEL-14;104407; BioLegend), CD11a PE (0.1 µg/ml; M17/4; 101107; BioLegend), CD11b PE (0.1 µg/ml; M17/0; 101207; BioLegend), CD61 PE (0.4 µg/ml; 2C9.G2 (HMβ3-1); 101107; BioLegend), CD51 PE (0.4 µg/ml; RMV-7; 104105; BioLegend), CD29 PE (0.4 µg/ml; HMβ1-1; 102207; BioLegend), CD18 PE (2 µg/ml; M18/2; 101407; BioLegend), CD49 PE (0.4 µg/ml; 5H10-27 (MFR5); 103805; BioLegend), CD11c PE (1 µg/ml; N418; 117307; BioLegend). Gating strategies were based on isotype and FMO controls. Accurate cell counts were validated using counting beads and analyzed using a LSR Fortessa flow cytometer (BD Biosciences) and FloJo software (TreeStar).

### Bulk-RNA sequencing

Neutrophils from the whole blood of male WT versus GPR84 KO mice were FACs sorted and gated using Zombie Yellow™ fixable Viability kit as a live/dead marker, single cells, CD45^+^, CD3^-^, CD19^-^, CD11b^+^, Ly6C^int^, Ly6G^+^ using a FACS Aria™ II (BD Biosciences). Exactly 10,000 neutrophils were collected in Trizol and sent to GENEWIZ (Azenta Life Sciences, US) for RNA isolation, quality control, and ultra-low input RNA sequencing amplification. Sequencing was performed on an Illumina HiSeq instrument with a 2×150-base-pair (BP) paired-end configuration. In total, 35 million reads were mapped to the reference mouse transcriptome (Gencode release v28, GRCm39), and transcripts were quantified by the STAR aligner v2.7.3a in 2-pass mapping mode. Protein-coding and immunoglobulin genes were used for analysis. Counts were subjected to variance-stabilizing transformation (VST) using DESeq2 v.1.42.1. Principal component analysis (PCA) was undertaken with the prcomp function in R using the VST data, and its plots were generated using ggplot2 v3.4.4. Differential expression analysis was performed on count data using DESeq2 v.1..42 using an unadjusted *P* value cut-off of 0.05 to identify transcripts differentially expressed between genotypes, where the volcano plot was visualized using easylabel v0.3.3. Pathway analysis was performed on DE transcripts using the WikiPathways 2024 by EnrichR database (as described by (Kuleshov et al, 2016) with enriched pathways considered significant with an adjusted *P* value of P < 0.05. Heatmaps were generated using ComplexHeatmap v2.21.1.

### In vitro adhesion assay

Ibidi chamber 8-well slides were coated overnight with 3 µg/ml rmICAM-1 (Abcam) at 4 °C, blocked with 1% Casein (in PBS) for 1 h at room temperature (RT), and washed with PBS. Murine neutrophils were isolated from bone marrow cells (harvested from tibiae and femora) by negative magnetic cell sorting using the Neutrophil Isolation Kit (Miltenyi Biotec) according to the manufacturer’s instructions. The purity of isolated neutrophils (CD45^+^/Ly6G^+^) expression profile) was consistently > 95% as determined by flow cytometry. Isolated neutrophils were stained with Cell Tracker™ Green CMFDA or Cell Tracker™ Orange CMRA (Thermofisher Scientific) at 0.5 µM for 10 min at RT. Potential effects of Cell Tracker™ colors were controlled for by alternating between genotypes for experimental replicates. Isolated neutrophils were seeded at 100,000 of each genotype and left to settle for ~20 min at RT prior to stimulation with 10 nM CXCL1 (Peprotech) for 15 min at 37 °C and imaged on a Nikon Spinning Disk SoRa at 60x magnification prior to assessment of the proportion of “spread” or motile cells by manual counting on IMARIS software. Neutrophils were classified as “spread” when displaying a flattened and non-spherical-like structure and considered as “motile” when displaying membrane ruffling and migration on the ICAM-1 coated surface. To assess cell surface area, images at the 15 min endpoint were analyzed using ImageJ (NIH).

### RhoA G-LISA GTPase activation assay

Murine bone marrow-derived neutrophils from WT and *Gpr84*^*−/−*^ mice were isolated as detailed above. At least 3 million cells (pooled from 2 to 3 mice) per sample were stimulated with either the GPR84 agonist LY237 (10 µM) (MedChem Express), or as a positive control, with the FPR agonist WKYMVm (1 µM), for 4 min at 37 °C. Cells were lysed at 4°, the protein content collected, and immediately snap-frozen. Samples were normalized for protein content and processed using RhoA G-LISA GTPase Activation Assay Kit (Cytoskeleton) according to the manufacturer’s instructions. Biological replicates were performed, and all samples were run in technical triplicates with absorbance read at 490 nm using a Sunrise microplate reader (Tecan^TM^).

### Zymosan-induced peritonitis

C57Bl6/J mice (Charles River) received 10 or 30 mg/kg GLPG1205 (Galapagos Pharmaceuticals) in (40% PEG 200, 60% sterile water) or vehicle control by oral gavage 24 h prior to induction of inflammation in the TNF-inflamed cremaster model or zymosan-induced peritonitis. Peritonitis was induced by an i.p. injection of zymosan (1 mg) in 500 µl of PBS or PBS alone as a control. Subsequently, 24 h later, the peritoneal lavage was collected in 5 ml PBS containing 5 mM EDTA (Sigma-Aldrich) and 0.25% BSA. The peritoneal exudates were labeled for myeloid cell markers of interest for flow cytometry and gated as follows for neutrophils (single cells, live, CD45^+^ CD115^−^ F4/80^−^ Ly6C^int^ Ly6G^+^) and monocytes (single cells, live, CD45^+^ CD115^+^ F4/80^low-int^ Ly6C^hi-int^ Ly6G^-^). Absolute cell counts were calculated with the application of counting beads and assessed by flow cytometry.

### Ischemia–reperfusion injury in the ear

Male and female C57Bl6/J mice (Charles River) received 30 mg/kg GLPG1205 or vehicle control as above. Mice were briefly anesthetized with 3% isoflurane prior to application of magnets covering the lower region of the ear; anesthesia was reduced to 1.5% during the 1.5 h ischemia period, and a heating pad was used to maintain body temperature at 37 °C. The magnets (2 mm thick 45H Neodymium with 0.8 kg pull) were removed prior to a 3 h reperfusion period. Mice were sacrificed, whole blood collected (to assess differential leukocyte counts), and ear tissues harvested and processed for staining and analysis.

### Cutaneous wound injury

Male and female C57Bl6/J mice (Charles River) received 30 mg/kg GLPG1205 or vehicle control as above. Briefly, 8-week-old mice were anesthetized with 3% isofluorane, prior to shaving their back skin and generating paired excisional wounds with a 4 mm biopsy punch (Kai Medical). Animals were sacrificed at 0, 18, and 36 h post-wounding, and skin samples were processed for histological analysis.

### Histological staining and image analysis

Tissue was fixed in 4% paraformaldehyde overnight prior to transfer through serial dehydration steps in preparation for embedding in paraffin using an automatic tissue processor (Expredia Exelsior AS). In all, 7-μm sections were cut with a Leica 2125 microtome and subsequently deparaffinized and stained with hematoxylin and eosin. Stained sections were then imaged in an Olympus VS200 slide scanner, followed by analysis in QuPath (Bankhead et al, 2017). Quantification of the total number of neutrophils was performed using the built-in Cell Detection algorithm that detects hematoxylin-stained nuclei with minor adjustments to intensity parameters for neutrophil nuclear morphology: threshold (0.15) and cell expansion (2 µm). This was validated by comparing manual and automated counts.

### Quantification and statistics

Statistical analyses were performed using Prism 10.1 software (GraphPad), and all datasets are expressed as mean ± SEM. Details for exact n numbers are reported in the legends for each Figure. Sample sizes were chosen based on published work in which similar results have been reported.

## Supplementary information

Peer Review File

Movie EV1

Movie EV2

Movie EV3

Movie EV4

Movie EV5

Movie EV6

Movie EV7

Source data Fig. 1

Source data Fig. 2

Source data Fig. 3

Source data Fig. 4

Source data Fig. 5

Source data Fig. 7

Source data Fig. 8

Source data Fig. 9

Expanded View Figures

## Data Availability

RNA-Seq data are available at ArrayExpress (with accession ID E-MTAB-16278). The following access code provides a direct link to this dataset: https://www.ebi.ac.uk/biostudies/arrayexpress/studies/E-MTAB-16278?key=3e828e38-79a7-45fa-a8be-27e824be2973. The source data of this paper are collected in the following database record: biostudies:S-SCDT-10_1038-S44319-026-00923-0.

## References

[CR1] Abdel-Aziz H, Schneider M, Neuhuber W, Kassem AM, Khailah S, Müller J, Eldeen HG, Khairy A, Khayyal MT, Shcherbakova A et al (2016) GPR84 and TREM-1 signaling contribute to the pathogenesis of reflux esophagitis. Mol Med 21:1011–1024 10.2119/molmed.2015.00098 PMC4982478 26650186

[CR2] Abramson J, Adler J, Dunger J, Evans R, Green T, Pritzel A, Ronneberger O, Willmore L, Ballard AJ, Bambrick J et al (2024) Accurate structure prediction of biomolecular interactions with AlphaFold 3. Nature 630:493–500 10.1038/s41586-024-07487-w PMC11168924 38718835

[CR3] Aepfelbacher M, Essler M, Huber E, Czech A, Weber PC (1996) Rho is a negative regulator of human monocyte spreading. J Immunol 157:5070–5075 8943416

[CR4] Bankhead P, Loughrey MB, Fernández JA, Dombrowski Y, McArt DG, Dunne PD, McQuaid S, Gray RT, Murray LJ, Coleman HG et al (2017) QuPath: open source software for digital pathology image analysis. Sci Rep 7:1–7 10.1038/s41598-017-17204-5 PMC5715110 29203879

[CR5] Bouchard C, Pagé J, Bédard A, Tremblay P, Vallières L (2007) G protein-coupled receptor 84, a microglia-associated protein expressed in neuroinflammatory conditions. Glia 55:790–800 10.1002/glia.20506 17390309

[CR6] Brückner K, Kockel L, Duchek P, Luque CM, Rørth P, Perrimon N (2004) The PDGF/VEGF receptor controls blood cell survival in Drosophila. Dev Cell 7:73–84 10.1016/j.devcel.2004.06.007 15239955

[CR7] Chastney MR, Kaivola J, Leppänen VM, Ivaska J (2025) The role and regulation of integrins in cell migration and invasion. Nat Rev Mol Cell Biol 26:147–167 10.1038/s41580-024-00777-1 39349749

[CR8] Chen J, Stork T, Kang Y, Nardone KAM, Auer F, Farrell RJ, Jay TR, Heo D, Sheehan A, Paton C et al (2024) Astrocyte growth is driven by the Tre1/S1pr1 phospholipid-binding G protein-coupled receptor. Neuron 112:93–112.e10 10.1016/j.neuron.2023.11.008 PMC11073822 38096817

[CR9] Chen R, Grill S, Lin B, Saiduddin M, Lehmann R (2025) Origin and establishment of the germline in Drosophila melanogaster. Genetics 229:217 10.1093/genetics/iyae217 PMC12005264 40180587

[CR10] Cooper PO, Kleb SS, Noonepalle SK, Amuso VM, Varshney R, Rudolph MC, Dhaliwal TK, Nguyen DV, Mazumder MF, Babirye NS et al (2024) G-protein-coupled receptor 84 regulates acute inflammation in normal and diabetic skin wounds. Cell Rep 43:114288 10.1016/j.celrep.2024.114288 PMC11247419 38814782

[CR11] Faust N, Varas F, Kelly LM, Heck S, Graf T (2000) Insertion of enhanced green fluorescent protein into the lysozyme gene creates mice with green fluorescent granulocytes and macrophages. Blood 96:719–726 10887140

[CR12] Foulon M, Fraga AG, Robbe-Saule M, Tarnaud F, Lafont M, Lebon G, Fevereiro J, Pignon P, Boneca IG, D’Andon MF et al (2026) The orphan receptor GPR84 drives inflammation in Buruli ulcer development. Sci Transl Med 18:eads8681 10.1126/scitranslmed.ads8681 41984933

[CR13] Gagnon L, Leduc M, Thibodeau JF, Zhang MZ, Grouix B, Sarra-Bournet F, Gagnon W, Hince K, Tremblay M, Geerts L et al (2018) A newly discovered antifibrotic pathway regulated by two fatty acid receptors: GPR40 and GPR84. Am J Pathol 188:1132–1148 10.1016/j.ajpath.2018.01.009 29454750

[CR14] Girbl T, Lenn T, Perez L, Rolas L, Barkaway A, Thiriot A, del Fresno C, Lynam E, Hub E, Thelen M et al (2018) Distinct compartmentalization of the chemokines CXCL1 and CXCL2 and the atypical receptor ACKR1 determine discrete stages of neutrophil diapedesis. Immunity 49:1062–1076.e6 10.1016/j.immuni.2018.09.018 PMC6303217 30446388

[CR15] Hildebrand D, Uhle F, Sahin D, Krauser U, Weigand MA, Heeg K (2018) The interplay of notch signaling and STAT3 in TLR-activated human primary monocytes. Front Cell Infect Microbiol 8:241 10.3389/fcimb.2018.00241 PMC6048282 30042932

[CR16] Hind LE, Vincent WJB, Huttenlocher A (2016) Leading from the back: the role of the uropod in neutrophil polarization and migration. Dev Cell 38:161–169 10.1016/j.devcel.2016.06.031 PMC4982870 27459068

[CR17] Hou Y, Khatri P, Rindy J, Schultz Z, Gao A, Chen Z, Gibson ALF, Huttenlocher A, Dinh HQ (2024) Single-cell transcriptional landscape of temporal neutrophil response to burn wound in larval zebrafish. J Immunol 213:469–480 10.4049/jimmunol.2400149 PMC11300161 38922186

[CR18] Hu X, Chung AY, Wu I, Foldi J, Chen J, Ji JD, Tateya T, Kang YJ, Han J, Gessler M et al (2008) Integrated regulation of Toll-like receptor responses by notch and interferon-γ pathways. Immunity 29:691–703 10.1016/j.immuni.2008.08.016 PMC2585039 18976936

[CR19] Hu Y, Flockhart I, Vinayagam A, Bergwitz C, Berger B, Perrimon N, Mohr SE (2011) An integrative approach to ortholog prediction for disease-focused and other functional studies. BMC Bioinforma 12:1–16 10.1186/1471-2105-12-357 PMC3179972 21880147

[CR20] Hyun YM, Sumagin R, Sarangi PP, Lomakina E, Overstreet MG, Baker CM, Fowell DJ, Waugh RE, Sarelius IH, Kim M (2012) Uropod elongation is a common final step in leukocyte extravasation through inflamed vessels. J Exp Med 209:1349–1362 10.1084/jem.20111426 PMC3405502 22711877

[CR21] Joulia R, Guerrero-Fonseca IM, Girbl T, Coates JA, Stein M, Vázquez-Martínez L, Lynam E, Whiteford J, Schnoor M, Voehringer D et al (2022) Neutrophil breaching of the blood vessel pericyte layer during diapedesis requires mast cell-derived IL-17A. Nat Commun 13:7029 10.1038/s41467-022-34695-7 PMC9672103 36396641

[CR22] Jumper J, Evans R, Pritzel A, Green T, Figurnov M, Ronneberger O, Tunyasuvunakool K, Bates R, Žídek A, Potapenko A et al (2021) Highly accurate protein structure prediction with AlphaFold. Nature 596:583–589 10.1038/s41586-021-03819-2 PMC8371605 34265844

[CR23] Kim JH, Hanlon CD, Vohra S, Devreotes PN, Andrew DJ (2021) Hedgehog signaling and Tre1 regulate actin dynamics through PI(4,5)P2 to direct migration of Drosophila embryonic germ cells. Cell Rep 34:108799 10.1016/j.celrep.2021.108799 PMC8023404 33657369

[CR24] Kowanko IC, Ferrante A (1987) Interleukin 2 inhibits migration and stimulates respiratory burst and degranulation of human neutrophils in vitro. Immunol Lett 15:285–289 10.1016/0165-2478(87)90129-5 3500909

[CR25] Kuleshov MV, Jones MR, Rouillard AD, Fernandez NF, Duan Q, Wang Z, Koplev S, Jenkins SL, Jagodnik KM, Lachmann A et al (2016) Enrichr: a comprehensive gene set enrichment analysis web server 2016 update. Nucleic Acids Res 44:W90–W97 10.1093/nar/gkw377 PMC4987924 27141961

[CR26] LeBlanc MG, Lehmann R (2017) Domain-specific control of germ cell polarity and migration by multifunction Tre1 GPCR. J Cell Biol 216:2945–2658 10.1083/jcb.201612053 PMC5584160 28687666

[CR27] Ley K, Laudanna C, Cybulsky MI, Nourshargh S (2007) Getting to the site of inflammation: the leukocyte adhesion cascade updated. Nat Rev Immunol 7:678–689 10.1038/nri2156 17717539

[CR28] Li J, Gyorffy S, Lee S, Kwok CS (1996) Effect of recombinant human interleukin 2 on neutrophil adherence to endothelial cells in vitro. Inflammation 20:361–372 10.1007/BF01486739 8872500

[CR29] Lin B, Luo J, Lehmann R (2022) An AMPK phosphoregulated RhoGEF feedback loop tunes cortical flow-driven amoeboid migration in vivo. Sci Adv 8:323 10.1126/sciadv.abo0323 PMC9473612 36103538

[CR30] Mancini SJ, Al Mahmud Z, Jenkins L, Bolognini D, Newman R, Barnes M, Edye ME, McMahon SB, Tobin AB, Milligan G (2019) On-target and off-target effects of novel orthosteric and allosteric activators of GPR84. Sci Rep 9:1–15 10.1038/s41598-019-38539-1 PMC6372602 30755705

[CR31] Mosaddeghzadeh N, Ahmadian MR (2021) The rho family gtpases: Mechanisms of regulation and signaling. Cells 10:1831 10.3390/cells10071831 PMC8305018 34359999

[CR32] Muller WA (2016) Transendothelial migration: unifying principles from the endothelial perspective. Immunol Rev 273:61–75. 10.1111/imr.12443 PMC5090979 27558328

[CR33] Munjal A, Philippe JM, Munro E, Lecuit T (2015) A self-organized biomechanical network drives shape changes during tissue morphogenesis. Nature 524:351–355 10.1038/nature14603 26214737

[CR34] Nagasaki H, Kondo T, Fuchigami M, Hashimoto H, Sugimura Y, Ozaki N, Arima H, Ota A, Oiso Y, Hamada Y (2012) Inflammatory changes in adipose tissue enhance expression of GPR84, a medium-chain fatty acid receptor. FEBS Lett 586:368–372 10.1016/j.febslet.2012.01.001 22245676

[CR35] Nicol LSC, Dawes JM, La Russa F, Didangelos A, Clark AK, Gentry C, Grist J, Davies JB, Malcangio M, McMahon SB (2015) The role of G-protein receptor 84 in experimental neuropathic pain. J Neurosci 35:8959–8969 10.1523/JNEUROSCI.3558-14.2015 PMC4461694 26063927

[CR36] Nourshargh S, Alon R (2014) Leukocyte migration into inflamed tissues. Immunity 41:694–707 10.1016/j.immuni.2014.10.008 25517612

[CR37] Nourshargh S, Hordijk PL, Sixt M (2010) Breaching multiple barriers: leukocyte motility through venular walls and the interstitium. Nat Rev Mol Cell Biol 11:366–378 10.1038/nrm2889 20414258

[CR38] Pettersen EF, Goddard TD, Huang CC, Meng EC, Couch GS, Croll TI, Morris JH, Ferrin TE (2021) UCSF ChimeraX: structure visualization for researchers, educators, and developers. Protein Sci 30:70–82 10.1002/pro.3943 PMC7737788 32881101

[CR39] Phillipson M, Heit B, Colarusso P, Liu L, Ballantyne CM, Kubes P (2006) Intraluminal crawling of neutrophils to emigration sites: a molecularly distinct process from adhesion in the recruitment cascade. J Exp Med 203:2569 10.1084/jem.20060925 PMC2118150 17116736

[CR40] Proebstl D, Voisin M-B, Woodfin A, Whiteford J, D’Acquisto F, Jones GE, Rowe D, Nourshargh S (2012) Pericytes support neutrophil subendothelial cell crawling and breaching of venular walls in vivo. J Exp Med 209:1219–1234 10.1084/jem.20111622 PMC3371725 22615129

[CR41] Puengel T, De Vos S, Hundertmark J, Kohlhepp M, Guldiken N, Pujuguet P, Auberval M, Marsais F, Shoji KF, Saniere L et al (2020) The medium-chain fatty acid receptor GPR84 mediates myeloid cell infiltration promoting steatohepatitis and fibrosis. J Clin Med 9:1140 10.3390/jcm9041140 PMC7231190 32316235

[CR42] Razzell W, Wood W, Martin P (2011) Swatting flies: modelling wound healing and inflammation in Drosophila. Dis Model Mech 4:569–574 10.1242/dmm.006825 PMC3180219 21810906

[CR43] Recio C, Lucy D, Purvis GSD, Iveson P, Zeboudj L, Iqbal AJ, Lin D, O’Callaghan C, Davison L, Griesbach E et al (2018) Activation of the immune-metabolic receptor GPR84 enhances inflammation and phagocytosis in macrophages. Front Immunol 9:1419 10.3389/fimmu.2018.01419 PMC6019444 29973940

[CR44] Rose M, Domsch K, Bartle-Schultheis J, Reim I, Schaub C (2022) Twist regulates Yorkie activity to guide lineage reprogramming of syncytial alary muscles. Cell Rep 38:110295 10.1016/j.celrep.2022.110295 35081347

[CR45] Schroeder T, Just U (2000) Notch signalling via RBP-J promotes myeloid differentiation. EMBO J 19:2558 10.1093/emboj/19.11.2558 PMC212745 10835354

[CR46] Schwabe T, Bainton RJ, Fetter RD, Heberlein U, Gaul U (2005) GPCR signaling is required for blood-brain barrier formation in Drosophila. Cell 123:133–144 10.1016/j.cell.2005.08.037 16213218

[CR47] Shin YJ, Kim EH, Roy A, Kim JH (2013) Evidence for a novel mechanism of the PAK1 interaction with the Rho-GTPases Cdc42 and Rac. PLoS ONE 8:e71495 10.1371/journal.pone.0071495 PMC3731272 23936510

[CR48] Simard JC, Thibodeau JF, Leduc M, Tremblay M, Laverdure A, Sarra-Bournet F, Gagnon W, Ouboudinar J, Gervais L, Felton A et al (2020) Fatty acid mimetic PBI-4547 restores metabolic homeostasis via GPR84 in mice with non-alcoholic fatty liver disease. Sci Rep 10:12778 10.1038/s41598-020-69675-8 PMC7391726 32728158

[CR49] Smyth I, Du X, Taylor MS, Justice MJ, Beutler B, Jackson IJ (2004) The extracellular matrix gene Frem1 is essential for the normal adhesion of the embryonic epidermis. Proc Natl Acad Sci USA 101:13560–13565 10.1073/pnas.0402760101 PMC518794 15345741

[CR50] Song Z, Clemens RA, Zhang Y, Chen J, Wang Y, Dinauer MC, Meng S (2025) Investigating pulmonary neutrophil responses to inflammation in mice via flow cytometry. J Leukoc Biol 117:qiae189 10.1093/jleuko/qiae189 PMC12631735 39212489

[CR51] Sun L, Ye RD (2012) Role of G protein-coupled receptors in inflammation. Acta Pharm Sin 33:342–350 10.1038/aps.2011.200 PMC4085652 22367283

[CR52] Suzuki M, Takaishi S, Nagasaki M, Onozawa Y, Iino I, Maeda H, Komai T, Oda T (2013) Medium-chain fatty acid-sensing receptor, GPR84, is a proinflammatory receptor. J Biol Chem 288:10684–10691 10.1074/jbc.M112.420042 PMC3624448 23449982

[CR53] Thuma L, Carter D, Weavers H, Martin P (2018) *Drosophila* immune cells extravasate from vessels to wounds using Tre1 GPCR and Rho signaling. J Cell Biol 217:3045–3056 10.1083/jcb.201801013 PMC6122984 29941473

[CR54] Vestweber D (2015) How leukocytes cross the vascular endothelium. Nat Rev Immunol 15:692–704. 10.1038/nri3908 26471775

[CR55] Wang J, Wu X, Simonavicius N, Tian H, Ling L (2006) Medium-chain fatty acids as ligands for orphan G protein-coupled receptor GPR84. J Biol Chem 281:34457–34464 10.1074/jbc.M608019200 16966319

[CR56] Wang JX, Bair AM, King SL, Shnayder R, Huang YF, Shieh CC, Soberman RJ, Fuhlbrigge RC, Nigrovic PA (2012) Ly6G ligation blocks recruitment of neutrophils via a β2-integrin–dependent mechanism. Blood 120:1489 10.1182/blood-2012-01-404046 PMC3423786 22661700

[CR57] Wang SW, Zhang Q, Lu D, Fang YC, Yan XC, Chen J, Xia ZK, Yuan QT, Chen LH, Zhang YM et al (2023) GPR84 regulates pulmonary inflammation by modulating neutrophil functions. Acta Pharm Sin 44:1665–1675 10.1038/s41401-023-01080-z PMC10072043 37016043

[CR58] Wittenberger T, Schaller HC, Hellebrand S (2001) An expressed sequence tag (EST) data mining strategy succeeding in the discovery of new G-protein coupled receptors. J Mol Biol 307:799–813 10.1006/jmbi.2001.4520 11273702

[CR59] Woodfin A, Voisin M-B, Beyrau M, Colom B, Caille D, Diapouli F-M, Nash GB, Chavakis T, Albelda SM, Rainger GE et al (2011) The junctional adhesion molecule JAM-C regulates polarized transendothelial migration of neutrophils in vivo. Nat Immunol 12:761–769 10.1038/ni.2062 PMC3145149 21706006

[CR60] Worthylake RA, Lemoine S, Watson JM, Burridge K (2001) RhoA is required for monocyte tail retraction during transendothelial migration. J Cell Biol 154:147–160 10.1083/jcb.200103048 PMC2196864 11448997

[CR61] Xu W, Wang P, Petri B, Zhang Y, Tang W, Sun L, Kress H, Mann T, Shi Y, Kubes P et al (2010) Integrin-induced PIP5K1C kinase polarization regulates neutrophil polarization, directionality, and in vivo infiltration. Immunity 33:340–350 10.1016/j.immuni.2010.08.015 PMC2947797 20850356

[CR62] Yin C, Cheng L, Pan J, Chen L, Xue Q, Qin J, Wang S, Du B, Liu M, Zhang Y et al (2020) Regulatory role of Gpr84 in the switch of alveolar macrophages from CD11blo to CD11bhi status during lung injury process. Mucosal Immunol 13:892–907 10.1038/s41385-020-0321-7 32719411

[CR63] Yoshiura S, Ohta N, Matsuzaki F (2012) Tre1 GPCR signaling orients stem cell divisions in the Drosophila central nervous system. Dev Cell 22:79–91 10.1016/j.devcel.2011.10.027 22178499

[CR64] Yousefi S, Cooper PR, Potter SL, Mueck B, Jarai G (2001) Cloning and expression analysis of a novel G-protein-coupled receptor selectively expressed on granulocytes. J Leukoc Biol 69:1045–1052 11404393

[CR65] Zhang Q, Chen LH, Yang H, Fang YC, Wang SW, Wang M, Yuan QT, Wu W, Zhang YM, Liu ZJ et al (2022) GPR84 signaling promotes intestinal mucosal inflammation via enhancing NLRP3 inflammasome activation in macrophages. Acta Pharm Sin 43:2042–2054 10.1038/s41401-021-00825-y PMC9343429 34912006

[CR66] Zhao ZS, Manser E (2005) PAK and other Rho-associated kinases–effectors with surprisingly diverse mechanisms of regulation. Biochem J 386:201–214 10.1042/BJ20041638 PMC1134783 15548136

