# Supplementary material for: Orphan GPR84 facilitates uropod de-adhesion at multiple steps during leukocyte extravasation
Source: EMBO Rep. 2026 Sep 12;27(20):6018–49. doi: 10.1038/s44319-026-00923-0 (PMC13694715; doi:10.1038/s44319-026-00923-0)
Supplement: Supplementary file 1 [file 44319_2026_923_MOESM17_ESM.pdf]

Expanded View Figures

Figure EV1. Tre1 is required by hemocytes to complete extravasation.

(**A**) Quantitative RT-PCR of the relative expression of *Tre1* in 75 h APF whole-pupae in *Oregon R* (*WT*) background versus pupae with RNAi-mediated silencing of *Tre1* ($Tre1^{KD}$) in hemocytes (*srp-Gal4*-driven knockdown) or in fully *Tre1* null pupae ($P = 0.0051$ and $P < 0.0001$, respectively). *Tre1* threshold cycle values were normalized to *Gapdh1*. RT-PCR data represented in a bar plot (mean ± SEM) were overlaid with average values (white dots) for each biological replicate (RNA pooled from 10 animals). Data was analyzed by one-way ANOVA with a Dunnett's test for multiple comparisons. (**B**, **C**) Representative in vivo time-lapse imaging of 75 h APF with $Tre1^{KD}$ hemocytes, which results in a significant reduction in hemocyte extravasation capacity as compared to *WT*. (**D**) Representative in vivo time-lapse imaging of 75 h APF *Tre1* null pupae expressing *Tre1* specifically in hemocytes (*srp-Gal4*-driven expression), which partially recovered the Tre1 extravasation phenotype. Solid white line indicates the border of the wound, white arrows indicate the direction towards the wound that hemocytes extravasated towards, and asterisks indicate hemocytes that have either fully or partially extravasated. (**E**) Comparison of the proportion of intraveinally adhered hemocytes between *WT* and $Tre1^{KD}$ ($P = 0.0054$) or $Tre1^{KO}$ ($P = 0.0007$) genotypes. Hemocytes were classified as adhered if they remained in the same location between consecutive frames (remained still for five minutes). Between six and nine animals were analyzed for each genotype, and data represent mean ± SEM. Data were analyzed by one-way ANOVA with a Tukey's test for multiple comparisons between genotypes. (**F**) Quantification of the number of hemocytes attempting to extravasate at various timepoints after wounding. Six animals were analyzed for each genotype, and data represent mean ± SEM. A two-way ANOVA was performed followed by Tukey's multiple comparisons which determined no significant differences between genotypes at any time point. $^*P < 0.05$, $^{**}P < 0.01$, $^{***}P < 0.001$, $^{****}P < 0.0001$. (**C**, **D**) Yellow asterisks indicate hemocytes, and white arrows show the direction of travel towards wounds. Scale bars: (**B**–**D**) = 15 μm.

© The Author(s)

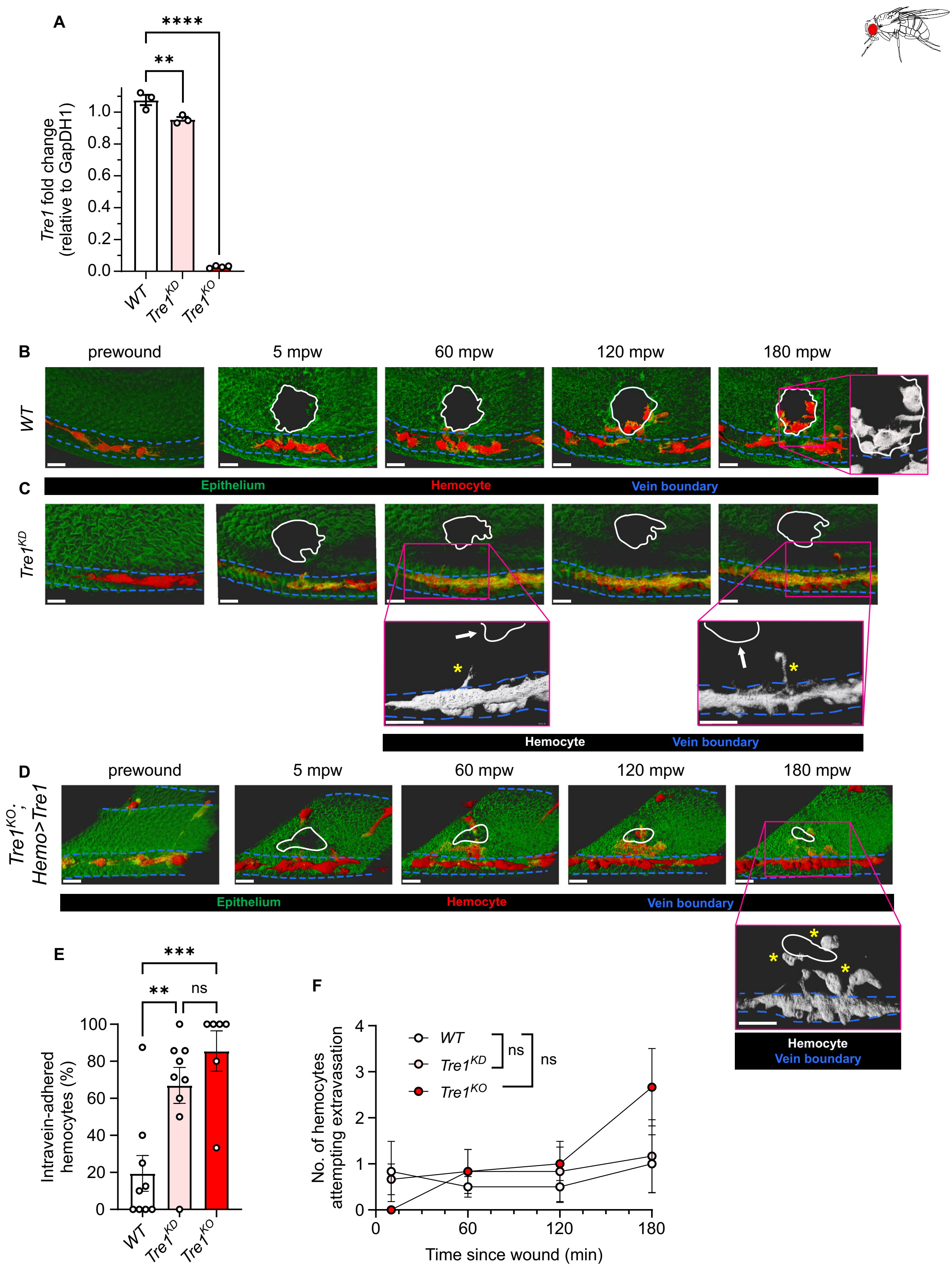
A
****
**
Tre1 fold change (relative to GapDH1)
1.0
0.8
0.6
0.4
0.2
0.0
WT
Tre1KD
Tre1KO
B
prewound
5 mpw
60 mpw
120 mpw
180 mpw
WT
Epithelium
Hemocyte
Vein boundary
C
Tre1KD
Hemocyte
Vein boundary
D
Tre1KO; Hemo>Tre1
prewound
5 mpw
60 mpw
120 mpw
180 mpw
Epithelium
Hemocyte
Vein boundary
Hemocyte
Vein boundary
E
***
**
ns
Intravein-adhered hemocytes (%)
100
80
60
40
20
0
WT
Tre1KD
Tre1KO
F
No. of hemocytes attempting extravasation
WT
Tre1KD
Tre1KO
ns
ns
4
3
2
1
0
0
60
120
180
Time since wound (min)

© The Author(s)

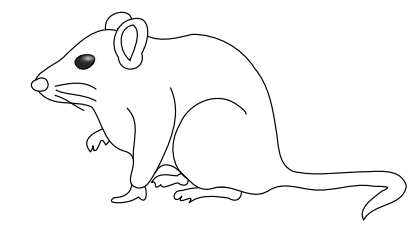

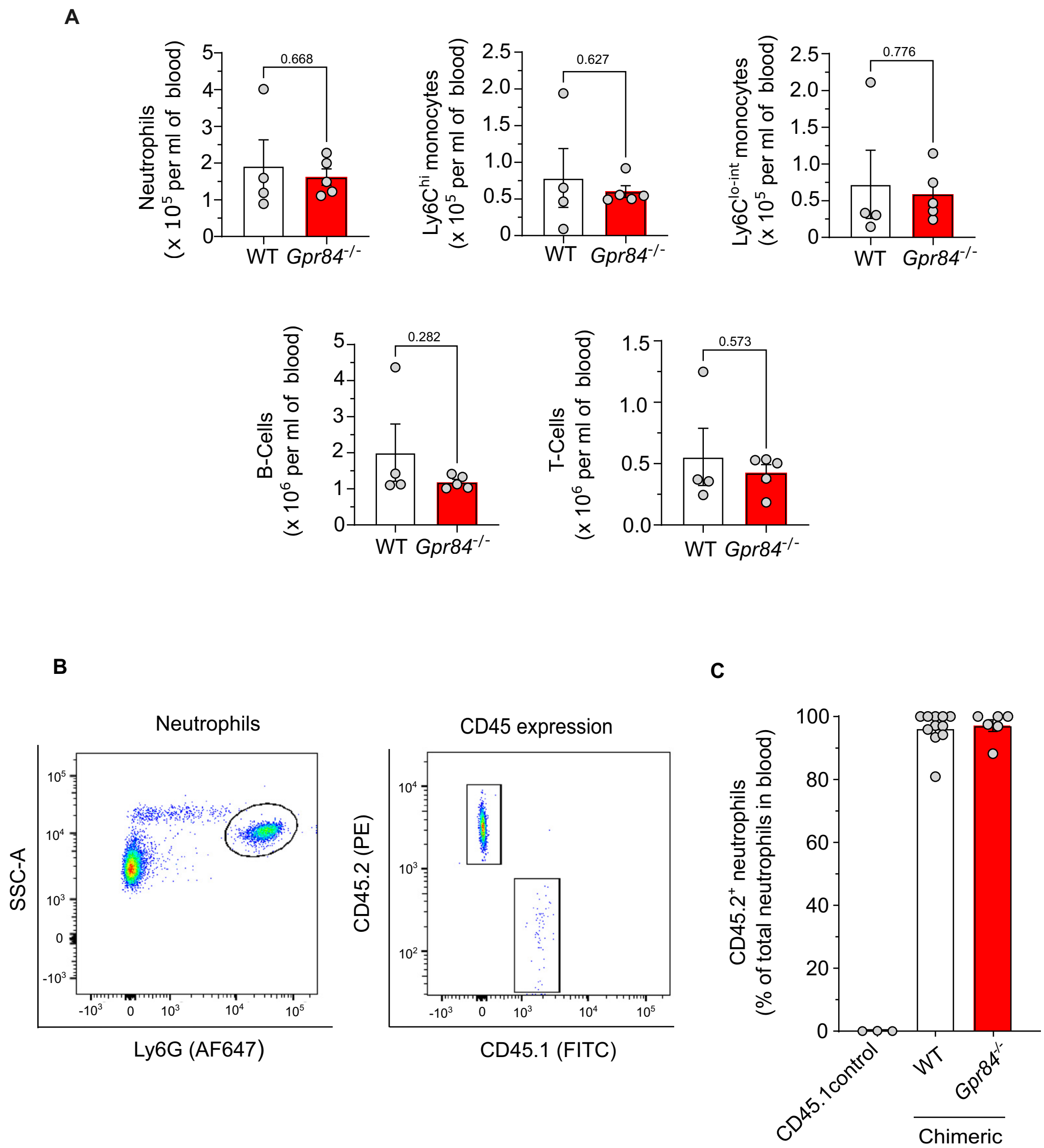

Figure EV2. Blood leukocyte counts of WT vs *Gpr84$^{-/-}$* global knockouts and reconstitution efficiency in chimeric mice after bone marrow transfer.

(**A**) Differential leukocyte counts of whole blood from WT ($n = 4$) and *Gpr84$^{-/-}$* ($n = 6$) mice determined by flow cytometry. (**B**) Representative flow cytometry gating strategy for selecting neutrophils, and (**C**) assessing CD45.2 allelic variant expression as a marker of reconstitution efficiency when generating WT and *Gpr84$^{-/-}$* chimeric mice. Data represent mean ± SEM and in (**A**) were analyzed by an unpaired *T* test with no significance between experimental groups.

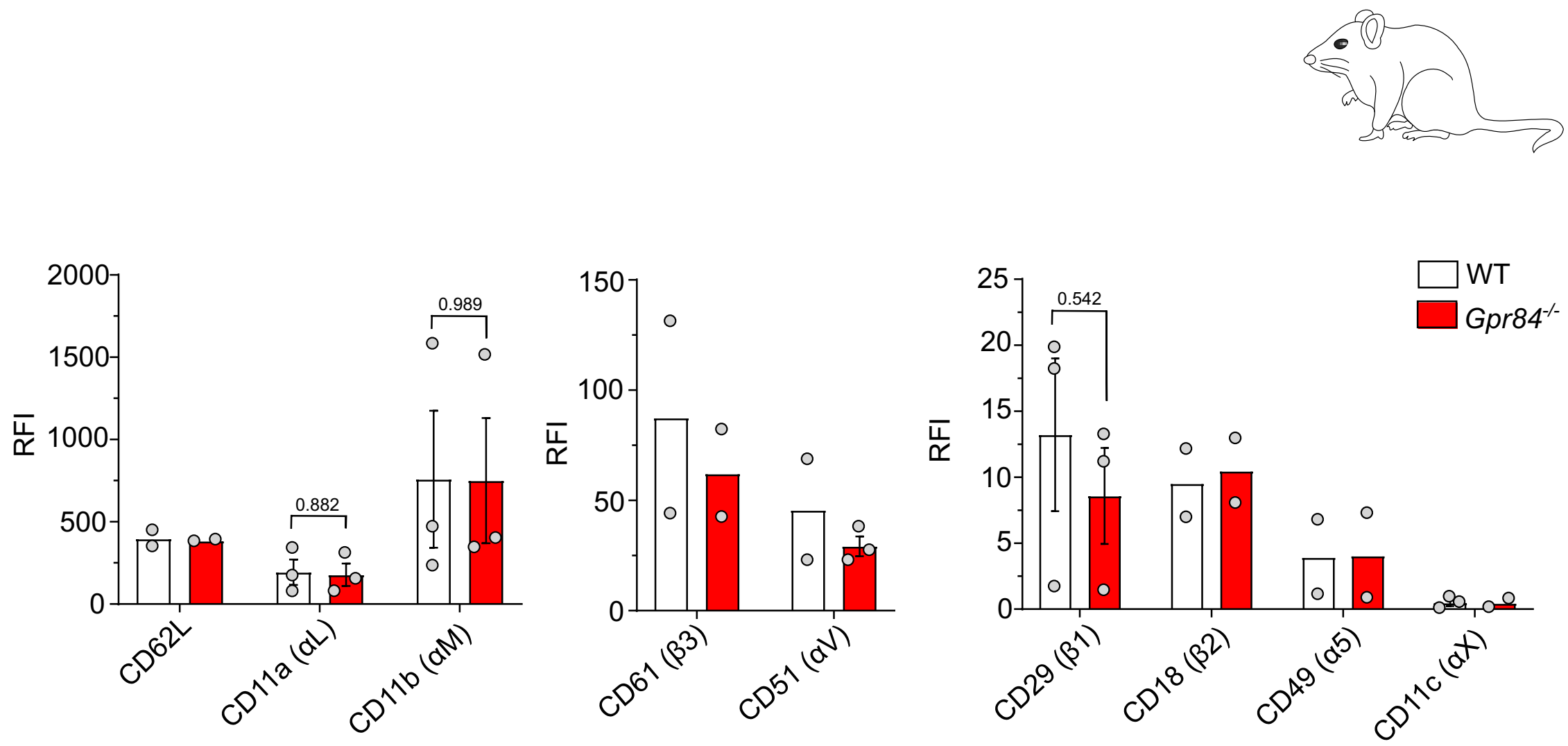

Figure EV3. Integrin expression on WT and *Gpr84*$^{-/-}$ mouse blood neutrophils.

Relative fluorescence intensity (RFI) of surface expression levels of indicated integrins on mouse blood neutrophils, from WT and *Gpr84*$^{-/-}$ as determined by flow cytometry (2–3 mice/group). Data represent mean ± SEM. Data were analyzed by unpaired *T* test with Welch's correction between genotypes where applicable.

 © The Author(s)

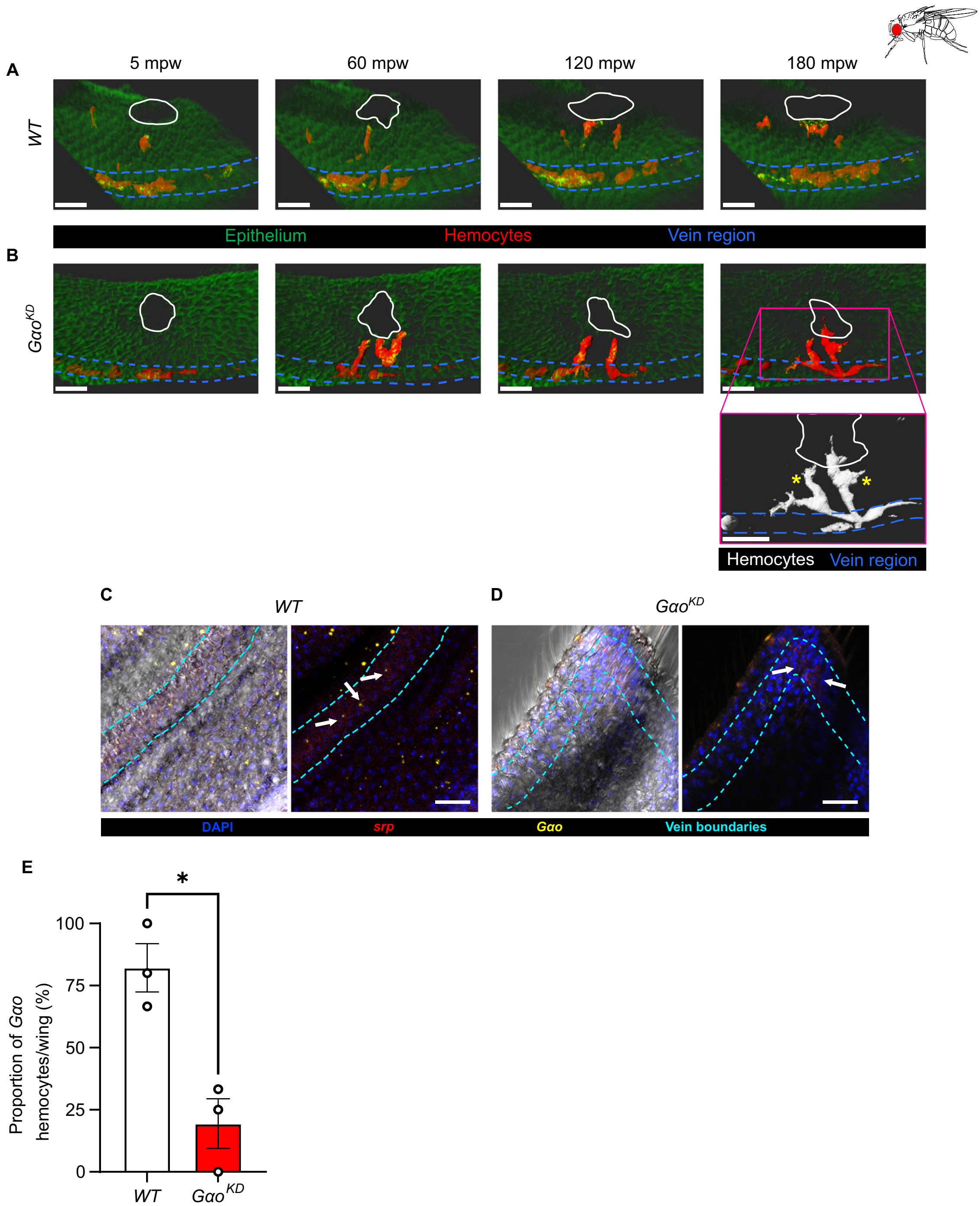

Figure EV4. Downstream effectors of Tre1.

(**A**, **B**) IMARIS renderings of 75 h APF pupae with *Gao* silenced hemocytes (*srp-Gal4*-driven RNAi silencing) as they respond to wounds, with labeling of epithelium (green, *ubi-GMA*), hemocytes (red, *srp-Gal4>mCherry, srp-mch*), veins (manually labeled with a blue dotted line), wound perimeter (solid white line), and hemocytes attempting to extravasate (yellow asterisks). (**A**) *WT* hemocytes are recruited to the wound and fully detached from the vasculature (**B**), whereas Gao^{KD} hemocytes show reduced recruitment and defective de-adhesion after leaving the vasculature. (**C**, **D**) Representative images of *WT* (**C**) or Gao^{KD} (**D**) 75 h APF wings labeled by HCR in situ hybridization for *Gao* (yellow) and the hemocyte marker *srp* (red), nuclei were counterstained with DAPI (blue), and brightfield was used to identify vasculature (manually labeled; white dotted line). In WT wings, *Gao* was found to be lowly expressed by both hemocytes and epithelium, but reduced expression of *Gao* was observed in the Gao^{KD}. (**E**) Quantification of the proportion of $Gao^{positive}$ hemocytes between *WT* and Gao^{KD} pupae ($P = 0.0108$), validating reduced expression when using the RNAi line. In total, 53 cells from 3 wings for each genotype were analyzed, and data show mean ± SEM, which was analyzed by an unpaired *t* test. $^*P < 0.05$. Scale bars: (**A**-**D**) = 15 µm.

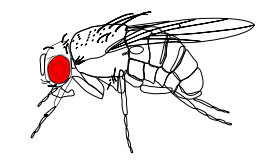

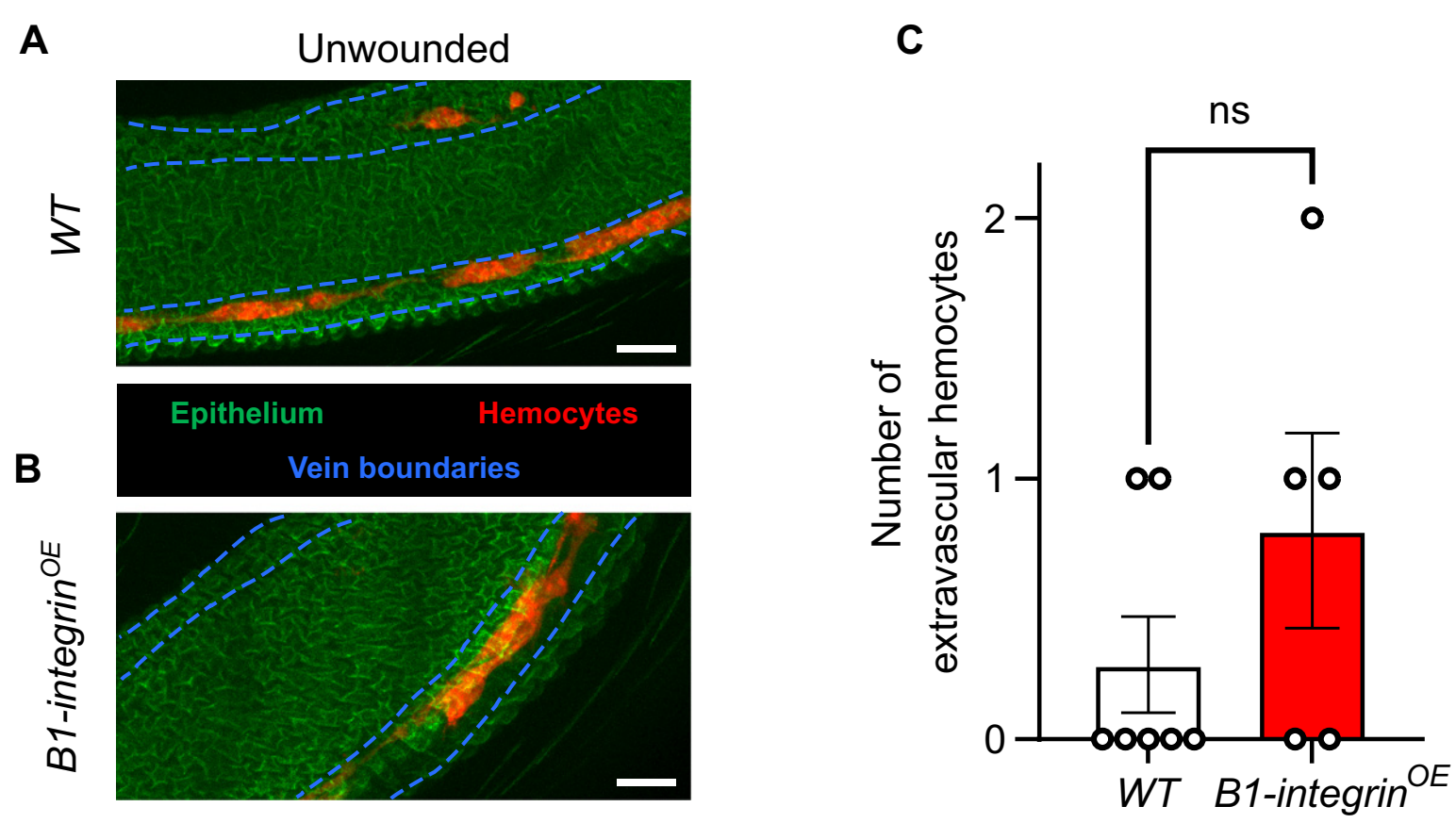

Figure EV5. Overexpression of ß1-integrin in hemocytes does not impact their tissue distribution.

(**A**, **B**) Representative images of 75 h APF wing vasculature with no significant difference in the number of extravascular hemocytes between *WT* (**A**) and ß1-integrin^{OE} (**B**) hemocytes. Extravascular refers to hemocytes, which are outside of the vasculature. (**C**) Quantification of the number of extravascular hemocytes in *WT* and *B1*-integrin^{OE} genetic backgrounds. Between 5 and 7 animals were analyzed for each genotype. Data represent mean ± SEM, which was analyzed by an unpaired Mann–Whitney test. Scale bars: (**A**, **B**) = 15 µm.

 © The Author(s)
