# Supplementary material for: Orphan GPR84 facilitates uropod de-adhesion at multiple steps during leukocyte extravasation
Source: EMBO Rep. 2026 Sep 12;27(20):6018–49. doi: 10.1038/s44319-026-00923-0 (PMC13694715; doi:10.1038/s44319-026-00923-0)
Supplement: Supplementary file 2 [file 44319_2026_923_MOESM1_ESM.pdf]

EMBO reports

Orphan GPR84 facilitates uropod de-adhesion at multiple steps during leukocyte extravasation

Clare Latta, Terrence Trinca, Francesca Robertson, Rachel Lau, Anna Barkaway, Loïc Rolas, Matthew Golding, Paul Imbert, Haitao Wang, Almke Bader, Liam Hill, Lorna Hodgson, Yue Yang, Aleksandar Ivetic, Barbara Walzog, Myles Lewis, Mathieu Voisin, Paul Martin, Sussan Nourshargh, and Helen Weavers

Corresponding author(s): Helen Weavers (helen.weavers@bristol.ac.uk) , Paul Martin (paul.martin@bristol.ac.uk), Sussan Nourshargh (s.nourshargh@qmul.ac.uk)

Review Timeline:

Submission Date:	26th Jan 26
Editorial Decision:	2nd Mar 26
Revision Received:	26th Jun 26
Editorial Decision:	4th Aug 26
Revision Received:	24th Aug 26
Accepted:	27th Aug 26

Editor: Achim Breiling

Transaction Report:

(Note: With the exception of the correction of typographical or spelling errors that could be a source of ambiguity, letters and reports are not edited. Depending on transfer agreements, referee reports obtained elsewhere may or may not be included in this compilation. Referee reports are anonymous unless the Referee chooses to sign their reports.)

Dear Dr. Weavers,

Thank you for the submission of your manuscript to EMBO reports. I have now received reports from the three referees that were asked to evaluate your study, which can be found at the end of this email. As you will see, the referees have several comments, concerns, and suggestions, indicating that a major revision of the manuscript is necessary to allow publication of the study in EMBO reports. As the reports are below, and all points need to be addressed, I will not detail them here.

Given the constructive referee comments, I would like to invite you to revise your manuscript with the understanding that the concerns of the referees must be addressed in the revised manuscript and in a detailed point-by-point response. Acceptance of your manuscript will depend on a positive outcome of a second round of review. It is EMBO reports policy to allow a single round of revision only and acceptance of the manuscript will therefore depend on the completeness of your responses included in the next, final version of the manuscript.

Revised manuscripts should be submitted within three months of a request for revision. Please contact me to discuss the revision (also by video chat) if you have questions or comments regarding the revision, or should you need additional time.

When submitting your revised manuscript, please also carefully review the instructions that follow below.

PLEASE NOTE THAT upon resubmission revised manuscripts are subjected to an initial quality control prior to exposition to re-review. Upon failure in the initial quality control, the manuscripts are sent back to the authors, which may lead to delays. Frequent reasons for such a failure are the lack of the data availability section (please see below) and the presence of statistics based on n=2 (the authors are then asked to present scatter plots or provide more data points).

When submitting your revised manuscript, we will require:

1) a .docx formatted version of the final manuscript text (including legends for main figures, EV figures and tables), but without the figures included. Figure legends should be compiled at the end of the manuscript text.

2) individual production quality figure files as .eps, .tif, .jpg (one file per figure), of main figures and EV figures. Please upload these as separate, individual files upon re-submission.

The Expanded View format, which will be displayed in the main HTML of the paper in a collapsible format, has replaced the Supplementary information. You can submit up to 6 images as Expanded View. Please follow the nomenclature Figure EV1, Figure EV2 etc. The figure legend for these should be included in the main manuscript document file in a section called Expanded View Figure Legends after the main Figure Legends section. Additional Supplementary material should be supplied as a single pdf file labeled Appendix. The Appendix should have page numbers and needs to include a table of content on the first page (with page numbers) and legends for all content. Please follow the nomenclature Appendix Figure Sx, Appendix Table Sx etc. throughout the text, and also label the figures and tables according to this nomenclature.

For movie files please use the nomenclature 'Movie EVx' in all places and add callouts for each file in the manuscript text using this name. Please provide the legends for each movie as separate text file uploaded and ZIPped together with its corresponding movie. Do not provide movie legends in the main text file.

For more details, please refer to our guide to authors:
https://link.springer.com/journal/44319/submission-guidelines#cms-Revised-submissions

Please consult our guide for figure preparation:
https://media.springernature.com/original/springer-cms/rest/v1/content/27825798/data/v1

3) a .docx formatted letter INCLUDING the reviewers' reports and your detailed point-by-point responses to their comments. As part of the EMBO Press transparent editorial process, the point-by-point response is part of the Review Process File (RPF), which will be published alongside your paper.

4) a complete author checklist, which you can download from our author guidelines (https://link.springer.com/journal/44319/submission-guidelines#cms-Revised-submissions). Please insert page numbers in the checklist to indicate where the requested information can be found in the manuscript. The completed author checklist will also be part of the RPF.

Please also follow our guidelines for the use of living organisms, and the respective reporting guidelines:
https://link.springer.com/partners/embo-press/editorial-policies#Animal%20and%20human%20studies

5) that primary datasets produced in this study (e.g. RNA-seq, ChIP-seq, structural and array data) are deposited in an

appropriate public database. If no primary datasets have been deposited, please also state this in a dedicated section (e.g. 'No primary datasets have been generated and deposited'), see below.

See also: https://link.springer.com/partners/embo-press/editorial-policies#Data%20deposition

Please remember to provide a reviewer password if the datasets are not yet public.

The accession numbers and database should be listed in a formal "Data Availability" section that follows the model below. This is now mandatory (like the COI statement). Please note that the Data Availability Section is restricted to new primary data that are part of this study. This section is mandatory. As indicated above, if no primary datasets have been deposited, please state this in this section

Data availability

The datasets produced in this study are available in the following databases:

- RNA-Seq data: Gene Expression Omnibus GSE46843 (https://www.ncbi.nlm.nih.gov/geo/query/acc.cgi?acc=GSE46843)
- [data type]: [name of the resource] [accession number/identifier/doi] ([URL or identifiers.org/DATABASE:ACCESSION])

*** Note - All links should resolve to a page where the data can be accessed. ***

See also: https://link.springer.com/partners/embo-press/editorial-policies#Data%20availability%20statement

Moreover, I have these editorial requests:

6) We now request the publication of original source data with the aim of making primary data more accessible and transparent to the reader. You will receive a separate email with instructions for providing source data with your revised manuscript, including information how to upload and organize the files.

7) Our journal encourages inclusion of *data citations in the reference list* to directly cite datasets that were re-used and obtained from public databases. Data citations in the article text are distinct from normal bibliographical citations and should directly link to the database records from which the data can be accessed. In the main text, data citations are formatted as follows: "Data ref: Smith et al, 2001" or "Data ref: NCBI Sequence Read Archive PRJNA342805, 2017". In the Reference list, data citations must be labeled with "[DATASET]". A data reference must provide the database name, accession number/identifiers and a resolvable link to the landing page from which the data can be accessed at the end of the reference. Further instructions are available at: https://link.springer.com/journal/44319/submission-guidelines#cms-Reference-guidelines

8) Regarding data quantification and statistics, please make sure that the number "n" for how many independent experiments were performed, their nature (biological versus technical replicates), the bars and error bars (e.g. SEM, SD) and the test used to calculate p-values is indicated in the respective figure legends (also for EV and Appendix figures). Please also check that all the p-values are explained in the legend, and that these fit to those shown in the figure. Please provide statistical testing where applicable. Please avoid the phrase 'independent experiment' but clearly state if these were biological or technical replicates. Please also indicate (e.g. with n.s.) if testing was performed, but the differences are not significant. In case n=2, please show the data as separate datapoints without error bars and statistics. See also: https://link.springer.com/journal/44319/submission-guidelines#cms-Figure-and-data-presentation

If n<5, please show single datapoints for diagrams.

9) Please add scale bars of similar style and thickness to microscopic images, using clearly visible black or white bars (depending on the background). Please place these in the lower right corner of the images themselves. Please do not write on or near the bars in the image but define the size in the respective figure legend.

10) Please also note our reference format: https://link.springer.com/journal/44319/submission-guidelines#cms-Reference-guidelines

11) We updated our journal's competing interests policy in January 2022 and request authors to consider both actual and perceived competing interests. Please name this section 'Disclosure and Competing Interests Statement' and put it after the Acknowledgements section.
See: https://link.springer.com/partners/embo-press/editorial-policies#Competing%20interest%20disclosures

12) We now use CRediT to specify the contributions of each author in the journal submission system. CRediT replaces the author contribution section. Please use the free text box to provide more detailed descriptions and do NOT provide your final manuscript text file with an author contributions section. See also our guide to authors (section 'Author contributions'): https://link.springer.com/journal/44319/submission-guidelines#cms-Revised-submissions

13) All Materials and Methods need to be described in the main text using our 'Structured Methods' format, which is required for all research articles. According to this format, the Methods section should include a Reagents and Tools Table (listing key reagents, experimental models, software, and relevant equipment and including their sources and relevant identifiers), uploaded as separate file, and a Methods section in which we encourage the authors to describe their methods using a step-by-step protocol format with bullet points, to facilitate the adoption of the methodologies across labs. More information on how to adhere to this format as well as downloadable templates (.doc) for the Reagents and Tools Table can be found in our author guidelines (section 'Structured Methods'):

https://link.springer.com/journal/44319/submission-guidelines#cms-Manuscript-organisation-and-formatting

Please add all primer or antibody information directly to the Reagents and Tools table.

14) Please order the manuscript sections like this, using only these names:
Title page - Abstract (max. 175 words) - Keywords (max. 5) - Introduction - Results - Discussion - Methods - Data availability section - Acknowledgements (please include here all the funding information) - Disclosure and Competing Interests Statement - References - Figure legends - Expanded View Figure legends

15) Please make sure that all the funding information is also entered into the online submission system and that it is complete and similar to the one in the acknowledgement section of the manuscript text file.

We would also welcome the submission of cover suggestions, or motifs to be used by our Graphics Illustrator in designing a cover.

As part of the EMBO publication's Transparent Editorial Process, EMBO reports publishes online a Review Process File (RPF) to accompany accepted manuscripts. This File will be published in conjunction with your paper and will include the referee reports, your point-by-point response and all pertinent correspondence relating to the manuscript.

You are able to opt out of this by letting the editorial office know (emboreports@embo.org). If you do opt out, the Review Process File link will point to the following statement: "No Review Process File is available with this article, as the authors have chosen not to make the review process public in this case."

I look forward to seeing a revised form of your manuscript when it is ready.

Yours sincerely,

Achim Breiling
Senior Editor
EMBO Reports

Referee #1:

Latta et al. present intriguing work suggesting a unifying role for the GPCR, GPR84, and its fly orthologue, Tre1, in regulating uropod detachment during leukocyte diapedesis. Previous work from this group established a new fly model system to study hemocyte extravasation and identified Tre1 as an important regulator of tail detachment. The current study extends this work to the mammalian orthologue, GPR84, and further delves into molecular mechanisms, identifying commonalities in the function of these GPCRs in leukocyte extravasation between flies and mice. The live imaging data and phenotypes are very compelling. However, some of the claims are not yet fully supported by the data. I outline my comments below to bolster this manuscript.

Comments:
1. Fig. 1F: The differences in hemocyte cell circularity between the Tre1 mutant and the hemocyte-specific Tre1 KD pre-wounding suggest an incomplete knockdown (also shown by quantification in the previous work (Thuma et al. 2018)), a role for Tre1 in vein cells, or a potential earlier role in hemocytes before the KD is complete, with a persistent effect. One would imagine a GPCR involved in extravasation would be activated in response to wounding, so the pre-wounding phenotype is curious. Otherwise, Tre1 may already be active when hemocytes are circulating, thereby constitutively affecting adhesion levels.

a. I suggest the authors use UAS/Gal4 to rescue Tre1 expression in hemocytes or vein cells in Tre1 mutant animals to tease apart the autonomy of this phenotype.
b. Is there a difference in Tre1 mutant hemocyte migration speed pre-wounding vs. control?

2. Is there a difference in the number of hemocytes that try to extravasate in Tre1 KD or Tre1 null hemocytes vs control? This would help to decouple whether Tre1 is involved in sensing the wound.

3. Line 27-28: Is there a specific effect on extravasation if there is a phenotype before wounding? This suggests an effect on hemocyte adhesion levels overall that may affect all migratory responses, with more pronounced effects in events that require stronger detachment, such as extravasation.

4. Figure 2B: It is difficult to appreciate the context of Figure 2B without a comparison. How does the structure of Tre1 compare to an unrelated Class A GPCR?

5. Figure 3: Is there an animal limitation to comparing Gpr84 mutant + TNF vs. Gpr84 mutant + PBS in the cremaster model? I imagine this would be a more appropriate comparison and also reveal baseline data for the Gpr84 mutant mice.

6. Are there differences in intraluminal morphology in GPR84 mutant neutrophils pre-TNF? This would be an apt comparison to the morphology of Tre1 mutant hemocytes pre-wounding.

7. The descriptive observations in Fig. 4B and in the text would benefit from quantification. How often are these fragments and long tethers observed?

8. In Fig. 4G, the fact that WT neutrophils have more TEM events than GPR84 null neutrophils, yet a similar number of TPM events, suggests a significant portion of WT neutrophils are stuck between the EC layer and the pericyte layer. Conversely, are the GPR84 mutant neutrophils more effective at initiating TPM?

9. Line 110: How do the authors define amoeboid morphology?

10. In the TEM images, how are the authors able to discern that the fragments came from neutrophils in Fig. 5F? One can also observe several small circular fragments in Figure 5Di in WT neutrophils.

11. Figure 7B, the authors should include confidence metrics for this interaction prediction. How often are these results obtained between simulations? What about other G-alpha subunits? Does G-alpha-o give the best fit vs. the others?

12. I have issues with the model proposed in 7B based on the known biology of RhoGEF2 in Drosophila. RhoGEF2 associates with microtubule + ends by binding to Eb1 and is canonically activated by concertina (Drosophila Galpha12/13) [1]. Previous work has suggested a connection between Tre1 and RhoGEF2, but signaling pathways directly linking them are unclear [2]. Mechanistically, how would RhoGEF2 associate with Tre1 and be activated by G-alpha-o? The RhoGEF2 connection could be strengthened by a RhoGEF2 KD in hemocytes and an epistasis experiment with a Tre1 mutant background to see if the phenotypes are exacerbated.

[1] Rogers SL, Wiedemann U, Häcker U, Turck C, Vale RD. Drosophila RhoGEF2 Associates with Microtubule Plus Ends in an EB1-Dependent Manner. Current Biology. 2004;14(20):1827-33.
[2] Lin B, Luo J, Lehmann R. An AMPK phosphoregulated RhoGEF feedback loop tunes cortical flow-driven amoeboid migration in vivo. Science Advances. 2022;8(37):eabo0323.

13. The degree of knockdown of Galpha-o in RNAi experiments in Figure 7 should be quantified. If qPCR on isolated hemocytes is not possible, RNA FISH could be used.

14. Figure 7L: What is a "0" second flash? How is a flash defined, by pixel intensity vs. background? Figure 7M: What is a "0" area flash? My guess is these are cells that never displayed a puncta, but again the criteria for these puncta have not been outlined.

15. Given that the putative ligand for GPR84 is known (medium chain fatty acids), the authors can provide more evidence for a connection between GPR84 and RhoA by adding the GPR84 ligand to isolated neutrophils and assessing GTP-bound RhoA. If this response is absent in GPR84 mutant neutrophils, this is stronger evidence for this connection. Alternatively, if GPR84 is constitutively active (as suggested by transcriptomics), assessing GTP-bound RhoA levels in GPR84 mutant vs control neutrophils may illuminate whether there is a difference.

16. Fig. 8C: Are the number of hemocytes recruited to the wound the same in mys OE vs. control? By eye, there seem to be more in the control. The authors could normalize to the initial number at the wound to account for this.

17. Fig. 8E-F, the reductions are very modest. The effects may be more apparent if the authors use a hemocyte-specific quantification, instead of the whole animal. These adhesion reductions would also point to a Tre1 -dependent effect that is not wounding specific.

18. Fig. 8G: Can the authors indicate which cells count as "spread" or "motile"? Looking at the image, the gpr84 mutant neutrophils that are rounded (which I assume is not spread), look to have the same surface area as the WT cells.

19. Discussion line 14-15: Localized Rho1/RhoA activity only applies to Tre1 as the connection to RhoA was not demonstrated in GPR84.

Minor comments:
1. Please add the actual number of cells analyzed in the legends.
2. Figure 7 legend states that there are no active Rho1 flashes in Tre1-KO, but there are non-zero times and areas in the plots.
3. Fig. 9E: The red box used to show where the inset is from is very difficult to see in the whole image. I suggest switching to blue hues, such as cyan.
4. The references on lines 160 and 163 are duplicated.

Referee #2:

Latta and colleagues report a series of elegant experiments that identify GPR84 *and the orthologue Tre1) as regulator of the final stages of neutrophil extravasation through inflamed blood vessels in mice and Drosophila. They show that GPR84 supports Rho1-mediated detachment of leukocyte uropod from vessel exit sites. Genetic deletion or pharmacological blockade of GPR84 in mice or Tre1-KO in Drosophila markedly impaired detachment of transmigrating leukocytes through defective cytoskeletal regulation and defective detachment from the endothelium. These findings are of interest and raise the intriguing possibility of therapeutic targeting of GPR84 for modulating leukocyte trafficking into inflamed tissues. There are, however, few issues that may require further attention.

1. GPR84-deletion led to both increases (exaggerated uropod) or decreases (amoeboid with perivascular arrest) in the length of perivascular neutrophils. Would these represent two distinct stages of the same process? What would be the molecular switch between these stages?
2. What was the fate of neutrophils that cannot detach in the inflamed vessel? It appears that few neutrophils might have undergone reverse trans-endothelial migration in Gpr84-/- mice in Supplementary Movie S5.
3. Since there are some differences in the mechanisms underlying neutrophil trafficking in postcapillary venules and arteries, some comments would be warranted on whether GPR84 may play similar role in the arterial wall as in capillaries.
4. The Discussion appears to be shifting focus in GPR84 regulation of myeloid cells ranging from myelopoiesis through neutrophil extravasation to macrophage recruitment in wound healing. These should be better aligned with the data presented.

Referee #3:

This is a very interesting manuscript from Latta and Trinca identifying a role for an orphan GPCR (CPR84) in activating Rho A to allow uropod de-attachment during several stages of leukocyte extravasation. The investigation uses both Drosophila and mouse models and comes up with a consistent story. Tre1-deficient Drosophila pupae were shown to have defective wound healing due to ineffective hemocyte migration out of the "vasculature" with a phenotype showing elongated hemocyte bodies with long uropods apparently still tethered to the inside of the vessel and/or extracellular matrix. This phenotype was reproduced in mice genetically deficient in GPR84, the mouse homologue of Tre1. Prolonged interactions of mouse neutrophils were seen at all stages of leukocyte contact with endothelium, but quantified during transendothelial migration, transpericyte migration, and detatchment from the venular wall. The authors then went back to the fly and knocked down Gαo, predicted to be the G protein coupled to Tre1 and observed the same phenotype. They also show that Rho1 (the Drosophila homologue of Rho A) signaling is defective in the Tre1 knockout flies and β1-integrin overexpressing fly pupae, building a model that Tre1/GPR84 signals through Gαo to activate Rho for integrin activation and cytoskeletal changes.

The manuscript is generally easy to read, flows nicely, and the data justify the conclusions. The videos are clear and really demonstrate the phenotype. I have several points for the authors to address, but mostly minor issues. Nothing that requires new experimentation.

The most important comment is that beyond the GPCR findings, much of this work was anticipated by a paper from Keith Burridge's group in 2001 (Worthylake et al.), which they cite once in the Results section, but I believe deserves at least a paragraph in the Discussion. Worthylake, et al. showed that inhibiting RhoA in monocytes blocked transendothelial migration as well as migration on a solid substratum. The phenotype was exactly what the authors show here-uropods with β2 integrins stuck in an elongated trailing end. They also showed that Rho kinase was required for this process. The manuscript from Latta and Trinca clearly advances the field, identifying GPR84 as an upstream regulator of Rho, and they demonstrate the phenomenon at many points of the extravasation process in vivo. However, a full discussion of the previous literature in the context of the new findings takes nothing away from the impact of the authors' new findings. It would be valuable for those new to the field or those who do not pay attention to papers that are 25 years old.

The next most important comment is regarding the rolling and intraluminal crawling. The authors say that the Gpr84 KO mice had abnormal intraluminal neutrophil structures, and some images are shown in Figure 4A, B. Do they have any statistics on how, if at all, the rolling flux, rolling rate, and crawling were affected in these mice? CD18 integrins are implicated in slow rolling

and one might expect aberrant deactivation could lead to slower rolling and abnormal intraluminal crawling rates. The authors would not have to perform additional experiments to obtain the data, merely reanalyze existing videos. If this is at all feasible, I recommend that they add such data to the story to make it complete.

Since they demonstrate that GPR84 has a role in multiple steps in the extravasation process, not just diapedesis, they should change the title to reflect that. I recommend, "Orphan GPR84 facilitates uropod de-adhesion at multiple steps in during (inflammatory) leukocyte extravasation."

Results lines 91 - 94 corresponding to Figs 4G, H: This sentence is confusing since it is hard to tell whether they use "breaching" to mean making a defect in the pericyte layer or to mean going through. Since they define this as trans-pericyte migration, it seems that they mean PMN migrating across the pericyte layer. However, then the "incomplete" TPM does not make sense. Please change the terms to make it clear what you mean.

Figure 1 legend, panel E. To truly show that the morphologies are distinct, it would be good to show WT leukocytes rendered in the same way.

Figure 1 legend, panel N. Are we supposed to see a wound. I do not see anything that appears green, but the box over the figure shows green as wound. Also, please point out the "vein" lumen in panels L, M, N.

Faculty of Biomedical Sciences
University of Bristol
University Walk
Bristol
BS8 1TD UK

26th June 2026

RE: Manuscript EMBOR-2026-63676V1
Point-by-Point Response to The Referee's Comments

The revised manuscript incorporates 22 new data sets/image panels that directly address the referee's points. Collectively, we have strengthened our description of Tre1's autonomous role within hemocytes by inserting new live imaging data of a hemocyte-specific Tre1 rescue. Furthermore, we have validated the efficiency of Gαo knockdown (via HCR) and provided new evidence that RhoGEF2 is additionally required for hemocyte extravasation. In mice, we have provided further evidence to support our overall conclusion that GPR84 is a regulator of neutrophil de-adhesion. Specifically, we present new data showing decreased neutrophil luminal crawling (i.e. excessive adhesion) and enhanced neutrophil-derived fragments in inflamed venules of GPR84 KO mice. Crucially, we provide direct *in vitro* evidence to show that GPR84 agonism of mouse neutrophils leads to RhoA activation. Together, these findings reinforce our conclusion that GPR84 is a previously unidentified regulator of neutrophil extravasation via RhoA activation.

Finally, we have made extensive changes to the manuscript text to enhance clarity, be more rigorous with some of our interpretations and better align the Discussion with the Results. We are grateful for the reviewers' suggestions that led us to perform the additional experiments and analysis that we believe have greatly improved the strength and impact of the paper. Of note, in preparing the Source Data file, we identified an error in the graphical presentation of our neutrophil TEM data (Figure 4F). With this error rectified, the comparison of WT vs KO neutrophils is now significant.

The text below provides a detailed point-by-point response (**bold**) to the reviewer's comments. We have also highlighted all changes in the manuscript in yellow.

Referee #1:

Major Comments:

1. Fig. 1F: The differences in hemocyte cell circularity between the Tre1 mutant and the hemocyte-specific Tre1 KD pre-wounding suggest an incomplete knockdown (also shown by quantification in the previous work (Thuma et al. 2018), a role for Tre1 in vein cells, or a potential earlier role in hemocytes before the KD is complete, with a persistent effect. One would imagine a GPCR involved in extravasation would be activated in response to wounding, so the pre-wounding phenotype is curious. Otherwise, Tre1 may already be active when hemocytes are circulating, thereby constitutively affecting adhesion levels.

a. I suggest the authors use UAS/Gal4 to rescue Tre1 expression in hemocytes or vein cells in Tre1 mutant animals to tease apart the autonomy of this phenotype.

We agree that the exaggeration of the pre-wound circulatory phenotype in the Tre1 KO compared to the hemocyte-specific Tre1 RNAi most likely reflects the incomplete Tre1 knockdown in the RNAi (as we showed previously by qPCR in Thuma et al 2018). Nevertheless, as correctly suggested here, it could also indicate a non-autonomous role for Tre1 (e.g. in vein cells). To

address, we have ectopically expressed Tre1 specifically within hemocytes (using the Gal4 UAS system) in a Tre1 KO background. This rescue experiment not only restored the pre-wound circularity of luminal hemocytes (Fig 1F), but it also rescued both the number of hemocytes fully recruited to wounds (Fig 1G) and the time taken to extravasate towards wounds (Fig 1H) (discussed in the text on page 5).

b. Is there a difference in Tre1 mutant hemocyte migration speed pre-wounding vs. control?

Yes, our analyses and new quantifications show that prior to wounding, Tre1 mutant hemocytes exhibit greater adhesion to the vessel wall as compared to controls (Fig EV1E and described in the text page 5).

2. Is there a difference in the number of hemocytes that try to extravasate in Tre1 KD or Tre1 null hemocytes vs control? This would help to decouple whether Tre1 is involved in sensing the wound.

We agree that it is important to establish whether Tre1 influences initial wound "sensing" or "recruitment" as this would have a knock-on effect on extravasation. To address, we have re-analysed our imaging data and have found that the number of hemocytes attempting to extravasate towards wounds over time are not significantly different between Tre1 knockdown (or null) hemocytes compared to WT hemocytes (as shown in Figure EV1F and described in the text on page 5).

3. Line 27-28: Is there a specific effect on extravasation if there is a phenotype before wounding? This suggests an effect on hemocyte adhesion levels overall that may affect all migratory responses, with more pronounced effects in events that require stronger detachment, such as extravasation.

We thank the Referee for this valid point and agree that adhesion is likely the principal response impacted. This would influence all stages of the extravasation process as well as prior migratory responses, as now indicated in the revised title and the text (e.g. see first paragraph of page 15 in the Discussion).

4. Figure 2B: It is difficult to appreciate the context of Figure 2B without a comparison. How does the structure of Tre1 compare to an unrelated Class A GPCR?

We have now included another unrelated Class A GPCR, *Drosophila* Rhodopsin (and its mammalian orthologues) in our comparative analyses and we find a clear segregation. Our revised Figure 2 includes these routing sequences in the dendrogram (Fig 2A), which has helped to further highlight the close relationship between Tre1 and its mammalian orthologues. We have also included an additional AlphaFold model comparison for Tre1 against *Drosophila* Rhodopsin (Fig 2B). This additional comparison demonstrates less structural overlap than our original Tre1 vs. human GPR84 comparison, again further highlighting the orthology of these proteins.

5. Figure 3: Is there an animal limitation to comparing Gpr84 mutant + TNF vs. Gpr84 mutant + PBS in the cremaster model? I imagine this would be a more appropriate comparison and also reveal baseline data for the Gpr84 mutant mice.

Figure 3 now incorporates control PBS data from Gpr84$^{-/-}$ mice, showing no significant difference in the number of adherent or extravasated leukocytes as compared to WT PBS groups (Figures 3C and 3D).

6. Are there differences in intraluminal morphology in GPR84 mutant neutrophils pre-TNF? This would be an apt comparison to the morphology of Tre1 mutant hemocytes pre-wounding.

Analysis of brightfield intravital microscopy recordings of control (PBS-treated) murine cremaster muscles revealed no notable differences in leukocyte morphologies between genotypes, suggesting a variance in the functional role of GPR84 in regulating baseline intraluminal immune cell-vessel wall interactions in the fly vs mice. This may be accounted for by the differences in molecular and cellular composition of fly vs mouse small veins.

7. The descriptive observations in Fig. 4B and in the text would benefit from quantification. How often are these fragments and long tethers observed?

The revised manuscript provides quantification of these intraluminal observations. With respect to fragments, we see a strong trend towards an increased number in inflamed KO venules (p=0.085; new Figure 4C). With respect to neutrophil tethers, whilst we saw no significant difference in their number in inflamed WT vs *Gpr84*$^{-/-}$ post-capillary venules, we observed a trend towards increased length of the tethers in KO neutrophils. Specifically, 35% and 43% of WT and *Gpr84*$^{-/-}$ neutrophil tethers, respectively, were ≥ ~10 µm in length. This quantification is inserted in the text in the relevant Results section.

8. In Fig. 4G, the fact that WT neutrophils have more TEM events than GPR84 null neutrophils, yet a similar number of TPM events, suggests a significant portion of WT neutrophils are stuck between the EC layer and the pericyte layer. Conversely, are the GPR84 mutant neutrophils more effective at initiating TPM?

This is a valid point. We have now quantified the number of neutrophils in the sub-endothelial space at the experimental endpoint (3.5-5 hrs post stimulation) and the data (not presented) shows no significant difference between WT and KO mice. We believe that this is because KO cells exhibit an inefficient TPM, showing multiple oscillatory events at the pericyte layer (as shown in Fig 4K).

9. Line 110: How do the authors define amoeboid morphology?

We have used "amoeboid" to describe neutrophils characterised by a rounded morphology lacking distinct polarising features such as a leading edge and uropod. We accept that this may be ambiguous and so have now changed the term in the text and Figure 5B to "spherical" morphology.

10. In the TEM images, how are the authors able to discern that the fragments came from neutrophils in Fig. 5F? One can also observe several small circular fragments in Figure 5Di in WT neutrophils.

We agree that it is not possible for us to categorically confirm that the fragments are derived from neutrophils. As such, we have limited the false colouring of neutrophils in the EM images to the extended neutrophil tails (in the KO tissues) only.

11. Figure 7B, the authors should include confidence metrics for this interaction prediction. How often are these results obtained between simulations? What about other G-alpha subunits? Does G-alpha-o give the best fit vs. the others?

We apologise for this accidental omission and have now included the confidence metrics in the Figure legend. We did not originally test the other G-alpha subunits as there was already suggestion from the literature that Gαo directly binds Tre1, and that Gαo is downstream of the closely-related paralogue moody [Refs 1 and 2 below]. We have now tested multiple protein complex models, where Gαo has been substituted with the five other *Drosophila* Gα proteins. We

find that replicate models tend to converge on Gαo, Gαs and Concertina as the most stable models. Nevertheless, given the observed similarity in defective extravasation between Tre1 mutant and Gαo knockdown hemocytes, together with the published evidence [Refs 1 and 2 below] and *in silico* modelling, these data synergistically support Gαo as a key G-protein working downstream of Tre1. We have now modified the text accordingly [page 10].

[1] Yoshiura S, Ohta N, Matsuzaki F. Tre1 GPCR Signaling Orients Stem Cell Divisions in the Drosophila Central Nervous System. Developmental Cell, 2011; 22, 79-91
[2] Schwabe T, Bainton RJ, Fetter RD, Heberlein U, Gaul U. GPCR signaling is required for blood-brain barrier formation in Drosophila. Cell. 2005 Oct 7;123(1):133-44.

12. I have issues with the model proposed in 7B based on the known biology of RhoGEF2 in Drosophila. RhoGEF2 associates with microtubule + ends by binding to Eb1 and is canonically activated by concertina (Drosophila Galpha12/13) [1]. Previous work has suggested a connection between Tre1 and RhoGEF2, but signaling pathways directly linking them are unclear [2]. Mechanistically, how would RhoGEF2 associate with Tre1 and be activated by G-alpha-o? The RhoGEF2 connection could be strengthened by a RhoGEF2 KD in hemocytes and an epistasis experiment with a Tre1 mutant background to see if the phenotypes are exacerbated.

[1] Rogers SL, Wiedemann U, Häcker U, Turck C, Vale RD. Drosophila RhoGEF2 Associates with Microtubule Plus Ends in an EB1-Dependent Manner. Current Biology. 2004;14(20):1827-33.
[2] Lin B, Luo J, Lehmann R. An AMPK phosphoregulated RhoGEF feedback loop tunes cortical flow-driven amoeboid migration in vivo. Science Advances. 2022;8(37):eabo0323.

Since our live imaging of the Rho activity reporter suggested that Rho was activated at the uropod detachment site, we hypothesised that a Rho GEF might be activating it. As you suggest, we have attempted to confirm this using a RhoGEF2 knockdown experiment and this shows a significant reduction in fully recruited hemocytes at wounds, similar to that following knock-down of Gαo or Tre1. We have now included this in our Figures (7D-G) and updated the text accordingly (page 10).

13. The degree of knockdown of Galpha-o in RNAi experiments in Figure 7 should be quantified. If qPCR on isolated hemocytes is not possible, RNA FISH could be used.

It is particularly challenging to collect living hemocytes in sufficient numbers to perform RT-qPCR, which is why we have resorted to whole animal studies for other RT-qPCR experiments. Nevertheless, we have now undertaken alternative expression analyses of Gao (using HCR in situ with probes against Gαo mRNA) in both control and hemocytes expressing Gao-RNAi and found a significant reduction in Gαo transcripts (punctae) following RNAi (Fig EV4C-E).

14. Figure 7L: What is a "0" second flash? How is a flash defined, by pixel intensity vs. background? Figure 7M: What is a "0" area flash? My guess is these are cells that never displayed a puncta, but again the criteria for these puncta have not been outlined.

Apologies for our brevity. We now include the definition of these punctae more comprehensively in the legend of Figure 7. We define a puncta as "any event which occurs over two consecutive time frames with an average intensity above 40 a.u.".

15. Given that the putative ligand for GPR84 is known (medium chain fatty acids), the authors can provide more evidence for a connection between GPR84 and RhoA by adding the GPR84 ligand to isolated neutrophils and assessing GTP-bound RhoA. If this response is absent in GPR84 mutant neutrophils, this is stronger evidence for this connection. Alternatively, if GPR84 is constitutively active (as suggested

by transcriptomics), assessing GTP-bound RhoA levels in GPR84 mutant vs control neutrophils may illuminate whether there is a difference.

We thank the Reviewer for this suggestion. In addressing, using a RhoA G-LISA (G-protein Linked Immunosorbent Assay), GPR84 KO bone-marrow derived mouse neutrophils exhibited reduced GTP-bound RhoA following stimulation with the potent GPR84 agonist L237, as compared to WT cells (new Figure 7O). Notably, within this assay, both WT and KO neutrophils responded equally to the potent synthetic FPR activator, WKYMVm, showing that GPR84-deficient mouse neutrophils have no intrinsic abnormalities in RhoA activation.

16. Fig. 8C: Are the number of hemocytes recruited to the wound the same in mys OE vs. control? By eye, there seem to be more in the control. The authors could normalize to the initial number at the wound to account for this.

Apologies for the confusion here. Due to technical constraints our first timepoint is 5-10 mins post-wounding by which time some hemocytes have already been recruited to the (control) wound. In unwounded pupae, we have quantified the number of extravascular hemocytes (i.e. those hemocytes already outside of the vessel) in WT versus mysOE backgrounds and find no significant difference (Figure EV5). We have added additional images and quantifications (Figure EV5) and amended the text accordingly [page 11].

17. Fig. 8E-F, the reductions are very modest. The effects may be more apparent if the authors use a hemocyte-specific quantification, instead of the whole animal. These adhesion reductions would also point to a Tre1 -dependent effect that is not wounding specific.

We agree that a more dramatic difference in adhesion is likely observed using a hemocyte-specific quantification. However, as described above, collecting sufficient high-quality cells for such RT-qPCR has proven particularly challenging in our hands. As described above, we have also modified the text (e.g. Discussion page 15) to discuss how Tre1/GPR84 might impact leukocyte adhesion during all migratory responses, although more pronounced effects might be apparent during events that require stronger detachment, such as extravasation.

18. Fig. 8G: Can the authors indicate which cells count as "spread" or "motile"? Looking at the image, the gpr84 mutant neutrophils that are rounded (which I assume is not spread), look to have the same surface area as the WT cells.

We have now provided a more representative image and indicated “spread” or “motile” with arrows. The surface area of each individual neutrophil has been quantified at the experimental endpoint (15 min post-CXCL1 stimulation) indicating a trend towards a reduction in GPR84-deficient neutrophils, although this was not significant. The numbers are presented in the revised text. Additionally, for clarity, we have provided description of “spread” and “motile” cells in the Methods.

19. Discussion line 14-15: Localized Rho1/RhoA activity only applies to Tre1 as the connection to RhoA was not demonstrated in GPR84.

As mentioned above, the revised manuscript now includes data showing RhoA activation post GPR84 agonism using a RhoA G-LISA (new Figure 7O).

<u>Minor comments:</u>

1. Please add the actual number of cells analyzed in the legends.

This has now been done.

2. Figure 7 legend states that there are no active Rho1 flashes in Tre1-KO, but there are non-zero times and areas in the plots.
We have now altered the legend to accommodate this.

3. Fig. 9E: The red box used to show where the inset is from is very difficult to see in the whole image. I suggest switching to blue hues, such as cyan.
This has been changed.

4. The references on lines 160 and 163 are duplicated.
This error has been rectified.

Referee #2:

1. GPR84-deletion led to both increases (exaggerated uropod) or decreases (amoeboid with perivascular arrest) in the length of perivascular neutrophils. Would these represent two distinct stages of the same process? What would be the molecular switch between these stages?

This is an interesting question that is difficult to categorically address. However, our observations suggest that the "spherical" perivascular KO cells are neutrophils that are strongly adherent to venular walls and hence show no sign of polarisation. In contrast, the KO cells with "exaggerated uropods" are less adherent neutrophils that are attempting to disengage from the venular wall. Our real-time imaging studies did not provide categoric evidence of one switching to the other and hence one possibility is that the differences in the noted morphologies may be accounted for by intrinsic neutrophil heterogeneity and maturity. The latter is a notion we intend to explore in future studies.

2. What was the fate of neutrophils that cannot detach in the inflamed vessel? It appears that few neutrophils might have undergone reverse trans-endothelial migration in Gpr84-/- mice in Supplementary Movie S5.

The true fate of neutrophils retained at the perivascular wall (for >30 mins; Figure 5C) is unknown due to the acute nature (~2 hrs) of the imaging periods. However, our observations within this time frame indicate that they simply remain there for prolonged periods before eventually detaching from the post-capillary venular wall and migrating into the interstitial tissue. Our group has much interest and expertise in tracking neutrophil reverse TEM and to date we have not observed any notable frequency of this response within TNF-stimulated cremaster muscles of WT or KO mice in the present study.

3. Since there are some differences in the mechanisms underlying neutrophil trafficking in postcapillary venules and arteries, some comments would be warranted on whether GPR84 may play similar role in the arterial wall as in capillaries.

Neutrophil trafficking in the microcirculation occurs exclusively within postcapillary venules. As such, there is no evidence for GPR84 playing a role in neutrophil passage across the arterial wall and any comment on this would be highly speculative.

4. The Discussion appears to be shifting focus in GPR84 regulation of myeloid cells ranging from myelopoiesis through neutrophil extravasation to macrophage recruitment in wound healing. These should be better aligned with the data presented.

We have endeavoured to edit the Discussion for further alignment with the data.

Referee #3:

1. The most important comment is that beyond the GPCR findings, much of this work was anticipated by a paper from Keith Burridge's group in 2001 (Worthylake et al.), which they cite once in the Results section, but I believe deserves at least a paragraph in the Discussion. Worthylake, et al. showed that inhibiting RhoA in monocytes blocked transendothelial migration as well as migration on a solid substratum. The phenotype was exactly what the authors show here-uropods with β2 integrins stuck in an elongated trailing end. They also showed that Rho kinase was required for this process. The manuscript from Latta and Trinca clearly advances the field, identifying GPR84 as an upstream regulator of Rho, and they demonstrate the phenomenon at many points of the extravasation process in vivo. However, a full discussion of the previous literature in the context of the new findings takes nothing away from the impact of the authors' new findings. It would be valuable for those new to the field or those who do not pay attention to papers that are 25 years old.

We thank the Reviewer for this comment. The revised Discussion has an extended coverage of the seminal works of Keith Burridge and colleagues.

2. The next most important comment is regarding the rolling and intraluminal crawling. The authors say that the Gpr84 KO mice had abnormal intraluminal neutrophil structures, and some images are shown in Figure 4A, B. Do they have any statistics on how, if at all, the rolling flux, rolling rate, and crawling were affected in these mice? CD18 integrins are implicated in slow rolling and one might expect aberrant deactivation could lead to slower rolling and abnormal intraluminal crawling rates. The authors would not have to perform additional experiments to obtain the data, merely reanalyze existing videos. If this is at all feasible, I recommend that they add such data to the story to make it complete.

We thank the Reviewer for these valid suggestions. The most accurate quantification related to this point is that of neutrophil crawling velocity as conducted using confocal intravital microscopy in conjunction with Imaris image analysis. This new data reveals slower crawling speed of KO neutrophils compared to WTs, further reinforcing our overall conclusion of aberrant deactivation of integrins in KO cells. Figure 4 has been updated to incorporate this result (new Figure 4B) as well as the quantification of neutrophil-derived fragments (new Figure 4C) which we believe is an outcome of firmly adherent cells trying to detach from venular walls.

3. Since they demonstrate that GPR84 has a role in multiple steps in the extravasation process, not just diapedesis, they should change the title to reflect that. I recommend, "Orphan GPR84 facilitates uropod de-adhesion at multiple steps in during (inflammatory) leukocyte extravasation."

We agree and have now changed the title accordingly to *"Orphan GPR84 facilitates uropod de-adhesion at multiple steps during leukocyte extravasation".*

4. Results lines 91 - 94 corresponding to Figs 4G, H: This sentence is confusing since it is hard to tell whether they use "breaching" to mean making a defect in the pericyte layer or to mean going through. Since they define this as trans-pericyte migration, it seems that they mean PMN migrating across the pericyte layer. However, then the "incomplete" TPM does not make sense. Please change the terms to make it clear what you mean.

The text has been edited for enhanced clarity in addition to change in Y axis label of Figure 4J.

Figure 1 legend, panel E. To truly show that the morphologies are distinct, it would be good to show WT leukocytes rendered in the same way.

We have now done this and included rendered leukocytes for all genotypes in Figure EV1.

Figure 1 legend, panel N. Are we supposed to see a wound. I do not see anything that appears green, but the box over the figure shows green as wound. Also, please point out the "vein" lumen in panels L, M, N.

In 1N, the wound is some distance away in this particular EM view. We have also labelled the vein lumen in L-N.

Dear Dr. Weavers,

Thank you for the submission of your revised manuscript to our editorial offices. I have now received the reports from the three referees that were asked to re-evaluate the study, you will find below. As you will see, the referees now fully support publication of your study in EMBO reports.

Before I can proceed with formal acceptance, I have the editorial requests below I ask you to address in a final revised manuscript. Please also provide a final p-b-p-response to the editorial requests.

Editorial requests:

- Please order the manuscript sections like this, using only these names:
Title page - Abstract - Keywords (max. 5) - Introduction - Results - Discussion - Methods - Data availability section - Acknowledgements - Disclosure and Competing Interests Statement - References - Figure legends - Expanded View Figure legends

Please remove the 'Short title' and the 'Summary' from the title page.

- Please provide the abstract written in present tense throughout.

- We now use CRediT to specify the contributions of each author in the journal submission system. CRediT replaces the author contribution section. Please use the free text box to provide more detailed descriptions and do NOT provide your final manuscript text file with an author contributions section. See also our guide to authors (section 'Author contributions'): https://link.springer.com/journal/44319/submission-guidelines#cms-Revised-submissions

- Please check again that the number "n" for how many independent experiments were performed, their nature (biological versus technical replicates), the bars and error bars (e.g. SEM, SD) and the test used to calculate p-values is indicated in the respective figure legends (main and EV figures). Please also check that all the p-values are explained in the legend, and that these fit to those shown in the figure. Please provide statistical testing where applicable. Please avoid the phrase 'independent experiment' but clearly state if these were biological or technical replicates. Please also indicate (e.g. with n.s.) if testing was performed, but the differences are not significant. In case n=2, please show the data as separate datapoints without error bars and statistics. See also:

https://link.springer.com/journal/44319/submission-guidelines#cms-Figure-and-data-presentation

If n<5, please show single datapoints for diagrams. Could statistics be provided for the diagrams in Fig. EV3? Moreover:
- Please note that the legends for figure 6 are not provided in a sequential manner (legend for figure 6D is provided before legend of figure 6C). This needs to be rectified.
- Please note that the exact p values are not provided in the legends of figures 1F,G,H; 3C,D,G; 4B,F,K; 5C; 7G,M,N; 8C-F,H,I; 9B-D,G,H; EV-1A,E; EV-4E.
- Please indicate the statistical test used for data analysis in the legends of figures 1G; 6B; EV-1A.
- Please note that information related to n is missing in the legends of figures 6B; 9G,H.
- Please note that the white asterisks are not defined in the legend of figure 1M; This needs to be rectified.
- Please note that the yellow asterisks are not defined in the legend of figure EV-1C; This needs to be rectified
- Please note that the white arrows are not defined in the legend of 7D-F; 8B; EV-1C. This needs to be rectified.

- As there are no other panels, please remove the label 'A' from figure EV3 and update any callouts.

- The data availability section (DAS) is restricted to information about large datasets generated in the study that have been deposited externally. Please remove any other information from this section.

- Please remove the legends for the movies from the manuscript text file. Please provide separate legends for each movie as text file ZIPped up with its respective movie file so that we have 7 folders (Movie EV1 - Movie EV7).

- Please use our reference format:
https://link.springer.com/journal/44319/submission-guidelines#cms-Reference-guidelines

Please use 'et al' after 10 author names; DOIs should only be used for preprints and datasets that have not been published yet.

- It seems that panels EV1B/C and EV4A/B show the same data, although this is not obvious from the legend (and the genotypes in 1C and 4B are supposed to be different). Please check. If this reuse is nevertheless intentional, please indicate this clearly in the respective figure legends.

In addition, I would need from you uploaded separately:
- a short, two-sentence summary of the manuscript (not more than 35 words).
- two to four short (!) bullet points highlighting the key findings of your study (two lines each).
- a schematic summary figure as separate file that provides a sketch of the major findings (not a data image) in jpeg or tiff format (with the exact width of 550 pixels and the exact height of 300 pixels) that can be used as a visual synopsis on our website. Please upload this separately and remove the graphical abstract from the manuscript text file.

I look forward to seeing the further revised version of your manuscript when it is ready. Please let me know if you have questions regarding the revision.

Best,

Achim Breiling
Senior Editor
EMBO Reports

Referee #1:

The authors have done fine work in addressing all my comments, and the manuscript is substantially stronger. I find the hemocyte-specific Tre1 rescue experiments in animals to be very compelling, as well as RhoA-activation downstream of the GPR-84 agonist. I fully support publication.

Referee #2:

The authors have adequately addressed my previous concerns. The new data and revision have further strengthened the manuscript. I have no further comments.

Referee #3:

The revision satisfactorily addresses all of my previous comments.

All minor editorial requests have been addressed by the authors.

2nd Revision - Editorial Decision 27th Aug 2026

Dr. Helen Weavers
University of Bristol
School of Biochemistry
University Walk
Bristol BS8 1TD
United Kingdom

Dear Dr. Weavers,

I am very pleased to accept your manuscript for publication in the next available issue of EMBO reports. Thank you for your contribution to our journal.

Your manuscript will be processed for publication by EMBO Press. It will be copy edited and you will receive page proofs prior to publication. Please note that you will be contacted by Springer Nature Author Services to complete licensing and payment information.

You may qualify for financial assistance for your publication charges - either via a Springer Nature fully open access agreement or an EMBO initiative. Check your eligibility: https://link.springer.com/journal/44319/how-to-publish-with-us

Should you be planning a Press Release on your article, please notify the production team via this form in order to coordinate publication and release dates: https://forms.cloud.microsoft/e/eH4A8JVP6g

If you have any questions, please do not hesitate to contact the Editorial Office. Thank you for your contribution to EMBO Reports.

Yours sincerely,

Achim Breiling
Senior Editor
EMBO Reports

>>> Please note that it is EMBO Reports policy for the transcript of the editorial process (containing referee reports and your response letter) to be published as an online supplement to each paper. If you do NOT want this, you will need to inform the Editorial Office via email immediately. More information is available here: https://link.springer.com/partners/embo-press/editorial-policies#Peer%20review

EMBO Press Author Checklist

Corresponding Author Name: Dr Helen Weavers	
Journal Submitted to: EMBO reports	
Manuscript Number: EMBOR-2026-63676V2	

USEFUL LINKS FOR COMPLETING THIS FORM
The EMBO Journal - Author Guidelines
EMBO Reports - Author Guidelines
Molecular Systems Biology - Author Guidelines
EMBO Molecular Medicine - Author Guidelines

Reporting Checklist for Life Science Articles (updated January
This checklist is adapted from Materials Design Analysis Reporting (MDAR) Checklist for Authors. MDAR establishes a minimum set of requirements in transparent reporting in the life sciences (see Statement of Task: 10.31222/osf.io/9sm4x). Please follow the journal's guidelines in preparing your
Please note that a copy of this checklist will be published alongside your article.

Abridged guidelines for figures
1. Data
The data shown in figures should satisfy the following conditions:
- the data were obtained and processed according to the field's best practice and are presented to reflect the results of the experiments in an accurate and unbiased manner.
- ideally, figure panels should include only measurements that are directly comparable to each other and obtained with the same assay.
- plots include clearly labeled error bars for independent experiments and sample sizes. Unless justified, error bars should not be shown for technical
- if n<5, the individual data points from each experiment should be plotted. Any statistical test employed should be justified.
- Source Data should be included to report the data underlying figures according to the guidelines set out in the authorship guidelines on Data

2. Captions
Each figure caption should contain the following information, for each panel where they are relevant:
- a specification of the experimental system investigated (eg cell line, species name).
- the assay(s) and method(s) used to carry out the reported observations and measurements.
- an explicit mention of the biological and chemical entity(ies) that are being measured.
- an explicit mention of the biological and chemical entity(ies) that are altered/varied/perturbed in a controlled manner.
- the exact sample size (n) for each experimental group/condition, given as a number, not a range;
- a description of the sample collection allowing the reader to understand whether the samples represent technical or biological replicates (including how many animals, litters, cultures, etc.).
- a statement of how many times the experiment shown was independently replicated in the laboratory.
- definitions of statistical methods and measures:
  - common tests, such as t-test (please specify whether paired vs. unpaired), simple χ2 tests, Wilcoxon and Mann-Whitney tests, can be unambiguously identified by name only, but more complex techniques should be described in the methods section;
  - are tests one-sided or two-sided?
  - are there adjustments for multiple comparisons?
  - exact statistical test results, e.g., P values = x but not P values < x;
  - definition of 'center values' as median or average;
  - definition of error bars as s.d. or s.e.m.

Please complete ALL of the questions below.
Select "Not Applicable" only when the requested information is not relevant for your study.

Materials

Newly Created Materials	Information included in the manuscript?	In which section is the information available? (Reagents and Tools Table, Materials and Methods, Figures, Data Availability Section)
New materials and reagents need to be available; do any restrictions apply?	Yes	Reagents and Tools, Materials and Methods

Antibodies	Information included in the manuscript?	In which section is the information available? (Reagents and Tools Table, Materials and Methods, Figures, Data Availability Section)
For **antibodies** provide the following information:<br>- Commercial antibodies: RRID (if possible) or supplier name, catalogue number and or/clone number<br>- Non-commercial: RRID or citation	Yes	Reagents and Tools, Materials and Methods

DNA and RNA sequences	Information included in the manuscript?	In which section is the information available? (Reagents and Tools Table, Materials and Methods, Figures, Data Availability Section)
**Short novel DNA or RNA including primers, probes**: provide the sequences.	Yes	Reagents and Tools table, Materials and Methods

Cell materials	Information included in the manuscript?	In which section is the information available? (Reagents and Tools Table, Materials and Methods, Figures, Data Availability Section)
**Cell lines:** Provide species information, strain. Provide accession number in repository **OR** supplier name, catalog number, clone number, and/**OR** RRID.	Not Applicable	
**Primary cultures:** Provide species, strain, sex of origin, genetic modification status.	Not Applicable	
Report if the cell lines were recently **authenticated** (e.g., by STR profiling) and tested for mycoplasma contamination.	Not Applicable	

Experimental animals	Information included in the manuscript?	In which section is the information available? (Reagents and Tools Table, Materials and Methods, Figures, Data Availability Section)
**Laboratory animals or Model organisms:** Provide species, strain, sex, age, genetic modification status. Provide accession number in repository **OR** supplier name, catalog number, clone number, **OR** RRID.	Yes	Reagents and Tools Table, Materials and Methods
**Animal observed in or captured from the field:** Provide species, sex, and age where possible.	Not Applicable	
Please detail **housing and husbandry conditions**.	Yes	Materials and Methods

Plants and microbes	Information included in the manuscript?	In which section is the information available? (Reagents and Tools Table, Materials and Methods, Figures, Data Availability Section)
**Plants:** provide species and strain, ecotype and cultivar where relevant, unique accession number if available, and source (including location for collected wild specimens).	Not Applicable	
**Microbes:** provide species and strain, unique accession number if available, and source.	Not Applicable	

Human research participants	Information included in the manuscript?	In which section is the information available? (Reagents and Tools Table, Materials and Methods, Figures, Data Availability Section)
If collected and within the bounds of privacy constraints report on age, sex and gender or ethnicity for all study participants.	Not Applicable	

Core facilities	Information included in the manuscript?	In which section is the information available? (Reagents and Tools Table, Materials and Methods, Figures, Data Availability Section)
If your work benefited from core facilities, was their service mentioned in the acknowledgments section?	Yes	Acknowledgements

Design

Study protocol	Information included in the manuscript?	In which section is the information available? (Reagents and Tools Table, Materials and Methods, Figures, Data Availability Section)
If study protocol has been **pre-registered, provide DOI in the manuscript**. For clinical trials, provide the trial registration number **OR** cite DOI.	Not Applicable	
Report the **clinical trial registration number** (at ClinicalTrials.gov or equivalent), where applicable.	Not Applicable	

Laboratory protocol	Information included in the manuscript?	In which section is the information available? (Reagents and Tools Table, Materials and Methods, Figures, Data Availability Section)
Provide DOI OR other citation details if **external detailed step-by-step protocols** are available.	Not Applicable	

Experimental study design and statistics	Information included in the manuscript?	In which section is the information available? (Reagents and Tools Table, Materials and Methods, Figures, Data Availability Section)
Include a statement about **sample size** estimate even if no statistical methods were used.	Yes	Materials and Methods
Were any steps taken to minimize the effects of subjective bias when allocating animals/samples to treatment (e.g. **randomization procedure**)? If yes, have they been described?	Yes	Materials and Methods
Include a statement about **blinding** even if no blinding was done.	Yes	Materials and Methods
Describe **inclusion/exclusion criteria** if samples or animals were excluded from the analysis. Were the criteria pre-established?<br><br>If sample or data points were omitted from analysis, report if this was due to attrition or intentional exclusion and provide justification.	Not Applicable	
For every figure, are **statistical tests** justified as appropriate? Do the data meet the assumptions of the tests (e.g., normal distribution)? Describe any methods used to assess it. Is there an estimate of variation within each group of data? Is the variance similar between the groups that are being statistically compared?	Yes	Figure Legends and Materials and Mathods

Sample definition and in-laboratory replication	Information included in the manuscript?	In which section is the information available? (Reagents and Tools Table, Materials and Methods, Figures, Data Availability Section)
In the figure legends: state number of times the experiment was **replicated** in laboratory.	Yes	Figure legends
In the figure legends: define whether data describe **technical or biological replicates**.	Yes	Figure legends

Ethics

Ethics	Information included in the manuscript?	In which section is the information available? (Reagents and Tools Table, Materials and Methods, Figures, Data Availability Section)
Studies involving **human participants**: State details of **authority granting ethics approval** (IRB or equivalent committee(s), provide reference number for approval.	Not Applicable	
Studies involving **human participants**: Include a statement confirming that **informed consent** was obtained from all subjects and that the experiments conformed to the principles set out in the WMA Declaration of Helsinki and the Department of Health and Human Services Belmont Report.	Not Applicable	
Studies involving **human participants:** For publication of **patient photos**, include a statement confirming that consent to publish was obtained.	Not Applicable	
Studies involving experimental **animals**: State details of **authority granting ethics approval** (IRB or equivalent committee(s), provide reference number for approval. Include a statement of compliance with ethical regulations.	Not Applicable	
Studies involving **specimen and field samples:** State if relevant **permits** obtained, provide details of authority approving study; if none were required, explain why.	Not Applicable	

Dual Use Research of Concern (DURC)	Information included in the manuscript?	In which section is the information available? (Reagents and Tools Table, Materials and Methods, Figures, Data Availability Section)
Could your study fall under dual use research restrictions? Please check biosecurity documents and list of **select agents and toxins** (CDC): https://www.selectagents.gov/sat/list.htm	Not Applicable	
If you used a select agent, is the security level of the lab appropriate and reported in the manuscript?	Not Applicable	
If a study is subject to dual use research of concern regulations, is the name of the **authority granting approval and reference number** for the regulatory approval provided in the manuscript?	Not Applicable	

Reporting

The MDAR framework recommends adoption of discipline-specific guidelines, established and endorsed through community initiatives. Journals have their own policy about requiring specific guidelines and recommendations to complement MDAR.

Adherence to community standards	Information included in the manuscript?	In which section is the information available? (Reagents and Tools Table, Materials and Methods, Figures, Data Availability Section)
State if relevant guidelines or checklists (e.g., **ICMJE, MIBBI, ARRIVE, PRISMA**) have been followed or provided.	Yes	Materials and methods
For **tumor marker prognostic studies,** we recommend that you follow the **REMARK** reporting guidelines (see link list at top right). See author guidelines, under 'Reporting Guidelines'. Please confirm you have followed these guidelines.	Not Applicable	
For **phase II and III randomized controlled trials**, please refer to the **CONSORT** flow diagram (see link list at top right) and submit the CONSORT checklist (see link list at top right) with your submission. See author guidelines, under 'Reporting Guidelines'. Please confirm you have submitted this list.	Not Applicable	

Data Availability

Data availability	Information included in the manuscript?	In which section is the information available? (Reagents and Tools Table, Materials and Methods, Figures, Data Availability Section)
Have **primary datasets** been deposited according to the journal's guidelines (see 'Data Deposition' section) and the respective accession numbers provided in the Data Availability Section?	Yes	Data Availability Section
Were **human clinical and genomic datasets** deposited in a public access-controlled repository in accordance to ethical obligations to the patients and to the applicable consent agreement?	Not Applicable	
Are **computational models** that are central and integral to a study available without restrictions in a machine-readable form? Were the relevant accession numbers or links provided?	Not Applicable	
If publicly available data were reused, provide the respective **data citations in the reference list**.	Not Applicable	
