# Supplementary material for: Orphan GPR84 facilitates uropod de-adhesion at multiple steps during leukocyte extravasation
Source: EMBO Rep. 2026 Sep 12;27(20):6018–49. doi: 10.1038/s44319-026-00923-0 (PMC13694715; doi:10.1038/s44319-026-00923-0)
Supplement: Supplementary file 3 [file 44319_2026_923_MOESM10_ESM.zip › Figure 2/2A_Tre1_Rhodopsin_phylogenetic_tree.pdf]

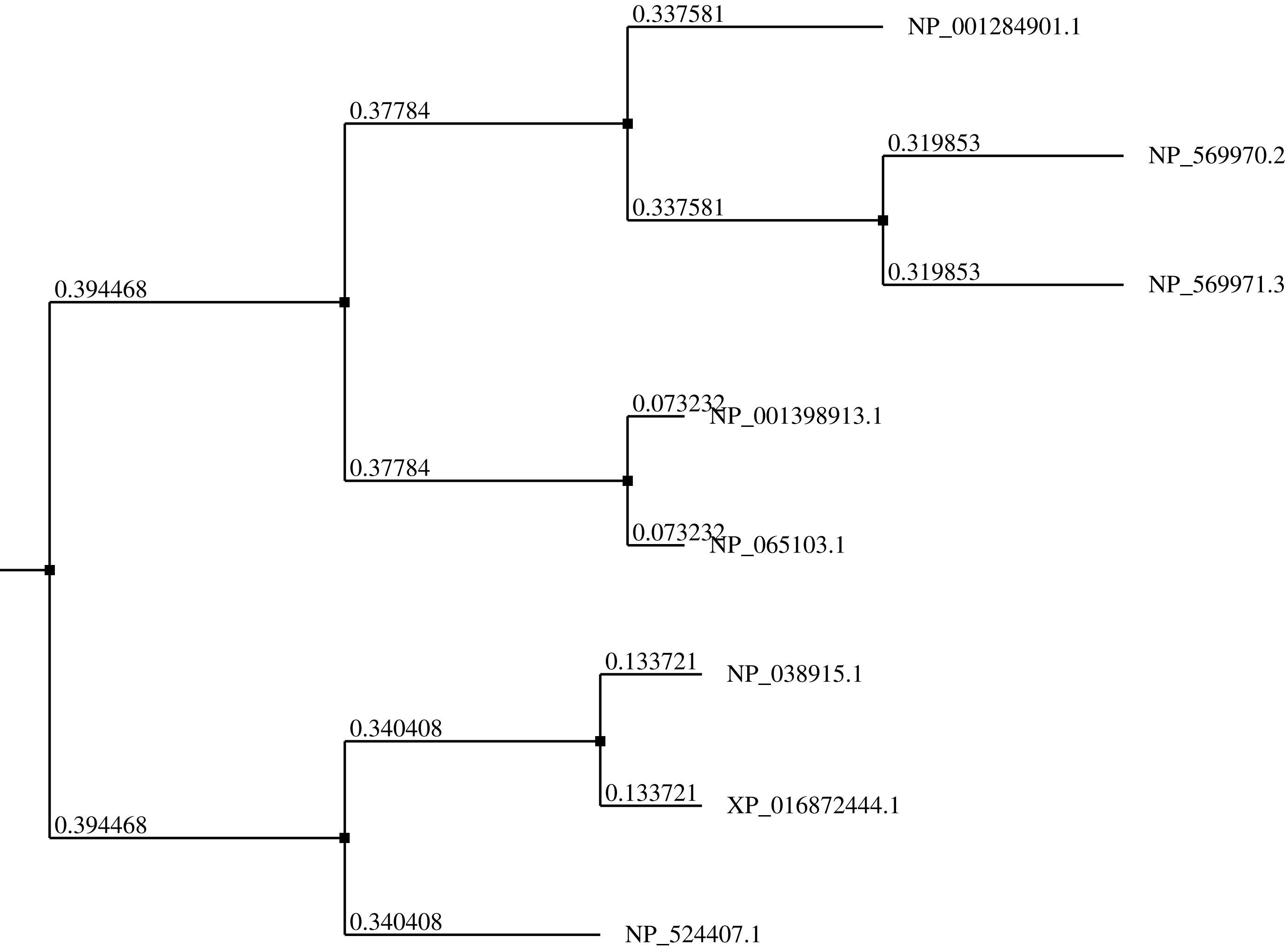
0.337581
NP_001284901.1
0.37784
0.319853
NP_569970.2
0.337581
0.394468
0.319853
NP_569971.3
0.073232
NP_001398913.1
0.37784
0.073232
NP_065103.1
0.133721
NP_038915.1
0.340408
0.133721
XP_016872444.1
0.394468
0.340408
NP_524407.1
