# Supplementary material for: Orphan GPR84 facilitates uropod de-adhesion at multiple steps during leukocyte extravasation
Source: EMBO Rep. 2026 Sep 12;27(20):6018–49. doi: 10.1038/s44319-026-00923-0 (PMC13694715; doi:10.1038/s44319-026-00923-0)
Supplement: Supplementary file 10 [file 44319_2026_923_MOESM2_ESM.zip › Movie EV1/Movie EV1.pdf]

Movie EV1. Hemocytes lacking Tre1 are impaired in extravasation. Related to Figure 1. Representative *in vivo* time-lapse imaging of 75h APF *WT* (left) and *Tre1*^{KO} (right) circulating *Drosophila* hemocytes responding and extravasating from pupal veins (blue dotted line) towards sites of laser-induced wounding (solid white line) Examples of extravasating hemocytes are highlighted with white arrows. Wing epithelium is labeled using ubiquitously expressed GMA (green), and hemocytes are labeled by both *srp-Gal4*–driven and the *srp* enhancer trap-driven expression of cytoplasmic mcherry (red). Scale bars represent 20 µm. Z-stacks were taken at regular 4.5 minute time intervals.
