# Supplementary material for: Orphan GPR84 facilitates uropod de-adhesion at multiple steps during leukocyte extravasation
Source: EMBO Rep. 2026 Sep 12;27(20):6018–49. doi: 10.1038/s44319-026-00923-0 (PMC13694715; doi:10.1038/s44319-026-00923-0)
Supplement: Supplementary file 11 [file 44319_2026_923_MOESM3_ESM.zip › Movie EV2/Movie EV2 .pdf]

Movie EV2. Tre1 vs Gpr84 share sequence and structural homology. Related to Figure 2. Structural comparison of Tre1 (maroon) and its human orthologue GPR84 (teal) with superimposed orthologues highlighting conserved structure. AlphaFold 3 and ChimeraX was used for orthologue structure comparisons.
