# Supplementary material for: Orphan GPR84 facilitates uropod de-adhesion at multiple steps during leukocyte extravasation
Source: EMBO Rep. 2026 Sep 12;27(20):6018–49. doi: 10.1038/s44319-026-00923-0 (PMC13694715; doi:10.1038/s44319-026-00923-0)
Supplement: Supplementary file 12 [file 44319_2026_923_MOESM4_ESM.zip › Movie EV3/Movie EV3 .pdf]

Movie EV3. *Gpr84*$^{-/-}$ mice show defective extravasation in TNF-stimulated cremaster muscles. Related to Figure 3. Representative brightfield intravital microscopy movies of post-capillary venule segments of TNF-stimulated cremaster muscles of WT and *Gpr84*$^{-/-}$ mice used to observe leukocyte responses (adhesion and extravasation). Arrows indicate extravasated neutrophils in the interstitial tissue at the 4 h experimental endpoint. Scalebars represent 50 μm.
