# Supplementary material for: Orphan GPR84 facilitates uropod de-adhesion at multiple steps during leukocyte extravasation
Source: EMBO Rep. 2026 Sep 12;27(20):6018–49. doi: 10.1038/s44319-026-00923-0 (PMC13694715; doi:10.1038/s44319-026-00923-0)
Supplement: Supplementary file 13 [file 44319_2026_923_MOESM5_ESM.zip › Movie EV4/Movie EV4 .pdf]

Movie EV4. *Gpr84*$^{-/-}$ mice display aberrant extravasation in TNF-stimulated cremaster muscles at the pericyte layer. Related to Figure 4. Representative confocal intravital microscopy movies of post-capillary venule segments of TNF-stimulated cremaster muscles from WT and *Gpr84*$^{-/-}$ on a *Lyz2-EGFP-ki*; *Acta2-RFPcherry-Tg* genetic background; endothelium (blue), pericytes (red) and neutrophils (green). The movie shows a post-capillary venule segment with high optical imaging of a neutrophil breaching the pericyte layer without pause in a WT (taking ~4 mins) and subsequently a neutrophil exhibiting multiple oscillations at the pericyte layer (*Gpr84*$^{-/-}$) throughout the 1 h imaging period. Scalebars represent 10 μm.
