# Supplementary material for: Orphan GPR84 facilitates uropod de-adhesion at multiple steps during leukocyte extravasation
Source: EMBO Rep. 2026 Sep 12;27(20):6018–49. doi: 10.1038/s44319-026-00923-0 (PMC13694715; doi:10.1038/s44319-026-00923-0)
Supplement: Supplementary file 14 [file 44319_2026_923_MOESM6_ESM.zip › Movie EV5/Movie EV5.pdf]

Movie EV5. ***Gpr84$^{-/-}$* mice display impaired detachment from acutely inflamed vessel walls.** Related to Figure 5. Representative confocal intravital microscopy movies of post-capillary venule segments of TNF-stimulated cremaster muscles of WT and *Gpr84$^{-/-}$* on a *Lyz2-EGFP-ki*; *Acta2-RFPcherry-Tg* genetic background; endothelium (blue) and neutrophils (green). Movie shows extravasated neutrophil displaying short uropods prior to detachment in a WT vessel segment. The movie then displays a *Gpr84$^{-/-}$* post-capillary venule segment with neutrophils exhibiting morphologies associated with impaired detachment. Specifically, arrows indicate the formation of elongated uropod structures, persistent uropods and spherical neutrophils displaying perivascular arrest over a 1 h imaging period. Scalebars represent 20 µm.
