# Supplementary material for: Orphan GPR84 facilitates uropod de-adhesion at multiple steps during leukocyte extravasation
Source: EMBO Rep. 2026 Sep 12;27(20):6018–49. doi: 10.1038/s44319-026-00923-0 (PMC13694715; doi:10.1038/s44319-026-00923-0)
Supplement: Supplementary file 15 [file 44319_2026_923_MOESM7_ESM.zip › Movie EV6/Movie EV6 .pdf]

Movie EV6. Hemocytes require Gαo for vein extravasation. Related to Figure 7. Representative *in vivo* time-lapse imaging of 75h APF control (left) and *srp-Gal4* driven *Gαo-RNAi* (right) circulating *Drosophila* hemocytes responding and extravasating from pupal veins (blue dotted line) towards sites of laser-induced wounding (solid white line). Hemocytes expressing *Gαo-RNAi* failed to fully extravasate from vessels over the 3 hours of imaging (right) and remained adhered to vein cells. Wing epithelium is labeled using GFP-tagged E-cadherin (green), and hemocytes are labeled by both *srp-Gal4*–driven and the *srp* enhancer trap expressing cytoplasmic mcherry (red). Scale bar represents 20 µm. Z-stacks were taken at regular 5 min time intervals.
