# Supplementary material for: Orphan GPR84 facilitates uropod de-adhesion at multiple steps during leukocyte extravasation
Source: EMBO Rep. 2026 Sep 12;27(20):6018–49. doi: 10.1038/s44319-026-00923-0 (PMC13694715; doi:10.1038/s44319-026-00923-0)
Supplement: Supplementary file 16 [file 44319_2026_923_MOESM8_ESM.zip › Movie EV7/Movie EV7.pdf]

Movie EV7. Tre1 is required for Rho1 localization at lagging tails of extravasating hemocytes. Related to Figure 7. Representative *in vivo* time-lapse imaging of 75h APF control (left) and *Tre1*^{KO} (right) *Drosophila* hemocytes extravasating from pupal veins (blue dotted line) towards sites of laser-induced wounding. Upon exit from veins, control hemocytes (left) develop a timely accumulation of active Rho1 that dissipates with de-adhesion to the vessel (white arrow). *Tre1*^{KO} hemocytes (right) fail to de-adhere with no observable active Rho1 accumulation at the tail. Active Rho1 is labelled using the Rho1 biosensor (Munjal et al. 2015), and hemocytes are labeled using the *srp* enhancer trap expressing cytoplasmic mcherry (red). Scale bars represent 5 µm. Z-stacks were taken at regular 20 s time intervals.
